# Semirigid Ligands Enhance Different Coordination Behavior of Nd and Dy Relevant to Their Separation and Recovery in a Non-aqueous Environment

**DOI:** 10.1021/acs.inorgchem.2c02619

**Published:** 2022-09-30

**Authors:** Alex Falco, Martina Neri, Matteo Melegari, Laura Baraldi, Giulia Bonfant, Matteo Tegoni, Angela Serpe, Luciano Marchiò

**Affiliations:** † Department of Chemistry, Life Sciences and Environmental Sustainability, University of Parma, Parco Area delle Scienze 17/A, 43124, Parma, Italy; ‡ Department of Civil and Environmental Engineering and Architecture (DICAAR) and Research Unit of INSTM, University of Cagliari, Via Marengo 2, 09123 Cagliari, Italy; § Environmental Geology and Geoengineering Institute of the National Research Council (IGAG-CNR), Piazza d’Armi, 09123 Cagliari, Italy

## Abstract

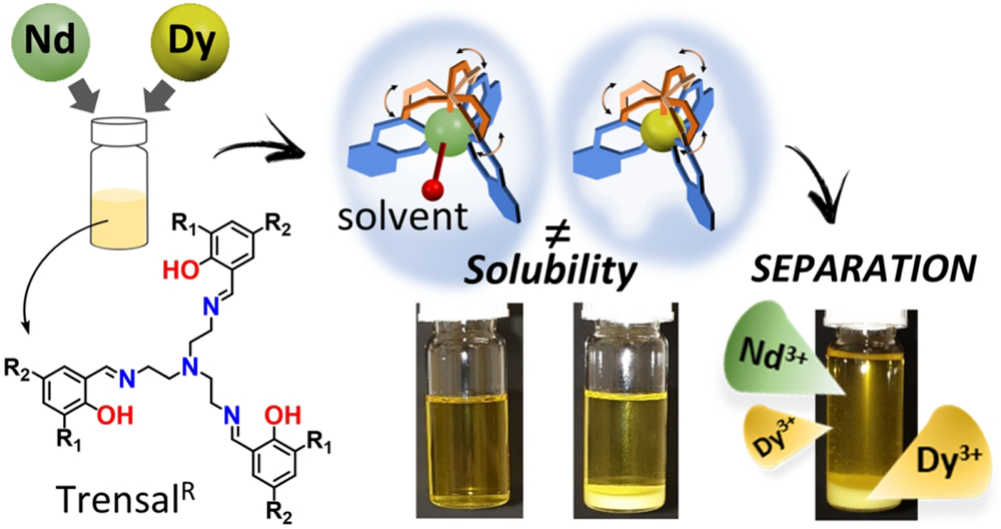

Rare-earth elements are widely used in high-end technologies, the production of permanent magnets (PMs) being one of the sectors with the greatest current demand and likely greater future demand. The combination of Nd and Dy in NdFeB PMs enhances their magnetic properties but makes their recycling more challenging. Due to the similar chemical properties of Nd and Dy, their separation is expensive and currently limited to the small scale. It is therefore crucially important to devise efficient and selective methods that can recover and then reuse those critical metals. To address these issues, a series of heptadentate Trensal-based ligands were used for the complexation of Dy^3+^ and Nd^3+^ ions, with the goal of indicating the role of coordination and solubility equilibria in the selective precipitation of Ln^3+^–metal complexes from multimetal non-water solutions. Specifically, for a 1:1 Nd/Dy mixture, a selective and fast precipitation of the Dy complex occurred in acetone with the Trensal^*p*-OMe^ ligand at room temperature, with a concomitant enrichment of Nd in the solution phase. In acetone, complexes of Nd and Dy with Trensal^*p*-OMe^ were characterized by very similar formation constants of 7.0(2) and 7.3(2), respectively. From the structural analysis of an array of Dy and Nd complexes with Trensal^R^ ligands, we showed that Dy invariably provided complexes with coordination number (cn) of 7, whereas the larger Nd experienced an expansion of the coordination sphere by recruiting additional solvent molecules and giving a cn of >7. The significant structural differences have been identified as the main premises upon which a suitable separation strategy can be devised with these kind of ligands, as well as other preorganized polydentate ligands that can exploit the small differences in Ln^3+^ coordination requirements.

## Abstract 2

Heptadentate Trensal^R^-based ligands were complexed with Dy^3+^ and Nd^3+^ cations. Dy compounds exhibited an invariable coordination number of 7. Nd complexes presented a coordination number of ≥8, with the metal having additional solvent or water molecules in the coordination sphere. The ligand Trensal^*p*-OMe^ led to a selective precipitation of the Dy complex in acetone, with a concomitant enrichment of Nd in the solution phase.

## Introduction

Magnets based on rare-earth elements (REEs) are among the most high-performing permanent magnets currently available. They are widely employed in wind and solar energy conversion and in hybrid and electric vehicles, transducers, HDD, speakers, cell phones, and many other appliances.^1,2^ NdFeB magnets owe their reputation to their remarkable magnetic properties such as a maximum energy product BH_max_ of 300 kJ/m^3^ and a coercivity _B_*H*_c_ of 900 kA/m. Indeed, rare-earth magnets along with hard ferrites account for a total of 90% of the market by value with a global annual consumption of REE estimated around 170 000 tons in 2020, which is expected to almost double by 2030.^3^ The rapid development of technologies for clean energy and transportation is significantly contributing to the growth in the demand for REEs, which has led to the identification of these metals as critical on a global scale because of the related supply risk, as pointed out by EU and U.S. Department of Energy assessments.^4,5^ At present, China is the global leader in the production and consumption of REEs, although export restrictions were introduced between 2010 and 2014, leading to a considerable growth in the price of rare-earth metals and a supply deficit to the rest of the world.^6^ Since then, the extent of global exploration for REE deposits has significantly increased and the recovery and recycling of rare-earth metals from End-of-Life magnets and industrial scraps have been taken into account as a supplement to the geological stocks.

Specifically, NdFeB PMs typically contain 28–35 wt % REEs (primarily Nd, with Pr, Tb, and Dy at lower rates) and represent one of the richest secondary sources of these metals, crucial for addressing the high risk of a supply shortage. The separation of a single lanthanide (Ln) over another metal of the group represents an actual challenge because of their similar chemical properties. Several methods have been developed to this end, primarily based on fractional precipitation,^7^ liquid–liquid extraction,^8−11^ and ion exchange chromatography,^12−14^ even if low selectivity and efficiency and the use of a large amount of hazardous and/or costly reagents and solvents often affect the technical, economical, and environmental sustainability of the processes. On the contrary, a relatively easier step involves the separation of the lanthanides from other transition metals. A variety of processes for separating REEs by the base metals contained in PMs have been developed in the past decade, often with a view of providing a REE-based mischmetal directly suitable for application. Among them, pyrometallurgical methods,^15^ selective molten salt leaching,^16,17^ and gas-phase extraction,^18^ in addition to conventional hydrometallurgical leaching and precipitation processes,^19^ can be cited. In this context, solvometallurgical approaches are rapidly emerging in the field of extractive metallurgy as an alternative to or combined with both hydrometallurgical and pyrometallurgical processes for a sustainable recovery of multiple metals, including REEs.^20−24^ Solvometallurgy is a suitable tool when water may interfere with materials and phases of the recycling process. In several cases, the use of non-water solvents may increase selectivity in metal coordination and separation, strongly affecting complex stability and solubility, with the result being improved effectiveness and reagent consumption. However, the development of sustainable solvometallurgical processes requires the employment of inexpensive, environmentally friendly solvents.^25^ Orefice and Binnemans showed that the protic ionic liquid (IL) pyridine hydrochloride can work at 165 °C as a nonselective leaching agent for waste NdFeB magnets and as a non-aqueous medium that is suitable for promoting a selective liquid–liquid extraction of the different lanthanides and metals contained in the scrap. The separation of neodymium and dysprosium was then achieved by the addition of an organophosphorous extractant (i.e., PC-88A) at different concentrations in non-aqueous solvents like *p*-cimene.^26^ Batchu et al. were able to separate europium and yttrium from ethylene glycol solutions by using the extractant Cyanex 923. They also demonstrated that the extraction from aqueous chloride solutions was inefficient compared to the non-aqueous process developed in the work.^27^

**Figure 1:**
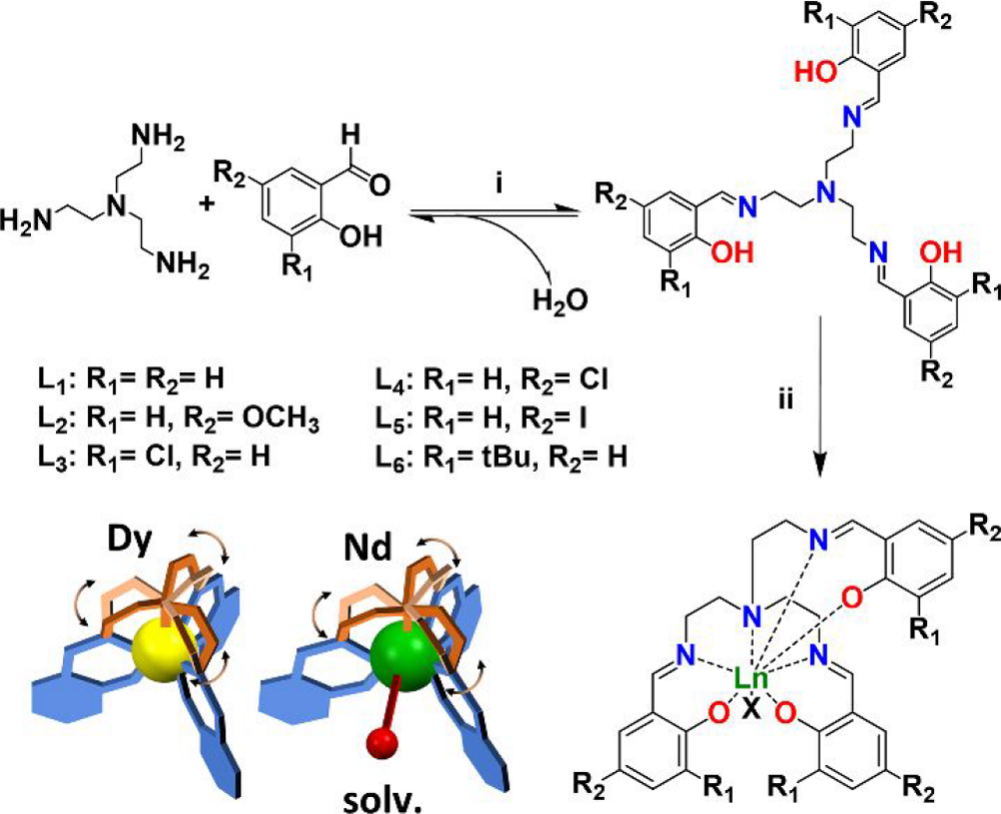
Synthesis of the ligands and coordination environment of the metals. Conditions: (i) tris(2-aminoethyl)amine (1 equiv), salicylic aldehyde^R^ (3 equiv), EtOH or CHCl_3_, Δ, 3 h; (ii) Trensal^R^ (1 equiv), Et_3_N (3 equiv), Ln(NO_3_)_3_·6H_2_O (1 equiv) (Ln = Dy or Nd), acetone or CH_3_CN, Δ, 1 h. The complexes are schematically represented highlighting the difference between the mobility of the −CH_2_–CH_2_– chain (brown) and the rigidity of the rest of the ligand (blue).

A valuable approach for the recovery of REEs is the use of multifunctional ligands, which can selectively separate lanthanide^28−31^ or actinide cations^32−35^ or a mixture of both.^36−42^ These ligands were specifically engineered to express different metal-to-ligand interaction geometries and strengths for a specific metal–ligand entity. In a slightly different approach, Bogart et al. showed that dysprosium and neodymium preferentially formed monomeric and dimeric entities, respectively, when using a polydentate N′N3O3 donor ligand. The monomer and the dimer exhibited different solubilities, which is relevant to an efficient REE separation.^31,43,44^ O’Connell-Danes, Love, and co-workers showed the selective precipitation and separation of lanthanide ions using a supramolecular strategy. In particular, a tripodal amido-arene synthon, in a biphasic HNO_3_/toluene system, could form a capsular structure hosting hexanitrometalate anions. The supramolecular assembly showed a lanthanide-dependent precipitation, thus leading to the selective separation of light from heavy lanthanide cations.^45^ Despite the similar chemical properties and ionic charges, different lanthanide ions may exhibit different coordination numbers (cn) and geometries when forming coordination compounds.^46^ The different structural features usually result in different physical properties, as in the case of compounds formed between various lanthanide ions and bidentate-functionalized 8-quinolinols in organic solvents.^47,48^ The family of tripodal ligands Trensal [H_3_trensal = 2,2′,2″-tris(salicylideneimino)triethylamine] is known to bind lanthanide +3 ions, and various studies have investigated the magnetic and optical properties of these complexes.^49−52^ By taking into account the fact that trivalent lanthanides present a cn of usually ≥7, one finds that the entropic effect plays a significant role in the formation of the complexes.^36^ In this case, the separation can be accomplished if the different complex stability is matched by a different solubility of the complexes. In this respect, the Trensal ligand can satisfy a coordination number of 7 (N′N3O3), with a suitable spatial orientation of the donor set for metal complexation. The ease of synthesis of Trensal allows for a different functionalization on its periphery, thus giving rise to the Trensal^R^ family (Figure 1). The ligand exhibits a rigid and preorganized arrangement of donor atoms in its periphery as well as a flexible ethylene linker connecting the central N and the donor moieties. These features allow for a slight, but significant, ability to adapt to the metal requirements (ion size and geometry) while preserving its full coordination properties. In a previous work, the effective Nd/Dy separation was investigated by using Trensal^*p*-Me^ as a chelating ligand.^53^ The slight difference in the stability constants between the Nd and Dy complexes was assessed as a reasonable explanation for their separation. However, the cited work did not report detailed structural information about the nature of the complexes, as it assumed (rightly, considering the nature of the ligand) the same isostructural seven-coordinated complexes for both metals. Here, we present the systematic synthesis of functionalized Trensal^R^ ligands, their reaction with Nd^3+^ and Dy^3+^ in organic solution, and a comprehensive investigation of the structural differences occurring between the obtained Nd and Dy complexes. In this study, solvometallurgy is preferred due to the low stability of the selected class of ligands in water. As a general observation, Nd complexes exhibit a cn of usually >7 by incorporating solvent molecules into the coordination sphere. On the contrary, Dy presents an invariant cn of 7. More interestingly, Trensal^*p*-OMe^ gave complexes with Dy and Nd characterized by a different solubility in acetone. We show here that when using a specific stoichiometric L:M ratio, we can maximize the separation of the two metals in acetone as the medium, obtaining a solid phase containing mostly Dy (82%) and a solution enriched with Nd (60%) in a single cycle. Even though the presented results do not show a complete separation between Nd and Dy and optimizations are still required to improve the conditions for a possible scale-up process, the results shed light on the different geometric and donor set requirements between Nd and Dy in the presence of a polydentate ligand. Indeed, the different speciation and structural features of the compounds formed with the two metals seem to be the most significant premises leading to a potential different solubility that can be ultimately helpful for their separation.

## Results and Discussion

### Synthesis of the Ligands and Complexes

The Trensal^R^ ligands used in this work were all synthesized in one step, starting from tris(2-aminoethyl)amine and the properly functionalized salicylaldehyde in a 1:3 stoichiometric ratio (Figure 1). A small excess of aldehyde was employed to selectively promote the formation of the three iminic functions. Ligands **L**_**1**_**–L**_**6**_ were characterized by nuclear magnetic resonance (NMR) and electrospray ionization mass spectrometry (ESI-MS) techniques as detailed in the Experimental Section and Figures S1–S12. Trensal is a heptadentate ligand having three phenolic oxygens, a central amine nitrogen, and the three imine nitrogen atoms. Indeed, the high denticity makes it suitable for the complexation of lanthanide ions, which, as hard species, show a marked preference for these types of ligands. In addition, these ligands can be easily prepared using aldehydes bearing diverse functional groups in the *ortho* and *para* position, many of them commercially available at a relatively low cost. The substituents were chosen to induce a different lipophilic character in the resulting ligand, with the aim of exploring the solubility of the ligands and complexes in various non-aqueous solvents. Furthermore, bulky groups as substituents may have a small but significant influence on the formation of the [Ln(Trensal^R^)] complex, upon comparison of Ln ions characterized by slightly different ionic radii such as Nd^3+^ (0.98 Å) and Dy^3+^ (0.91 Å).^54^ As a result, a possible variation in the coordination of metals (Nd vs Dy) may be anticipated, leading to the recruitment of additional donor atom moieties from the larger metal ion, with potential effects on solubility. An analogous scenario was previously described when using a polydentate N′N3O3 donor ligand, whose monomeric or dimeric entities exhibited different solubility.^31,43,44^

Even though the Trensal^R^ ligands are expected to be preorganized to host a Ln^3+^ metal center (Figures S45 and S46), in the initial part of this work we have prepared and structurally characterized the complex entities formed by the reaction in acetone or acetonitrile between Trensal^R^ ligands, lanthanide nitrates, and triethylamine in a 1:1:3 stoichiometric ratio (see Figure 1). The aim was to comprehensively investigate the behavior of each of the ligands with either Nd^3+^ or Dy^3+^. Ln(NO_3_)_3_ salts were selected as the inorganic precursors of Ln species to simulate leaching solutions typically provided by the most common hydrometallurgical processes, which often require dissolution steps of Ln-containing materials or process intermediates like REOs (rare-earth oxides) by nitric acid. Specifically, NdFeB waste magnets as well as mixtures of Ln_2_O_3_ obtained by preliminary separative treatments can be dissolved by HNO_3_ solutions before being concentrated and separated.^55^ Hence, a mixture of lanthanide nitrates may be considered as the feed solution for the present proof-of-concept work. Table 1 provides a summary of the different complexes of Nd and Dy obtained by the reaction of the lanthanide nitrate salts with Trensal^R^ ligands in the presence of triethylamine at the solvent during reflux for 1 h. The structures of all compounds were obtained by the recrystallization from DMF, except for those of compound **3**·acetone, **7**, and **13**. It is instructive to compare the structural arrangements of the Dy and Nd complexes as detailed in the next section, because this analysis will provide information about factors underlying the different solubilities of some of the complexes.

**Table 1:** Summary of the Ln(Trensal^R^) Complexes^a^

ligand	complex
Trensal	[Dy(Trensal)] (**1**)^b^
	[Nd(Trensal)(H_2_O)] (**7**)^b^
	[Nd(Trensal)]·ACN (**8**·ACN)^b^
Trensal^*o*-Cl^	[Dy(Trensal^*o*-Cl^)] (**2**)
	[Nd(Trensal^*o*-Cl^)(DMF)]·DMF (**11**·DMF)
Trensal^*o*-tBu^	[Dy(Trensal^*o*-tBu^)]·acetone (**3**·acetone)
	[Nd(Trensal^*o*-tBu^)]·DMF (**10**·DMF)
Trensal^p-I^	[Dy(Trensal^*p*-I^)]·3DMF (**4**·3DMF)
	[Nd(Trensal^*p*-I^)(DMF)(H_2_O)]·DMF (**9**·DMF)
Trensal^*p*-oMe^	[Dy(Trensal^*p*-OMe^)] (**5**)
	[Nd(Trensal^*p*-OMe^)(H_2_O)] (**13**)
Trensal^*p*-Cl^	[Dy(Trensal^*p*-Cl^)] (**6**)
	[Nd(Trensal^*p*-Cl^)(DMF)(H_2_O)] (**12**)

a The synthesis was performed in acetone.

b The synthesis was performed in acetonitrile.

### Description of [Ln(Trensal)] Crystal Structures

**Figure 2:**
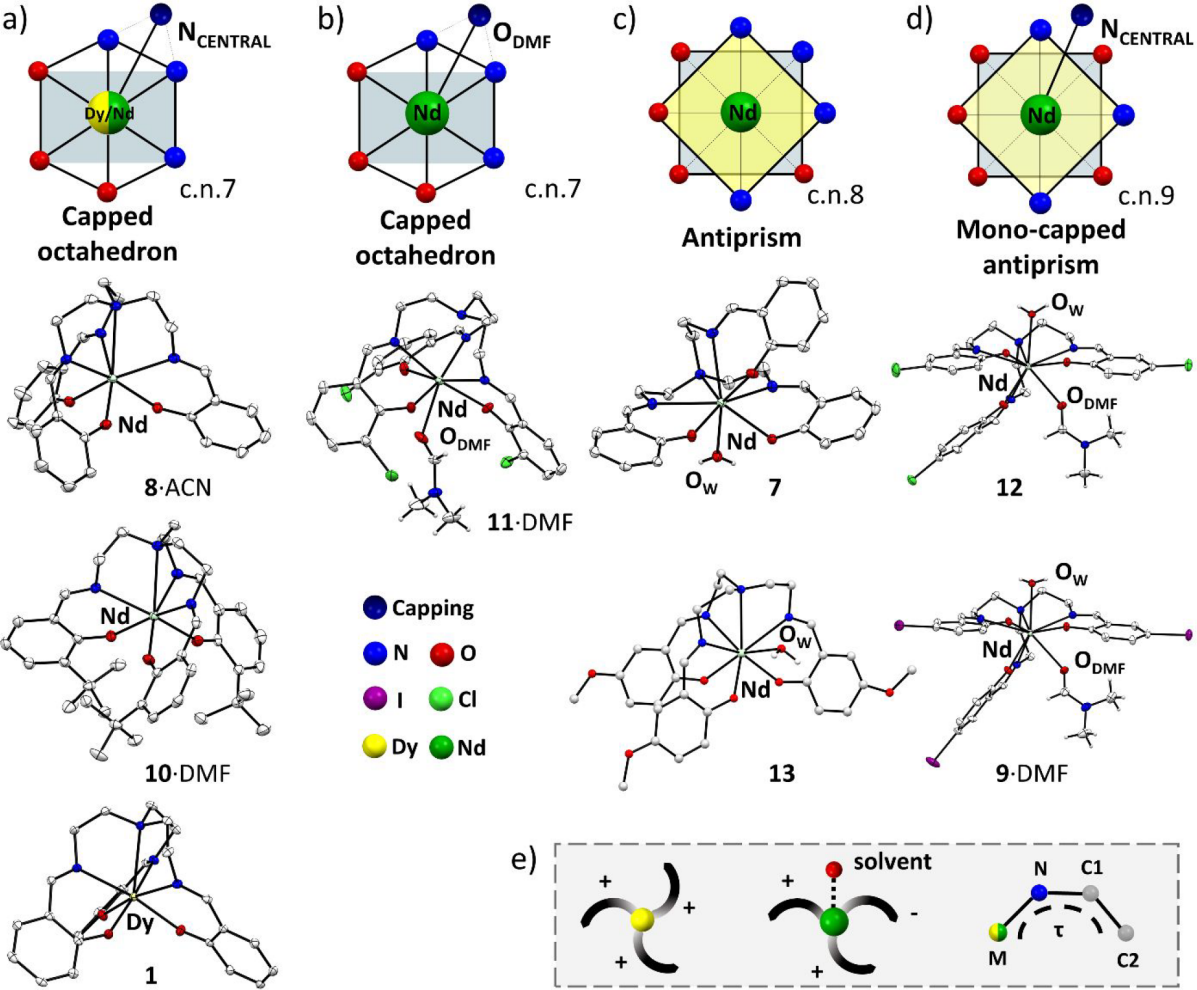
Molecular structures of selected complexes, together with a schematic representation of the metal geometry. (a) [Nd(Trensal)]·ACN (**8**·ACN), [Nd(Trensal^*o*-tBu^)]·DMF (**10**·DMF), and [Dy(Trensal)] (**1**) as representatives with cn values of 7. (b) [Nd(Trensal^*o*-Cl^)(DMF)]·DMF (**11**·DMF). (c) [Nd(Trensal)(H_2_O)] (**7**) and [Nd(Trensal^*p*-OMe^(H_2_O)] (**13**). (d) [Nd(Trensal^*p*-Cl^)(DMF)(H_2_O)] (**12**) and [Nd(Trensal^*p*-I^)(DMF)(H_2_O)]·DMF (**9**·DMF). The solvent of crystallization and most of the H atoms have been omitted for the sake of clarity. (e) Different orientations of the three arms of the ligands as a function of the sign of the τ angle (M–N_central_–C1–C2).

**Figure 3:**
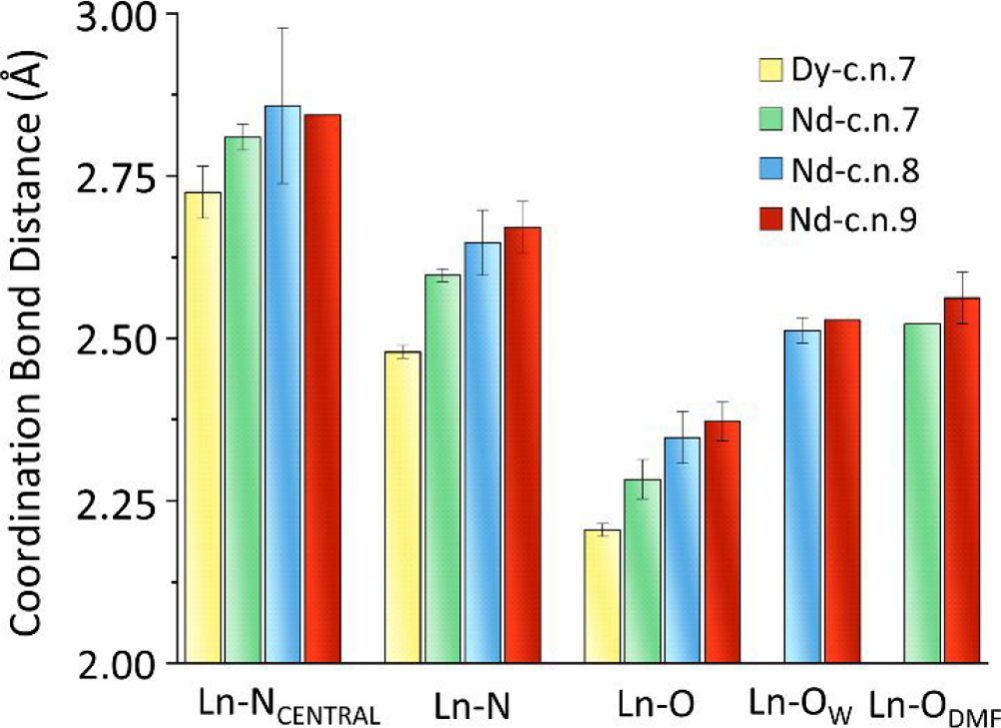
Mean values of the coordination bond distances as a function of cn, metal type, and donor atom.

The structural characterization of the complexes was an essential step in the interpretation of the different properties of the compounds and in the rational design of the next generations of ligands (Figures 2 and 3). Details of the structural investigation of [Dy(Trensal)] (**1**), [Dy(Trensal^*o*-Cl^)] (**2**), [Dy(Trensal^*o*-tBu^)]·acetone (**3**·acetone), [Dy(Trensal^*p*-I^)]·3DMF (**4**·3DMF), [Dy(Trensal^*p*-OMe^)] (**5**), [Dy(Trensal^*p*-Cl^)] (**6**), [Nd(Trensal)(H_2_O)] (**7**), [Nd(Trensal)]·ACN (**8**·ACN), [Nd(Trensal^*p*-I^)(DMF)(H_2_O)]·DMF (**9**·DMF), [Nd(Trensal^*o*-tBu^)]·DMF (**10**·DMF), [Nd(Trensal^*o*-Cl^)(DMF)]·DMF (**11**·DMF), [Nd(Trensal^*p*-Cl^)(DMF)(H_2_O)] (**12**), and [Nd(Trensal^*p*-OMe^)(H_2_O)] (**13**) are reported in Tables S2–S8. Dy complexes exhibit an invariant molecular structure with respect to the metal environment. Hence, compounds **1–6** will be described together. The metal coordination is capped octahedral, with the three phenoxy oxygen atoms and three imine nitrogen atoms that occupy the vertices of the octahedron, and the tertiary aminic nitrogen atom comprising the seventh coordination site (Figure 2a and Figures S19–S30). Consistently, the Dy–N_central_ distance [2.674(3)–2.766(7) Å] is significantly longer than the other Dy–N bond distances [2.460(2)–2.503(4) Å]. The Dy–O bond distance [2.187(3)–2.223(2) Å] is the shortest in the coordination environment, in agreement with the presence of negative charge on the oxygen atom. On the contrary, when the coordinated metal is Nd, the complexes show diverse molecular structures and usually show the presence of solvent molecules of crystallization interacting with the metal center. In more detail, only in **8**·ACN and **10**·DMF is the metal coordination capped octahedral and similar to that of the Dy complexes (Figures S33, S34, S37, and S38). However, the coordination bond distances are longer in the Nd complexes, in agreement with the larger size of Nd^3+^ with respect to Dy^3+^. A direct comparison is possible because **8**·ACN, **10**·DMF, and the Dy complexes have the same cn of 7 (see Figure 2a). The Nd–N_central_ distance [2.798(2)–2.821(4) Å] is significantly longer than the other Nd–N bond distances [2.596(1)–2.601(4) Å], with the Nd–O bond distances being the shortest [2.248(3)–2.273(1) Å].

The metal coordination in **11**·DMF is capped octahedral, obtained by three phenoxy oxygen atoms and three imine nitrogen atoms that define the octahedron, with the oxygen of the coordinated DMF that represents the capped position (Figure 2b and Figures S39 and S40). The Nd–N_central_ distance is too long [3.012(2) Å] to contribute to the metal coordination irrespective of the proper orientation of the lone pair. In addition, the DMF coordinated at the metal is disordered over sites with equivalent occupancy, which does not alter the overall complex geometry. The Nd–O_DMF_ distance [2.522(4) Å] is shorter than the Nd–N bond distances [2.595(2)–2.622(2) Å] but longer than the other Nd–O bond distances from the phenoxy groups [2.303(1)–2.337(1) Å], which remain the shortest in the coordination environment.

The metal coordination in **7** and **13** is antiprismatic, achieved by three phenoxy oxygen atoms, three imine nitrogen atoms, the central N of the ligand, and one molecule of water (Figure 2c and Figures S31, S32, S43, and S44). The Nd–N_central_ distance [2.776(2) Å for **7** and 2.82(2) Å for **13**] is the longest, followed by the other Nd–N bond distances [2.583(2)–2.677(2) Å for **7** and 2.58(3)–2.72(2) Å for **13**] and by the Nd–O_water_ one [2.523(2) Å for **7** and 2.49(2)–2.53(2) Å for **13**]. The shortest distances are those involving the phenoxy groups [2.299(2)–2.410(2) Å for **7** and 2.24(2)–2.38(2) Å for **13**].

**9**·DMF and **12** exhibit a capped antiprismatic metal coordination, where the seven coordination sites provided by the ligand are complemented by a water and a DMF molecule. The central N atom of the ligand occupies the capping position (Figure 2d and Figures S35, S36, S41, and S42). In both complexes, the Nd–N_central_ distance [2.841(3) Å for **9**·DMF and 2.846(2) Å for **12**] is significantly longer than the other Nd–N bond distances [2.647(3)–2.708(3) Å for **9**·DMF and 2.626(2)–2.733(2) Å for **12**], followed by the Nd–O_Water_ [2.531(3) Å for **9**·DMF and 2.525(1) Å for **12**] and Nd–O_DMF_ [2.588(3) Å for **9**·DMF and 2.535(1) Å for **12**] distances. As expected, the shortest distances are those involving the phenoxy groups [2.352(3)–2.397(2) Å for **9**·DMF and 2.349(1)–2.410(1) Å for **12**]. In addition to the structures presented here, it was previously reported that Nd and Trensal^*p*-Cl^ can form the [Nd(Trensal^*p*-Cl^)(H_2_O)] complex, having an antiprismatic geometry analogous to that of **7** and **13**.^56^

From the structural analysis, it is evident that two general features can be derived by the comparison of the Dy and Nd systems. The larger size of Nd leads to an increase in the bond distances pertaining to the coordination environment with respect to the Dy cases (Figure 3). As a result, the Nd atom is generally more exposed to the solvent and can therefore increase the cn from 7 to 9. The presence of the solvent (water or DMF) bound to Nd is particularly interesting because it offers additional interaction sites toward the bulk of the solvent, thus altering the solubility properties of the compounds. The ligands under investigation are characterized by a 3-fold symmetry and exhibit conformationally rigid coordination moieties (functionalized phenyl rings) connected to the central atom through the more flexible ethylene linkers. We introduce torsion angle τ (M–N_central_–C1–C2) to highlight additional differences between the geometry of Dy and Nd complexes. In particular, Dy shows a conserved 3-fold symmetry (τ angles having the same sign), whereas Nd shows more structural variability. Indeed, whenever a water or solvent molecules are bound to the Nd (**7**, **9**·DMF, **12**, and **13**), the 3-fold symmetry is disrupted (Figure 2e and Figure S47).

**Figure 4:**
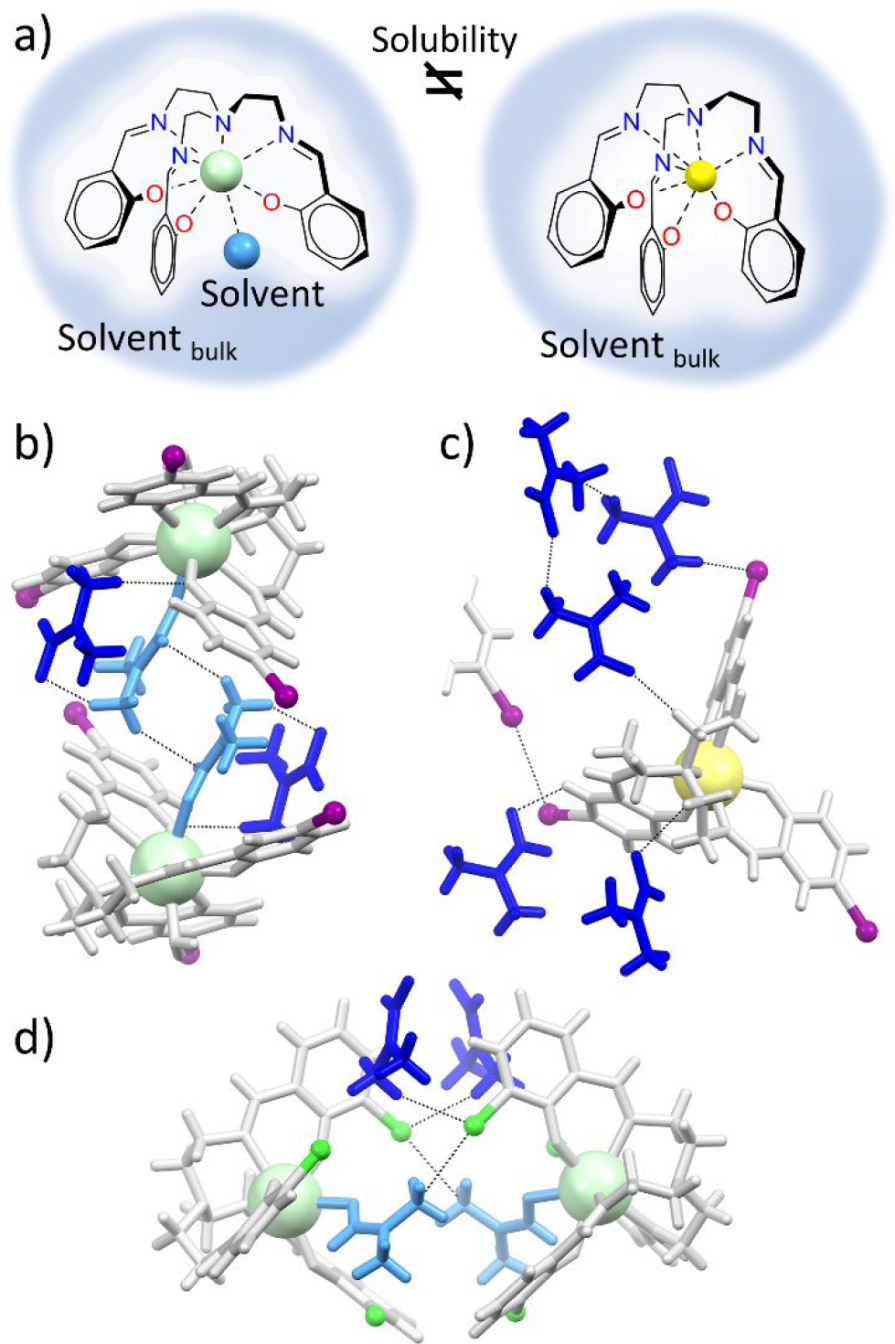
(a) Description of the potential influence of the coordinated solvent molecules on the Nd and Dy complexes on solubility. (b–d) Interactions exchanged by the coordinated solvent (light blue) and by the solvent of crystallization (blue) with the complex molecules in **9**·DMF, **4**·3DMF, and **12**. Nd is colored light green, and Dy yellow.

Another interesting feature worth noting is the different peripheral functionalization of the phenoxy groups of the ligands, as well as its influence on the interactions that are exchanged within the lattice. Indeed, the presence of iodine atoms, chlorine atoms, or methoxy groups in some of the ligands promotes the formation of weak interactions between the complex molecules and adjacent molecules or solvent of crystallization (Figure 4 and Figures S26, S36, and S42). More specifically, iodine atoms can act as halogen bond donors toward a nucleophile system such as the oxygen atom of DMF, whereas the methoxy group can exchange weak hydrogen bonds with aromatic or aliphatic CH moieties. These observations are suggestive of a potential different solubility of Nd complexes versus Dy complexes, according to the different interaction that a single molecular entity can exchange with a bulk solvent such as acetone, which is endowed with, though weak, coordination ability, as well as potentially acting as a halogen and hydrogen bond acceptor.

### Solution Studies and Nd/Dy Separation

**Figure 5:**
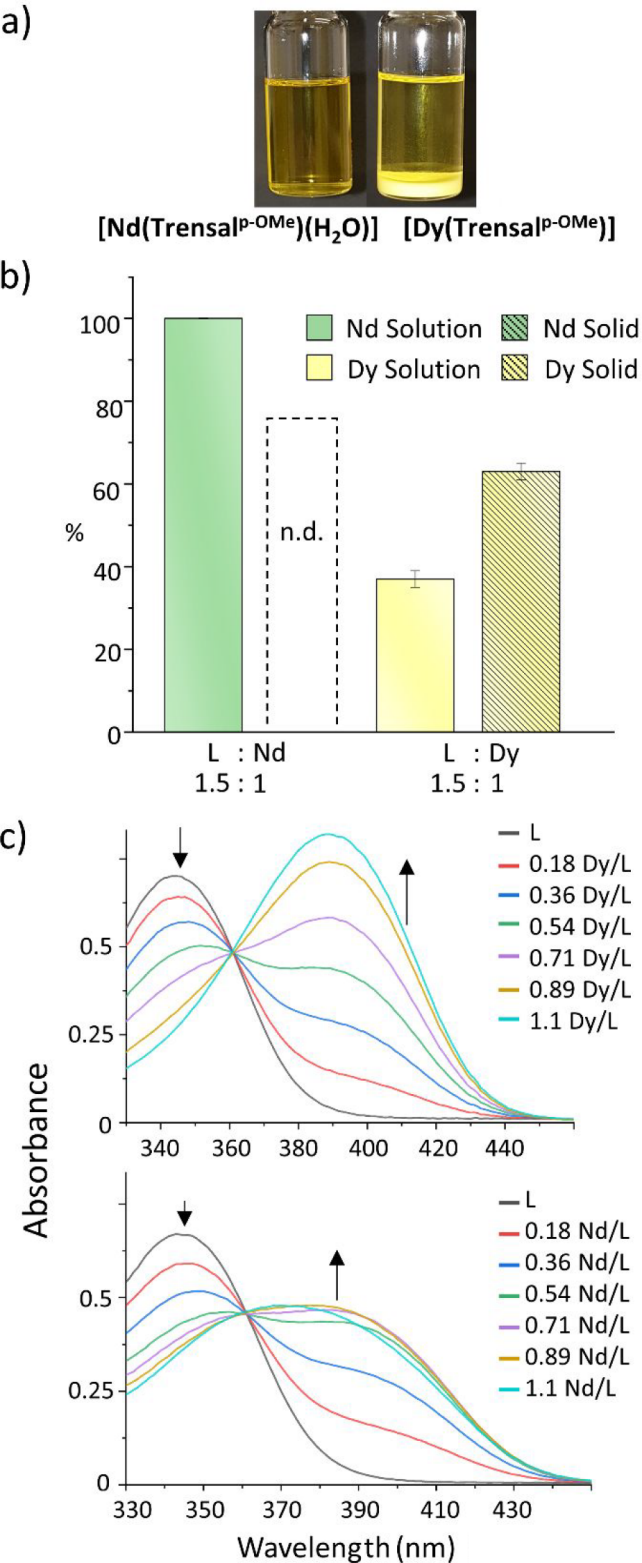
(a) Vials containing Nd and Dy nitrates mixed with Trensal^*p*-OMe^ in acetonitrile, showing the different solubility of the [Nd(Trensal^*p*-OMe^)(H_2_O)] (left) and [Dy(Trensal^*p*-OMe^)] (right) complexes. *C*_ligand_ = 1.16 × 10^–2^ M. *C*_Nd_ = *C*_Dy_ = 3.87 × 10^–3^ M. *C*_triethylamine_ = 3.48 × 10^–2^ M. (b) Distribution of Nd and Dy in the solid and solution phases in the presence of Trensal^*p*-OMe^ in acetone after 3 h, as measured by ICP-AES. (c) Titration experiments. UV–vis spectra recorded in acetone by increasing the metal:ligand ratio as indicated (*C*_ligand_ = 6.28 × 10^–5^ M, 1:3 ligand:triethylamine, 1:0–1.1 L:Ln).

Ligands **L**_**1**_**–L**_**6**_ used in this work were identified for their good lipophilicity, in agreement with the theoretical partition coefficients (logP) (Table S1), and they exhibit good solubility in non-aqueous environments. It is worth noting that the ligand was not stable in aqueous medium. Although the ligand was only sparingly soluble in water at neutral pH, we could obtain 5.63 × 10^–5^ M solutions of the ligand under buffered conditions between pH 4.4 and 10.1. For all of these solutions, a dramatic change in the absorption features was observed within seconds or minutes. We have interpreted these changes with hydrolysis of the Shiff base groups and have excluded water as a possible solvent for further studies of these systems. Accordingly, previous studies by Masuya-Suzuki et al. on Trensal^*p*-Me^ were performed in dried DMSO/isopropyl alcohol mixtures.^53^ From a screening of various organic solvents, acetone was selected for the separation experiments because all of the ligands were highly soluble in it, and the stock solutions were stable for at least up to a month in a nondry environment (Figure S13). Interestingly, we found that the presence of adventitious water molecules in acetone did not induce a degradation of the ligands with time (Figure S14). Furthermore, among the volatile organic compounds (VOCs), acetone is generally recognized as belonging to the “preferred” group of green solvents and widely industrially used, due to its properties, low cost, environmental impact, and health hazard.^57,58^ Finally, its low boiling point, despite growing concerns about its use, makes acetone very easy to recycle, limiting or avoiding process wastewater production and favoring the collection of products from the leaching solutions. Hence, systematic experiments performed in acetone with the different ligands demonstrated the peculiar capability of the Trensal^*p*-OMe^ ligand to react with Nd^3+^ and Dy^3+^ forming complexes with a marked solubility difference in this solvent. When nitrate salts of Nd or Dy were mixed with the ligand (10^–2^ M) in a 1:3 ratio in acetone at room temperature, only the precipitation of the [Dy(Trensal^*p*-OMe^)] complex in a few minutes was observed (Figure 5a) (a video with the time line of the precipitation of the complexes is available as Supporting Information). We then measured the amount of Nd and Dy both in solution and in the solid phase for the single Nd and Dy complexes in acetone and after 3 h (Figure 5b). At the concentration investigated and using an excess of ligand (*C*_Ln_ = 7.73 × 10^–3^ M; *C*_Trensal^*p*-OMe^_ = 1.16 × 10^–2^ M; *C*_triethylamine_ = 3.48 × 10^–2^ M; 1:1.5 M:L ratio), the Nd complex was completely soluble in acetone while the Dy complex was partially soluble (see Figure S17). For the Dy system, we determined the amount of Dy complex in the solid phase at three different temperatures (0, 25, and 55 °C). The precipitation started instantaneously after the addition of the metal to the solution of the ligands in the presence of triethylamine, and the analysis was performed after 3 h to maximize the precipitation. The weight percent of the recovered metal from the solid phase followed the order 80% (0 °C), 79% (25 °C), and 63% (55 °C), indicative of a slight negative correlation between temperature and precipitation. At 55 °C, the Dy^3+^ concentration in acetone was quantified as 2.86 × 10^–3^ M. In addition, we investigated formation of the complex through the evolution of the corresponding ultraviolet–visible (UV–vis) spectra. For this purpose, it was necessary to decrease the concentration of the reagents to prevent complex precipitation. Specifically, keeping the concentration of the ligands below 6.28 × 10^–5^ M and adding ≤1.1 equiv of metal ion in the presence of 3 equiv of triethylamine, we found no precipitation occurred. The UV–vis spectra of samples containing Trensal^*p*-OMe^ to which were added increasing amounts of Nd and Dy are represented in Figure 5c. For both metals, the absorbance of the band at ∼340 nm of the ligand diminished in favor of an increase in the absorbance of the band at 390 nm (which for Nd appears as a shoulder/broad band). In these spectra, the presence of the isosbestic point at 360 nm supported the hypothesis that upon addition of ≤1 equiv of metal the 1:1 lanthanide/ligand complexes were the only species formed in solution. Treatment of the spectral data using the HypSpec2014 software allowed us to determine the values of the logarithms of the formation constants (*K*) of the 1:1 Nd/L and Dy/L complexes, which were 7.0(2) and 7.3(2), respectively. These values are 1 to 2 log units higher than those determined by Masuya-Suzuki et al.,^53^ who reported the formation constants in dimethyl sulfoxide for Dy and Nd complexes with Trensal^*p*-Me^. Indeed, acetone exhibits a weaker solvation effect toward the metals with respect to dimethyl sulfoxide, thus explaining the larger values of *K* in acetone. However, it is worth noting that the difference between the two log *K* values (0.2 log unit) was not significant, so that we could assume that the two systems, 1:1 Nd/L and Dy/L, exhibited the same thermodynamic stability. Similar titrations in acetonitrile were also performed (Figure S15), and the results were consistent with those reported in Figure 5c.

Because of the molecular structures reported and the titration experiments, we expected that the complexation would lead to the formation of mononuclear entities, which, in the case of Nd, would usually incorporate solvent molecules into the coordination sphere. Despite the difference in the cn between the Nd and Dy species, the heptadentate ligands of the Trensal family conferred a similar stability to the resulting complexes. Hence, thermodynamic stability may not be a determining factor for the selective separation of the different complex species having two different metals. Here, we showed that the difference in the behavior of the metal/Trensal^*p*-OMe^ complexes in solution is strictly related to their diverse molecular structures, as the solvent molecules embedded in the coordination sphere of the Nd complex allegedly promoted the formation of further interactions with the bulk of the solution phase.

**Figure 6:**
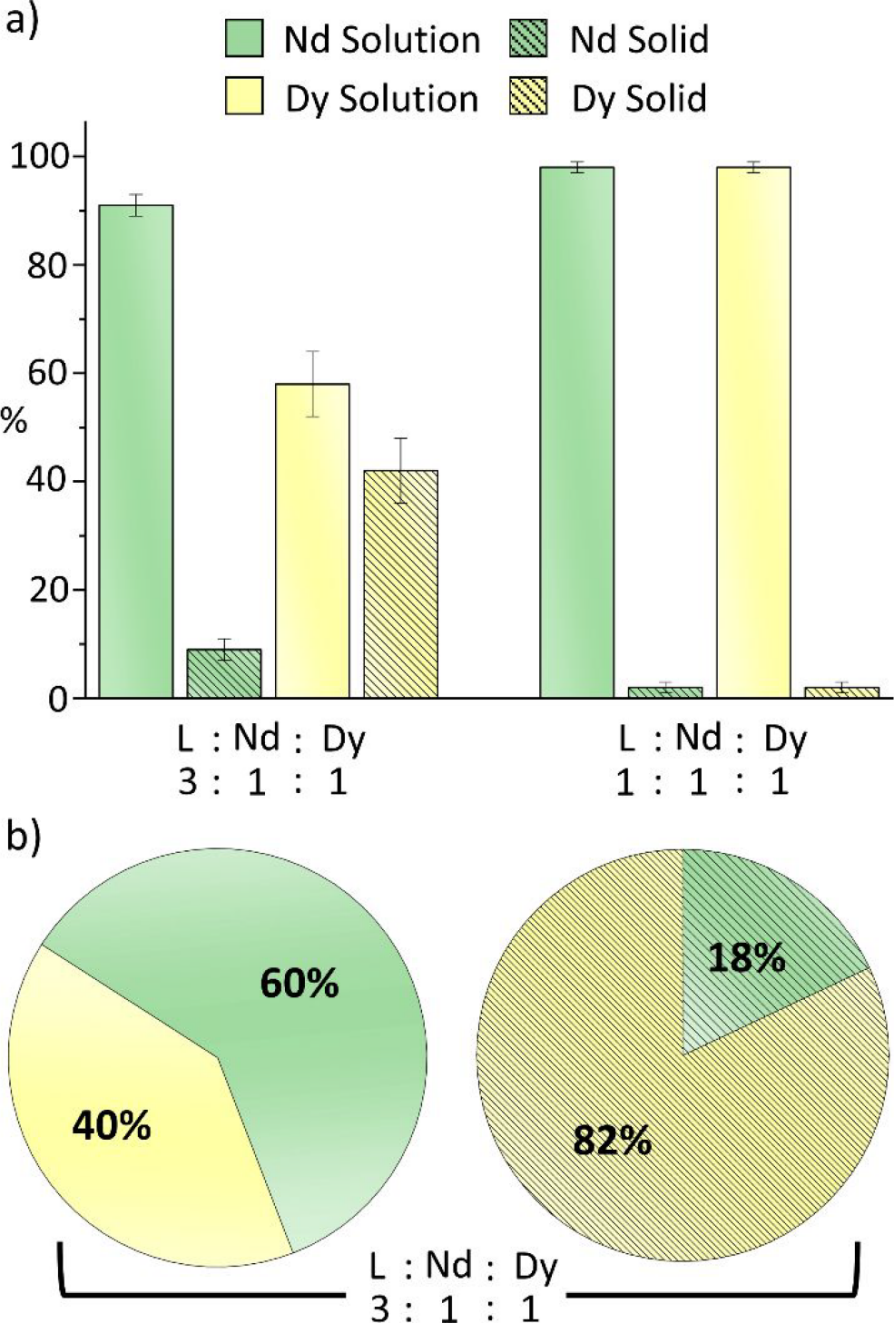
ICP-AES results. (a) Weight percent of Nd and Dy recovered from the solution and solid phases for two different stoichiometric ratios in acetone after mixing for 3 h Nd^3+^, Dy^3+^, Trensal^*p*-OMe^ (L), and triethylamine in 1:1:3:9 and 1:1:1:3 stoichiometric ratios, respectively. The metal cations were added as nitrate salts. (b) Weight percent of Dy and Nd in the liquid and solid phases.

The separation efficiency using Trensal^*p*-OMe^ was assessed by mixing a 1:1 solution of Nd and Dy (*C*_Nd_ = *C*_Dy_ = 3.87 × 10^–3^ M) and a ligand solution in various ratios from a substoichiometric ratio to an excess of ligand (1:1:3, 1:1:2, 1:1:1, and 1:1:0.75 Nd:Dy:L) in the presence of 3 equiv of triethylamine with respect to the ligand (Figure 6 and Figure S49). The first case represented a situation in which the ligand was in excess with respect to the metals and the separation efficiency should be determined by the different solubility properties of the two complexes, and assuming a very similar thermodynamic stability for the Nd and Dy complexes. In the last two cases (ligand in defect), the separation efficiency might be partly determined by the eventual different stability of the complexes. Indeed, with the ligand in defect, a different stability may result in different concentrations of the complexes in solution for the same concentrations of ligand and metals, in turn resulting in an overall different solubility.

The separation experiments showed that the maximum separation can be achieved by using an at least 1.5-fold excess of ligand with respect to the single cation (1:1:3 Nd:Dy:L). On the contrary, when the concentration of the ligand was decreased from stoichiometric to substoichiometric ratios, there appeared to be no discrimination between the two different metals because the solution and recovered solid phases, and for each individual phase, contained approximately the same amount of the two metals (Figure 6 and Figure S49). Interestingly, and unexpectedly because of the single solubility experiments, when both metals were present together with the ligand, there was a concomitant precipitation of the Nd complex as a minor component together with the Dy one. For the 1:1:3 Nd:Dy:L ratio, the weight percents of Dy and Nd in the solid phase were quantified as 82% and 18%, respectively. Consistently, the weight percents in the solution phase for Dy and Nd were quantified as 40% and 60%, respectively. This data showed an enrichment of the Nd in the solution phase and a significant enrichment of Dy in the solid phase.

The different solubility was most likely due to the different molecular structures and to the expansion of the coordination number in the Nd complex, which promoted an additional interaction with the surrounding solvent. Because of the stability constants determined in acetone, in Figure S50 we report the speciation of the systems with 1:1:1–3 Nd:Dy:L ratios at metal concentrations of 3.5 and 0.35 mM, with 3 equiv of base with respect to the ligand. The analysis involved systems in which the ligand:metal ratio varied from stoichiometric to substoichiometric (i.e., in excess of total lanthanide vs ligand). The plotted data clearly showed that at both concentrations, and in defect of ligand (1:1:1 Nd:Dy:L), 50% of Ln ions were coordinated, hence saturating the ligand. Under stoichiometric conditions (1:1:2 Dy:Nd:L, hence 1:1 Ln:L) or in excess of ligand, all metal was in the complexed form and no preference or selectivity for either metal is observed. According to these data, there was a very slight preference in the complexation of Dy over Nd, but this appeared to occur only in the presence of an excess of metal. It is therefore evident that the different thermodynamic stability of the two metal complexes should not be the determining factor underlying their different solubility, which should be ascribed to the different nature of the molecular entities in solution.

## Conclusions

In this work, a series of functionalized heptadentate semirigid ligands belonging to the Trensal family were synthesized and tested as complexing agents for Nd^3+^ and Dy^3+^ ions in conventional organic solvents. This study sought to investigate the potential differences in coordination of the two metals and the properties of the corresponding metal complexes to promote their separation and recovery from end-of-life Nd(Dy)FeB permanent magnets. As we know well, the most critical step in the recovery of the metal components from this kind of scrap is represented by the separation of lanthanides. Indeed, their similar chemical properties and reactivity pose crucial challenges and increase the costs of the purification procedures. In this respect, this work contributes to the study centered on the coordination chemistry of these metals as a key point in identifying simple strategies for their separation. Specifically, on the basis of the extensive and systematic X-ray diffraction characterization of 13 new Trensal^R^-based metal complexes, six for Dy and seven for Nd, as well as the inspection of the CCDC database, it was evident that the heavier and smaller Dy preferred lower cn values such as 7 and 8 with respect to the lighter and larger Nd, which privileged cn values of 8 and 9 instead (see Figure S48). The semirigid ligands of the Trensal family allowed for the sequestration of the lanthanide ions, which appeared to be enough for the Dy requirements, because all of the reported structures exhibited the same structural motif. On the contrary, Nd necessitated additional ancillary ligands in the coordination sphere (solvent molecules). Overall, this apparently small structural difference between the Nd and Dy complexes led to a significant solubility difference between complexes [Nd(Trensal^*p*-OMe^)(H_2_O)] and [Dy(Trensal^*p*-OMe^)] in acetone. The separation capabilities achieved with the use of the Trensal^*p*-OMe^ ligand in a one-pot reaction and very mild conditions are good and confirmed the effectiveness previously demonstrated on similar systems.^53^ Optimization steps are nevertheless required to improve the metal separations for a potential scale-up process. On the contrary, our results may open the possibility of applying various ligand classes, also designed with the aim of limiting the cost and environmental impact as much as possible, to implement separation techniques that use green non-aqueous media that can provide refined REEs for high-value applications.

## Experimental Section

### Materials and Methods

All chemicals were purchased from Merck and Alfa Aesar and were used without further purification. Anhydrous solvents were dried and stored over molecular sieves (3 Å). NMR experiments were performed on a Brüker Avance 400 MHz instrument or a JEOL 600 MHz ECZ600R instrument at 298 K, and chemical shifts are reported in parts per million relative to tetramethylsilane. Infrared (IR) spectra were recorded with a Thermo Scientific Nicolet 5PC FT-IR-ATR (diamond) spectrometer in the range of 4000–400 cm^–1^. ESI-MS analyses were carried out by using a Waters Acquity Ultra Performance LC instrument with a Waters Acquity SQ Detector and an ESI interface. The mixtures were analyzed in negative ionization mode by direct perfusion in the ESI-MS interface; the injection flow rate was 0.2 mL/min. Elemental analyses (CHN) were performed on a Thermo Fischer Scientific FlashSmart CHNS analyzer.

### Collection of X-ray Data

Single-crystal data were collected with a Bruker D8 PhotonII area detector diffractometer (Mo Kα; λ = 0.71073 Å). Complete data sets were obtained by merging several series of exposure frames collected at 200 K. An absorption correction was applied with SADABS.^59^ The structures were determined with ShelxT^60^ and refined on *F*^2^ with full-matrix least squares (ShelxL^61^), using the Olex2 software package.^62^ Non-hydrogen atoms were refined with anisotropic thermal parameters for all compounds except **13**, which, apart from the metal, was refined with isotropic thermal parameters due to the poor quality of the data. In **11**·DMF, the coordinated solvent molecule was disordered over two sites. In **8**·ACN, the solvent laid on a 3-fold symmetry axis and was refined with an overall occupancy of 0.5. In **4**·3DMF, one of the solvent molecules was found to be disordered over two sites. In the structure of the complexes, the hydrogen atoms were placed at their calculated positions, whereas for Trensal^*p*-I^ and Trensal^*p*-OMe^, the hydrogen atoms of the OH groups were found by the residual electron density map and refined. The crystal structures of **1**(63) and **8**·ACN^64^ were previously reported and are mentioned here for comparison.

### UV–Vis Spectroscopy and Treatment of Data

The UV–vis spectra were recorded on a Photodiode Array Lambda 465 spectrophotometer provided with a Peltier thermostat, using 1 cm path length quartz cuvettes. Spectra of the ligand/metal systems were recorded in the spectral range of 250–500 nm. Titrations of the ligand with Nd or Dy in acetone or acetonitrile were carried out by adding aliquots (∼15 μL) of a 2 × 10^–3^ M solution of the metal in a 6.3 × 10^–5^ M solution of the ligand (initial volume of 2.6 mL, 1:3 ligand:triethylamine, 1:0–1.1 L:Ln). The calculations of the logarithms of the formation constants of the Ln/L species were carried out using HypSpec2014 software.^65^

### ICP-AES Analysis

The metal content of the sample was determined via inductively coupled plasma atomic emission spectroscopy (ICP-AES). From 5 to 15 mg of the solid sample was suspended in 5 mL of 65% HNO_3_ and 1 mL of 30% H_2_O_2_ and then digested in a Milestone model MLS-1200 MEGA microwave (digestion sequence: 1 min at 250 W, 1 min at 0 W, 5 min at 250 W, 5 min at 400 W, 5 min at 650 W, and 5 min of cooling). The solutions were diluted to 50 mL with bidistilled water and analyzed using an emission spectrometer (JY 2501) with coupled plasma induction in a radial configuration HORIBA Jobin Yvon (Kyoto, Japan), ULTIMA2 model. Instrumental features were as follows: monochromator, model JY 2501; focal length, 1 m; resolution, 5 pm; nitrogen flow rate, 2 L/min; ICP source, nebulizer; Meinhard cyclonic spraying chamber; argon flow rate, 12 L/min; wavelength range, 160–785 nm; optical bench temperature, 32 °C. The wavelength used for quantitative analysis was chosen by examining the emission line with greater relative intensity, ensuring that there was no spectral interference with the argon emission lines. The acquisition parameters were as follows: wavelength for Fe, 238.204 nm; wavelength for Nd, 410.946 nm; wavelength for Dy, 394.469 nm; voltage, 580 V; gain, 100. The quantitative analysis was performed after the acquisition of a calibration line using standard solutions in 10% HNO_3_ to simulate the final acidity of the samples; the concentration range of the standards varied from 0.1 to 50 mg/L of Fe, Dy, and Nd. Data were acquired and processed using ICP JY version 5.2 (Jobin Yvon). Measurements were performed in triplicate, and syntheses performed in duplicate.

### Synthetic Procedures

#### General Procedure for the Synthesis of Ligands **L_1_–L_6_**

Salicylaldehydes (3 mmol) were dissolved in absolute ethanol (20 mL). Tris(2-aminoethyl)amine (1 mmol) was then added while the mixture was being stirred at room temperature. The resulting yellow solution was stirred and refluxed for 3 h. The solution was cooled to room temperature, which yielded a precipitate that was filtered off. The precipitate was washed with cold absolute ethanol and dried under vacuum. The preparations of ligands Trensal (**L**_**1**_),^66^ Trensal^*p*-OMe^ (**L**_**2**_),^67^ Trensal^*p*-Cl^ (**L**_**4**_),^68,69^ and Trensal^*o*-tBu^ (**L**_**6**_)^70^ were previously reported. We list here the chemical characterization of **L**_**1**_**–L**_**6**_ for the sake of completeness.

##### Trensal (**L**_**1**_)

Yellow solid, 86% yield. ^1^H NMR (600 MHz, DMSO-*d*_6_): δ 13.57 (s, 3H, OH), 8.23 (s, 3H, CH-N_imine_), 7.30 (dt, 3H, Ar), 6.93 (dd, *J* = 7.7 Hz, *J* = 1.7 Hz, 3H, Ar), 6.87 (dd, *J* = 8.3 Hz, *J* = 1.1 Hz, 3H, Ar), 6.76 (dt, *J* = 7.4 Hz, *J* = 1.1 Hz, 3H, Ar), 3.57 (t, *J* = 6.1 Hz, 6H, N_imine_-CH_2_), 2.80 (t, *J* = 6.1 Hz, 6H, N-CH_2_). ^13^C NMR (101 MHz, DMSO-*d*_6_): δ 166.65, 161.38, 132.59, 131.97, 119.01, 118.73, 116.98, 57.40, 55.41. ESI-MS (MeOH): *m*/*z* 457.16 [**L**_**1**_]^−^. Anal. Calcd for C_27_H_30_N_4_O_3_: C, 70.72; H, 6.59; N, 12.21. Found: C, 70.7; H, 6.7; N, 12.3.

##### Trensal^*p*-OMe^ (**L**_**2**_)

Yellow solid, 81% yield. ^1^H NMR (400 MHz, DMSO-*d*_6_): δ 12.99 (s, 3H, OH), 8.28 (s, 3H, CH-N_imine_), 6.92 (dd, *J* = 8.9 Hz, *J* = 3.1 Hz, 3H, Ar), 6.80 (d, *J* = 8.9 Hz, 3H, Ar), 6.66 (d, *J* = 3.1 Hz, 3H, Ar), 3.63 (t, *J* = 6, Hz, 6H, N_imine_-CH_2_), 2.86 (t, *J* = 6.0 Hz, 6H, N-CH_2_). ^13^C NMR (101 MHz, DMSO-*d*_6_): δ 166.86, 153.18, 148.68, 123.64, 118.41, 117.57, 115.02, 56.63, 55.33, 56.15. ESI-MS (MeOH): *m*/*z* 547.61 [**L**_**2**_]^−^. Anal. Calcd for C_30_H_36_N_4_O_6_: C, 65.67; H, 6.61; N, 10.21. Found: C, 65.77; H, 6.75; N, 10.23.

##### Trensal^*o*-Cl^ (**L**_**3**_)

Brown solid, 52% yield. ^1^H NMR (400 MHz, DMSO-*d*_6_): δ 14.59 (s, 3H, OH), 8.36 (s, 3H, CH-N_imine_), 7,41 (dd, *J* = 7.7 Hz, *J* = 1.7 Hz, 3H, Ar), 7.01 (dd, *J* = 7.9 Hz, *J* = 1.7 Hz, 3H, Ar), 6.50 (t, *J* = 7.7 Hz, 3H, Ar), 3.67 (t, *J* = 5.8 Hz, 6H, N_imine_-CH_2_), 2.88 (t, *J* = 5.8 Hz, 6H, N-CH_2_). ^13^C NMR (101 MHz, DMSO-*d*_6_): δ 166.73, 163.13, 133.58, 131.80, 122.90, 117.83, 115.97, 54.52, 53.46. ESI-MS (MeOH): *m*/*z* 561.36 [**L**_**3**_]^−^. Anal. Calcd for C_27_H_27_N_4_O_3_Cl_3_: C, 57.71; H, 4.84; N, 9.97. Found: C, 55.48; H, 5.07; N, 8.74.

##### Trensal^*p*-Cl^ (**L**_**4**_)

Yellow solid, 78% yield. ^1^H NMR (400 MHz, DMSO-*d*_6_): δ 13.80 (s, 3H, OH), 8.21 (s, 3H, CH-N_imine_), 7.28 (dd, *J* = 8.8 Hz, *J* = 2.7 Hz, 3H, Ar), 6.93 (d, *J* = 2.7 Hz, 3H, Ar), 6.85 (d, *J* = 8.8 Hz, 3H, Ar), 3.60 (t, *J* = 5.7 Hz, 6H, N_imine_-CH_2_), 2.84 (t, *J* = 5.7 Hz, 6H, N-CH_2_). ^13^C NMR (101 MHz, DMSO-*d*_6_): δ 165.51, 160.66, 132.49, 130.66, 122.02, 119.52, 119.13, 56.73, 54.91. ESI-MS (MeOH): *m*/*z* 561.05 [**L**_**4**_]^−^. Anal. Calcd for C_27_H_27_N_4_O_3_Cl_3_: C, 57.71; H, 4.84; N, 9.97. Found: C, 57.98; H, 4.93; N, 9.94.

##### Trensal^*p*-I^ (**L**_**5**_)

Yellow solid, 78% yield. ^1^H NMR (400 MHz, DMSO-*d*_6_): δ 13.72 (s, 3H, OH), 8.32 (s, 3H, CH-N_imine_), 7.53 (dd, *J* = 8.7 Hz, *J* = 2.3 Hz, 3H, Ar), 7.46 (d, *J* = 2.3 Hz, 3H, Ar), 6.67 (d, *J* = 8.7 Hz, 3H, Ar), 3.63 (t, *J* = 5.9 Hz, 6H, N_imine_-CH_2_), 2.86 (t, *J* = 6.0 Hz, 6H, N-CH_2_). ^13^C NMR (101 MHz, DMSO-*d*_6_): δ 165.55, 161.89, 140.88, 139.70, 120.97, 120.11, 79.65, 56.58, 54.97. ESI-MS (MeOH): *m*/*z* 835.86 [**L_5_**]^−^. Anal. Calcd for C_27_H_27_N_4_O_3_I_3_: C, 38.77; H, 3.25; N, 6.69. Found: C, 39.19; H, 3.34; N, 6.77.

##### Trensal^*o*-tBu^ (**L**_**6**_)

Yellow solid, 74% yield. ^1^H NMR (400 MHz, DMSO-*d*_6_): δ 14.50 (s, 3H, OH), 8.15 (s, 3H, CH-N_imine_), 7.26 (dd, *J* = 7.7 Hz, *J* = 1.8 Hz, 3H, Ar), 6.67 (t, *J* = 7.6 Hz, 3H, Ar), 6.60 (dd, *J* = 7.7 Hz, *J* = 1.7 Hz, 3H, Ar), 3.59 (t, *J* = 5.9 Hz, 6H, N_imine_-CH_2_), 2.87 (t, *J* = 6.0 Hz, 6H, N-CH_2_), 1.34 (s, 27H, tBu). ^13^C NMR (101 MHz, DMSO-*d*_6_): δ 167.14, 160.90, 132.89, 130.34, 129.41, 118.58, 117.95, 57.42, 55.66, 34.83, 29.57. ESI-MS (MeOH): *m*/*z* 626 [**L**_**6**_]^−^. Anal. Calcd for C_39_H_54_N_4_O_3_: C, 74.71; H, 8.68; N, 8.94. Found: C, 74.83; H, 8.83; N, 9.00.

#### General Procedure for the Synthesis of Complexes

Ln(NO_3_)_3_·6H_2_O (1 mmol) dissolved in 5 mL of acetone or acetonitrile (**1**, **7**, and **8**) was added to a solution of Trensal^R^ ligand (1 mmol) and triethylamine (3 mmol) in the same solvent (10 mL). The resulting mixture was stirred and refluxed for 1 h. The precipitate obtained was filtered off, washed, and dried under vacuum.

##### [Dy(Trensal)]

Pale-yellow solid, 67% yield. Anal. Calcd for DyC_27_H_24_N_4_O_3_: C, 52.47; H, 4.40; N, 9.07. Found: C, 52.26; H, 4.43; N, 9.07. The compound can be recrystallized in DMF yielding colorless crystals of [Dy(Trensal)] (**1**).

##### [Dy(Trensal^*o*-Cl^)]

Pale-yellow solid, 65% yield. Anal. Calcd for DyC_27_H_24_N_4_O_3_Cl_3_: C, 44.95; H, 3.35; N, 7.77. Found: C, 44.79; H, 3.44; N, 8.09. The compound can be recrystallized in DMF yielding colorless crystals of [Dy(Trensal^*o*-Cl^)] (**2**).

##### [Dy(Trensal^*o*-tBu^)]

Pale-yellow solid, 58% yield. Anal. Calcd for DyC_39_H_51_N_4_O_3_: C, 59.57; H, 6.54; N, 7.12. Found: C, 59.73; H, 6.86; N, 6.90. Colorless crystals of [Dy(Trensal^*o*-tBu^)]·acetone (**3**·acetone) were obtained by cooling the acetone solution of the compound to room temperature.

##### [Dy(Trensal^*p*-I^)]

Pale-yellow solid, 67.8% yield. Anal. Calcd for DyC_27_H_24_N_4_O_3_I_3_: C, 32.57; H, 2.43; N, 5.63. Found: C, 32.28; H, 2.38; N, 5.73. The compound can be recrystallized in DMF yielding colorless crystals of [Dy(Trensal^*p*-I^)]·3DMF (**4**·3DMF).

##### [Dy(Trensal^*p*-OMe^)]

Pale-yellow solid, 44% yield. Anal. Calcd for DyC_30_H_33_N_4_O_6_: C, 50.89; H, 4.70; N, 7.91. Found: C, 50.92; H, 4.67; N, 7.69. The compound can be recrystallized in DMF yielding colorless crystals of [Dy(Trensal^*p*-OMe^)] (**5**).

##### [Dy(Trensal^*p*-Cl^)]

Pale-yellow solid, 65% yield. Anal. Calcd for DyC_27_H_24_N_4_O_3_Cl_3_: C, 44.95; H, 3.35; N, 7.77. Found: C, 44.84; H, 3.35; N, 7.95. The compound can be recrystallized in DMF yielding colorless crystals of [Dy(Trensal^*p*-Cl^)] (**6**).

##### [Nd(Trensal)(H_2_O)]0.5ACN

Light-blue solid, 87% yield. Anal. Calcd for C_27_H_24_N_4_O_3_Nd(C_2_H_3_N)_0.5_H_2_O: C, 52.68; H, 4.81; N, 9.87. Found: C, 53.04; H, 4.61; N, 9.84. The compound can be recrystallized in DMF yielding colorless crystals of [Nd(Trensal)(H_2_O)] (**7**).

##### [Nd(Trensal)]

In a test tube, a solution of Nd(NO_3_)_3_·6H_2_O (0.057 mmol, 25 mg) in ACN was layered over a solution of Trensal (0.085 mmol, 39.22 mg) and triethylamine (0.25 mmol, 0.035 mL) in DMF. The reaction was carried out at 60 °C overnight while the mixture was being stirred, yielding purple crystals of [Nd(Trensal)]·ACN (**8**·ACN).

##### [Nd(Trensal^*p*-I^)(H_2_O)]

Light-blue solid, 61% yield. Anal. Calcd for NdC_27_H_24_N_4_O_3_I_3_H_2_O: C, 32.58; H, 2.63; N, 5.63. Found: C, 32.67; H, 2.95; N, 5.57. The compound can be recrystallized in DMF yielding light-blue crystals of [Nd(Trensal^*p*-I^)(DMF)(H_2_O)]·DMF (**9**·DMF).

##### [Nd(Trensal^*o*-tBu^)]

Green solid, 68% yield. Anal. Calcd for NdC_39_H_51_N_4_O_3_: C, 60.98; H, 6.69; N, 7.29. Found: C, 60.59; H, 6.80; N, 7.07. The compound can be recrystallized in DMF yielding colorless crystals of [Nd(Trensal^*o*-tBu^)]·DMF (**10**·DMF).

##### [Nd(Trensal^*o*-Cl^)]

Light-blue solid, 77% yield. Anal. Calcd for NdC_27_H_24_N_4_O_3_Cl_3_: C, 46.12; H, 3.44; N, 7.97. Found: C, 45.76; H, 3.44; N, 8.12. The compound can be recrystallized in DMF yielding light-blue crystals of [Nd(Trensal^*o*-Cl^)(DMF)]·DMF (**11**·DMF).

##### [Nd(Trensal^*p*-Cl^)(H_2_O)]

Light-blue solid, 73% yield. Anal. Calcd for NdC_27_H_24_N_4_O_3_Cl_3_H_2_O: C, 44.97; H, 3.63; N, 7.77. Found: C, 45.24; H, 4.03; N, 7.48. The compound can be recrystallized in DMF yielding light-blue crystals of [Nd(Trensal^*p*-Cl^)(DMF)(H_2_O)] (**12**).

##### [Nd(Trensal^*p*-OMe^)(H_2_O)]

Yellow solid, 42% yield. Anal. Calcd for NdC_30_H_33_N_4_O_6_H_2_O: C, 50.90; H, 4.98; N, 7.91. Found: C, 50.50; H, 4.94; N, 7.61. Yellow crystals of [Nd(Trensal^*p*-OMe^(H_2_O)] (**13**) were obtained by cooling the ACN solution of the compound to room temperature.

## Supporting Information Available

NMR and ESI-MS spectra of the ligands, UV–vis titration experiments, EDX, and single-crystal X-ray structural characterization (PDF)
A video with the time line of the precipitation of the complexes (AVI)

## Supplementary Material

ic2c02619_si_001.pdf

ic2c02619_si_002.avi

## Abbreviations

REE: rare-earth element
NdFeB: neodymium iron boron
Ln: lanthanide
Nd: neodymium
Dy: dysprosium
cn: coordination number
DMF: *N,N*-dimethylformamide
ACN: acetonitrile
