# Supplementary material for: Semirigid Ligands Enhance Different Coordination Behavior of Nd and Dy Relevant to Their Separation and Recovery in a Non-aqueous Environment
Source: Inorg Chem. 2022 Sep 30;61(40):16110–21. doi: 10.1021/acs.inorgchem.2c02619 (PMC9554911; doi:10.1021/acs.inorgchem.2c02619)
Supplement: Supplementary file 1 — ic2c02619_si_001.pdf [file ic2c02619_si_001.pdf]

SUPPORTING INFORMATION

Semi-rigid ligands enhance different coordination behavior of Nd and Dy relevant to their separation and recovery in non-aqueous environment

Alex Falco,[a] Martina Neri, [a] Matteo Melegari, [a] Laura Baraldi, [a] Giulia Bonfant, [a] Matteo Tegoni, [a] Angela Serpe, [b,c]* Luciano Marchiò [a]*

[a]*Department of Chemistry, Life Sciences and Environmental Sustainability, University of Parma, Parco Area delle Scienze 17/A, 43124 Parma, Italy.*

[b] *Department of Civil and Environmental Engineering and Architecture (DICAAR), and research unit of INSTM, University of Cagliari, Via Marengo 2, 09123 Cagliari, Italy.*

[c] *Environmental Geology and Geoengineering Institute of the National Research Council (IGAG-CNR), Piazza d'Armi, 09123 Cagliari, Italy.*

Corresponding author: luciano.marchio@unipr.it, serpe@unica.it

Table of contents

List of structurally characterized compounds S2
NMR spectra of the ligands S3
ESI-MS spectra of the ligands S6
Uv-Visible measurements S9
Nd/Dy Trensal^R complexes S13
EDX analysis S14
Theoretical partition coefficients of ligands S15
Single Crystal X-ray Structures S16
ICP-AES analysis S32
Crystal data and structural geometric parameters S34

List of X-ray structurally characterized compounds

[Dy(Trensal)] (**1**)

[Dy(Trensal$^{o\text{-}Cl}$)] (**2**)

[Dy(Trensal$^{o\text{-}tBu}$)]·Acetone (**3**·Acetone)

[Dy(Trensal$^{p\text{-}I}$)]·3DMF (**4**·3DMF)

[Dy(Trensal$^{p\text{-}OMe}$)] (**5**)

[Dy(Trensal$^{p\text{-}Cl}$)] (**6**)

Nd(Trensal)(H_2O)] (**7**)

[Nd(Trensal)]·ACN (**8**·ACN)

[Nd(Trensal$^{p\text{-}I}$)(DMF)(H_2O)]·DMF (**9**·DMF)

[Nd(Trensal$^{o\text{-}tBu}$)]·DMF (**10**·DMF)

[Nd(Trensal$^{o\text{-}Cl}$)(DMF)]·DMF (**11**·DMF)

[Nd(Trensal$^{p\text{-}Cl}$)(DMF)(H_2O)] (**12**)

[Nd(Trensal$^{p\text{-}OMe}$)(H_2O)] (**13**)

Trensal$^{p\text{-}I}$ (**L**$_{\mathbf{5}}$)

Trensal$^{p\text{-}OMe}$ (**L**$_{\mathbf{2}}$)

NMR spectra of the ligands

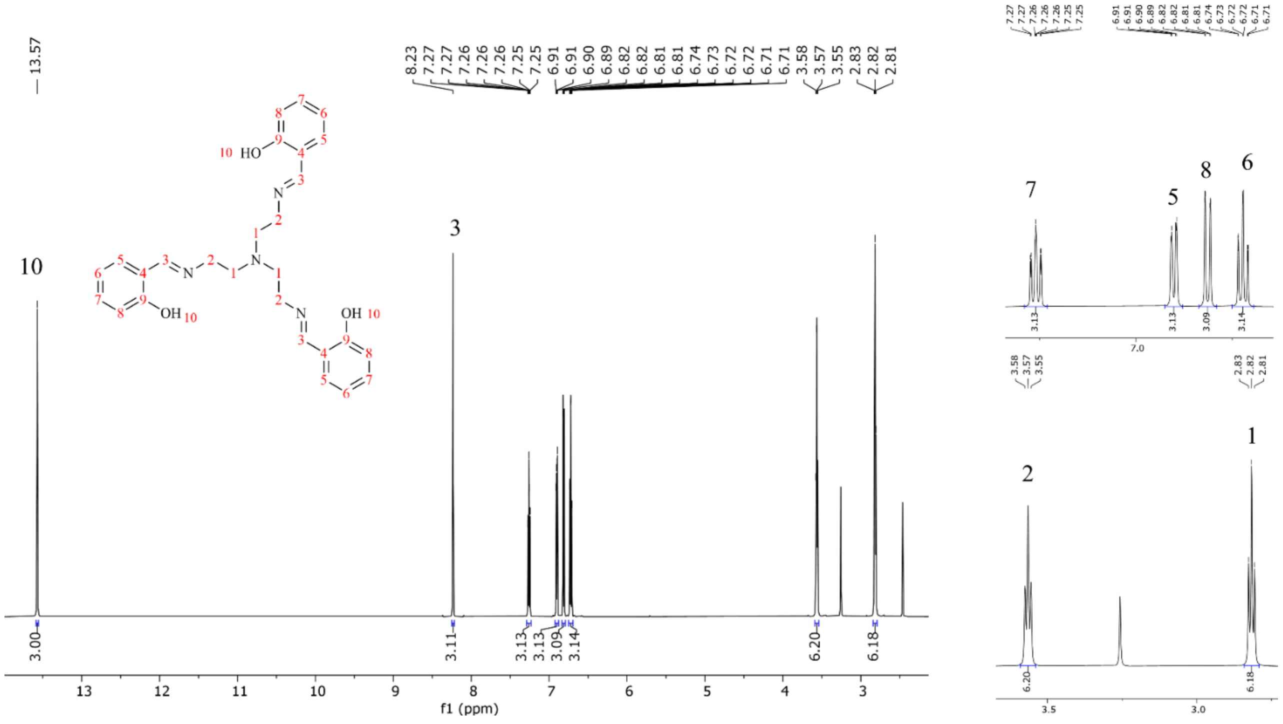

Figure S1. ^{1}H-NMR (600 MHz, DMSO) spectrum of L_1.

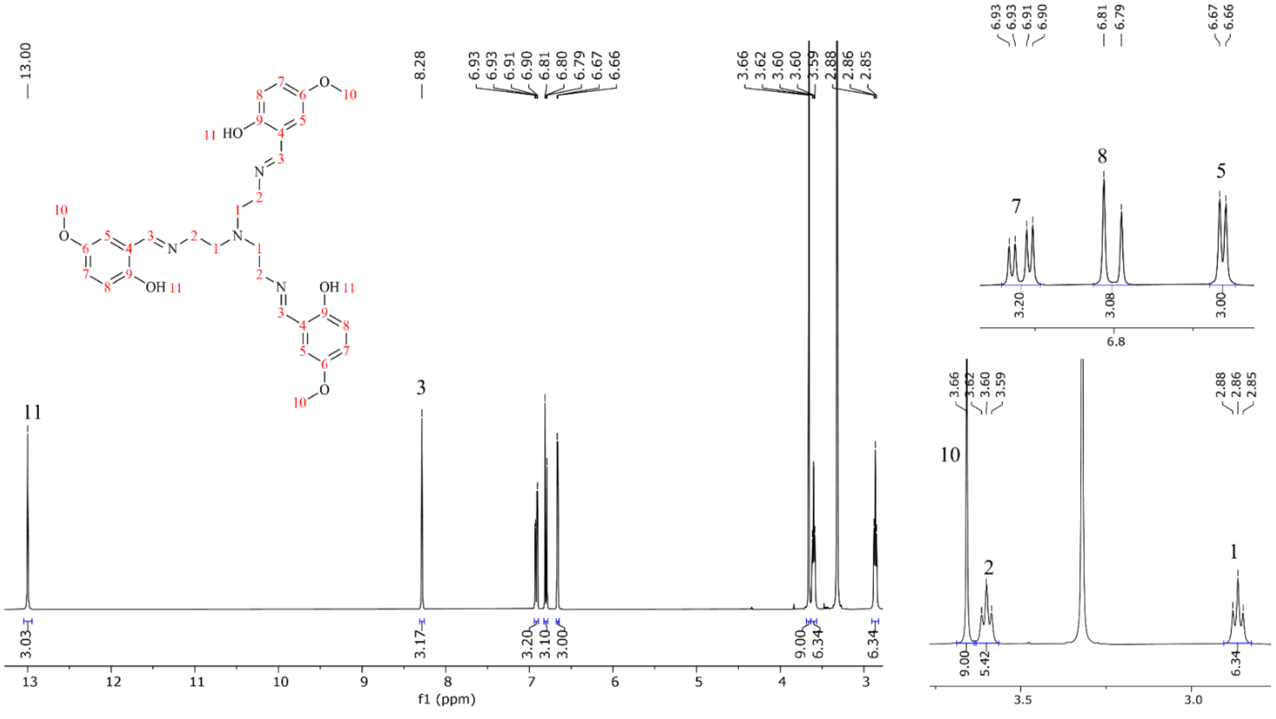

Figure S2. ^{1}H-NMR (400 MHz, DMSO) spectrum of L_2.

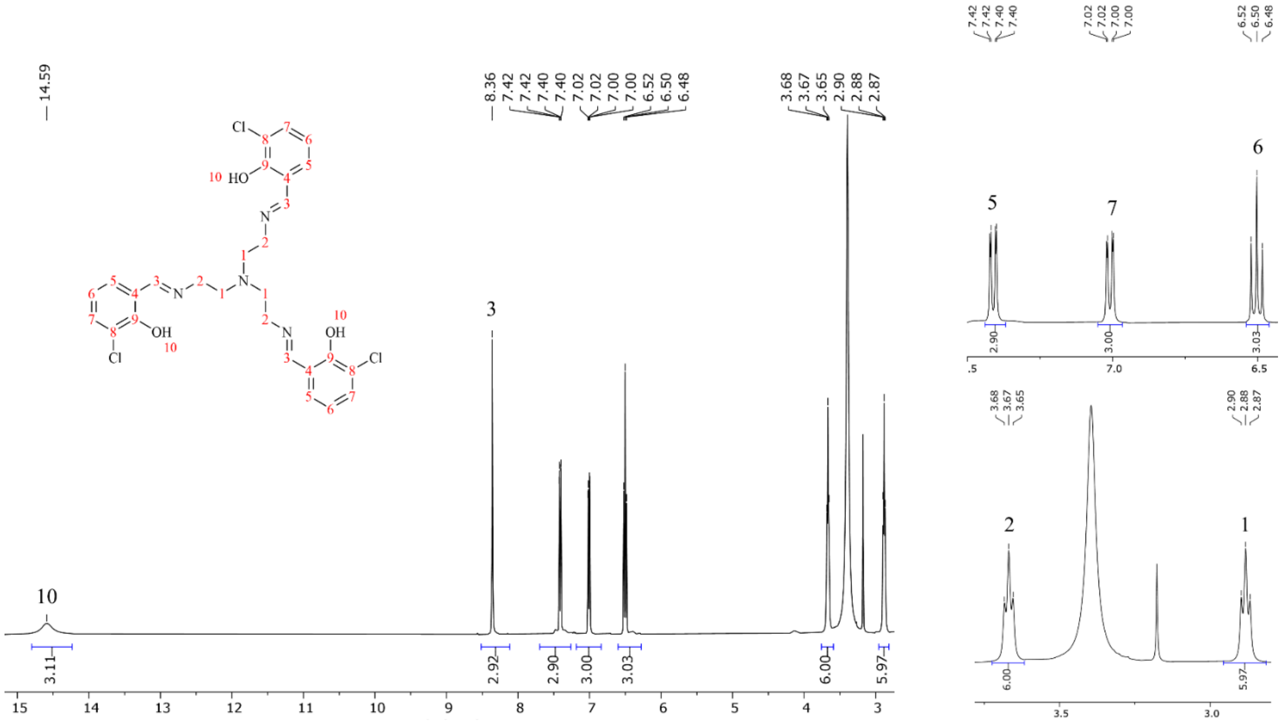

Figure S3. ^{1}H-NMR (400 MHz, DMSO) spectrum of L_3.

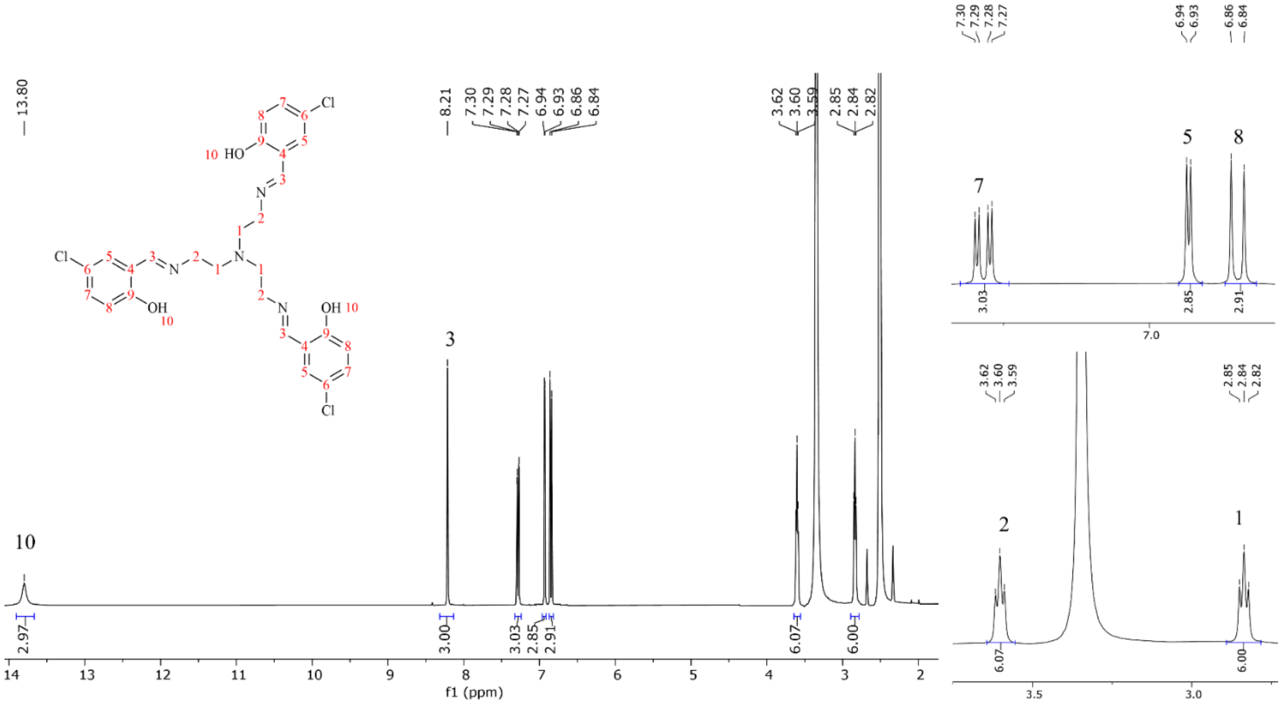

Figure S4. ^{1}H-NMR (400 MHz, DMSO) spectrum of L_4.

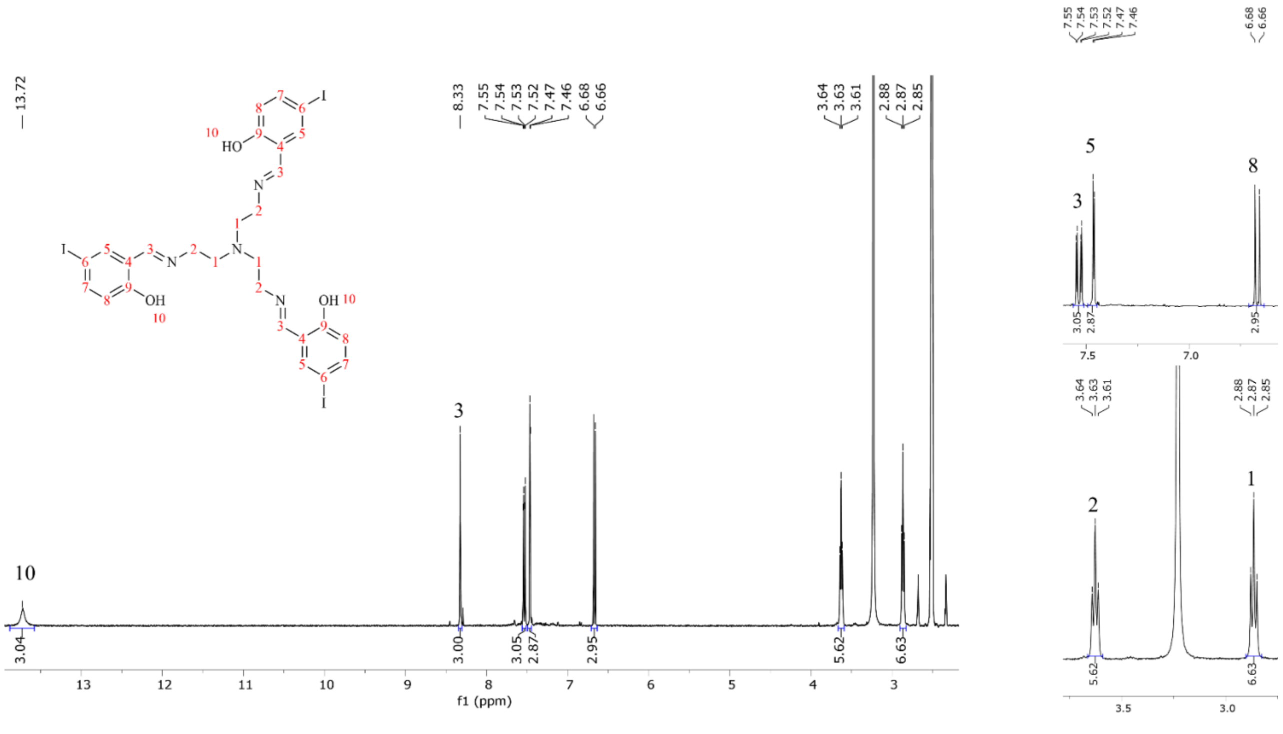

Figure S5. ^{1}H-NMR (400 MHz, DMSO) spectrum of L_5.

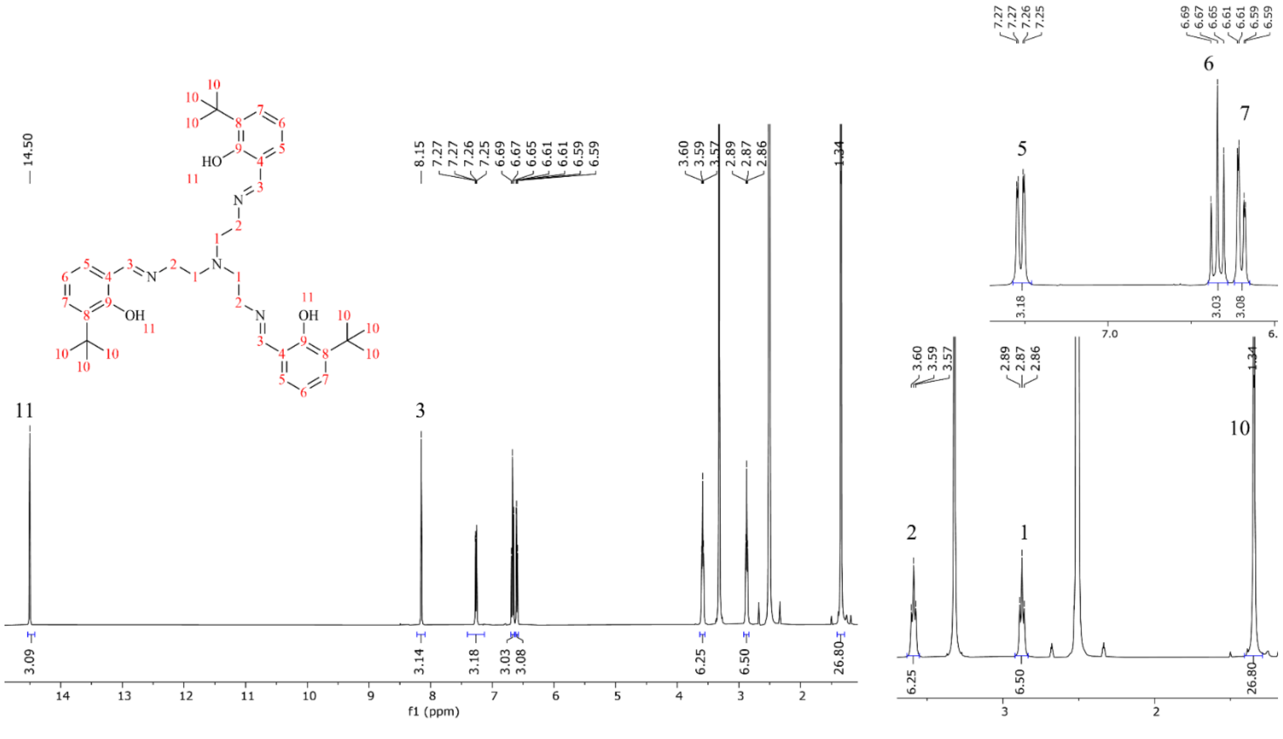

Figure S6. ^{1}H-NMR (400 MHz, DMSO) spectrum of L_6.

ESI-MS spectra of the ligands

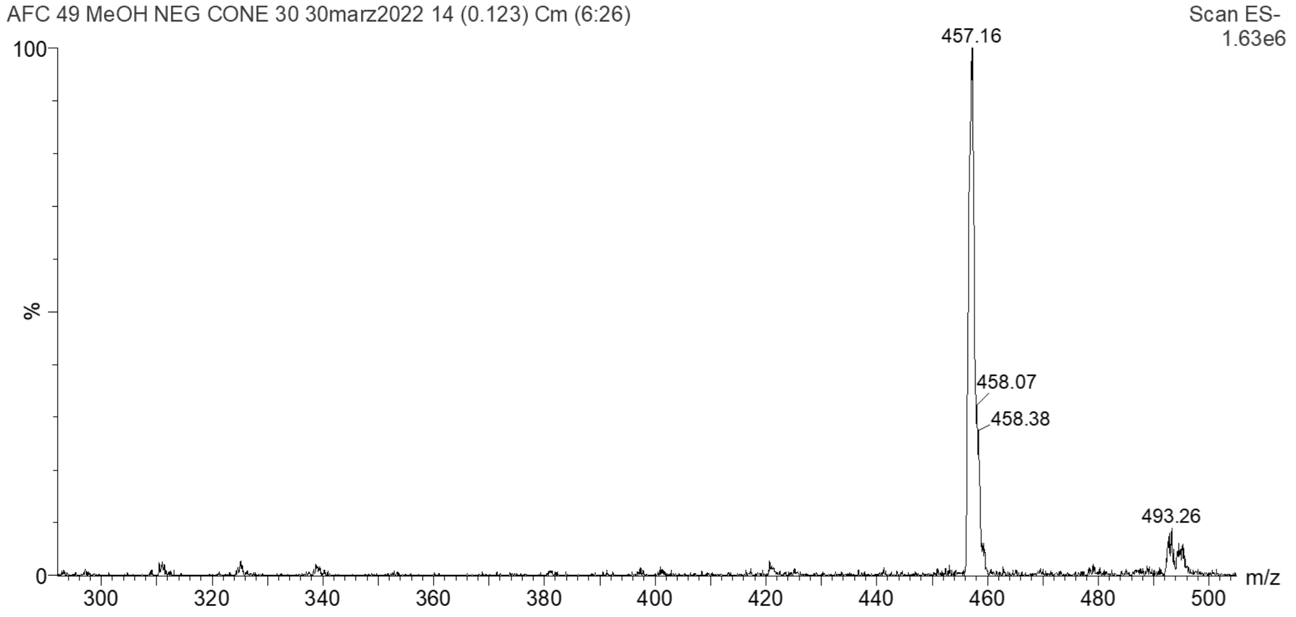

Figure S7. UPLC-ESI (-) mass spectrum of L_1 (CH_3OH); *m/z* $[L_1]^-$= 457.16.

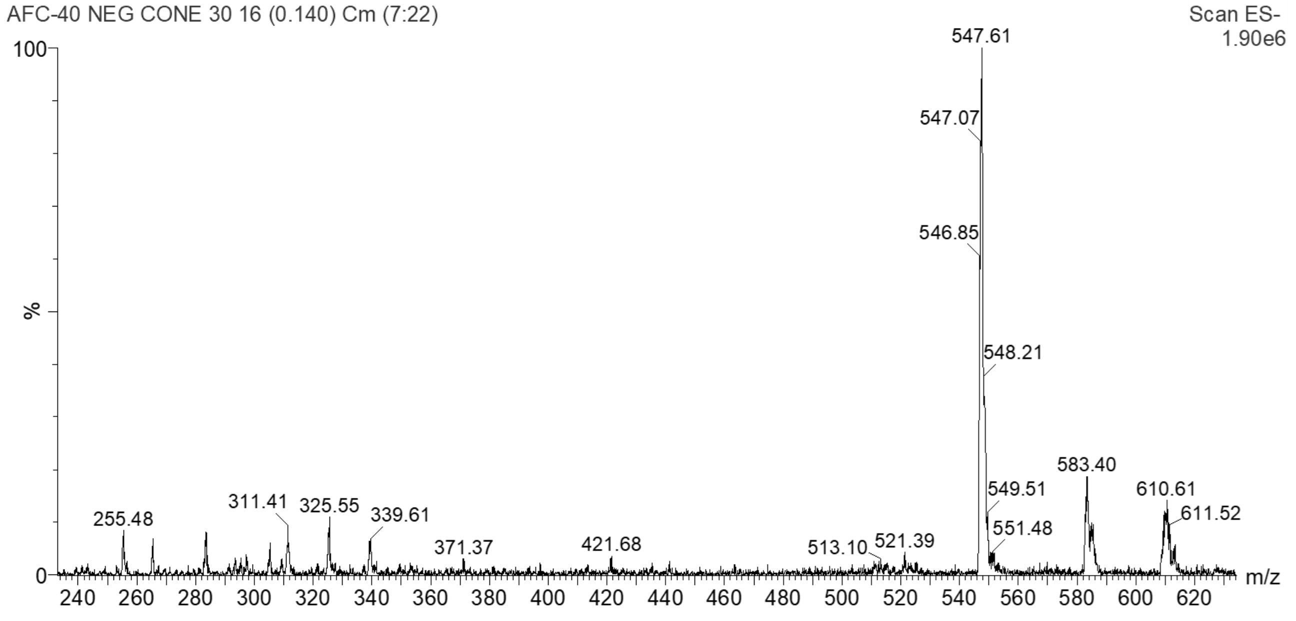

Figure S8. UPLC-ESI (-) mass spectrum of L_2 (CH_3OH); *m/z* $[L_2]^-$= 547.61.

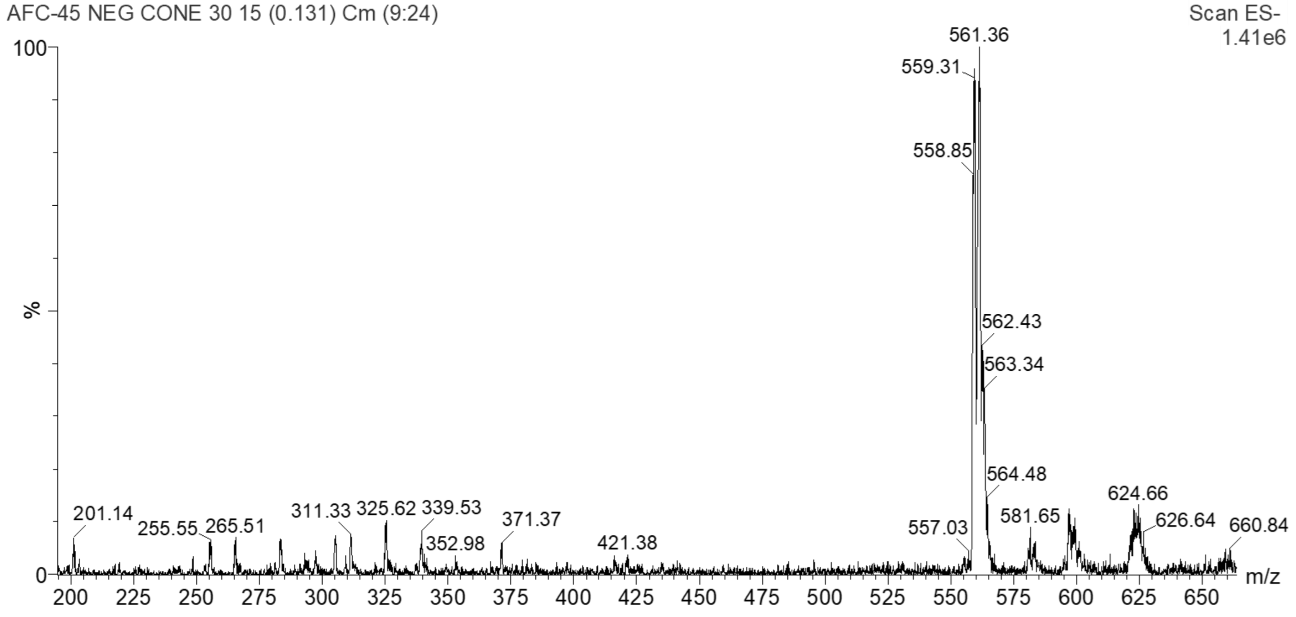

Figure S9. UPLC-ESI (-) mass spectrum of L_3 (CH_3OH); *m/z* $[L_3]^-$= 561.36.

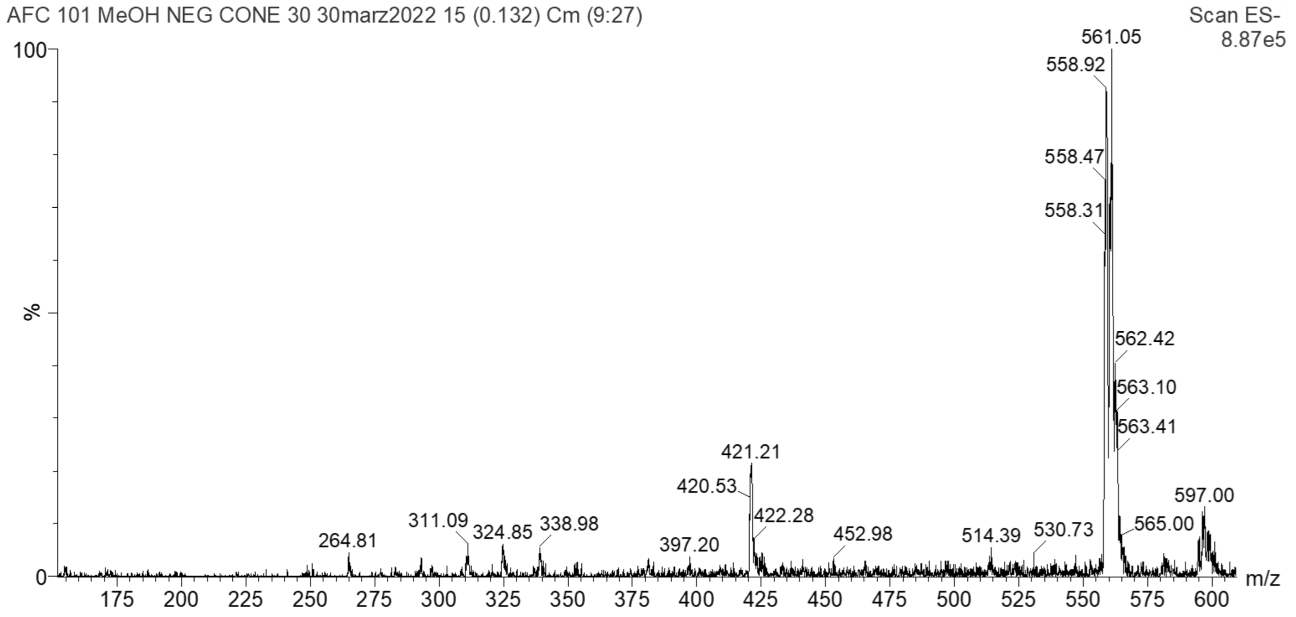

Figure S10. UPLC-ESI (-) mass spectrum of L_4 (CH_3OH); *m/z* $[L_4]^-$= 561.05.

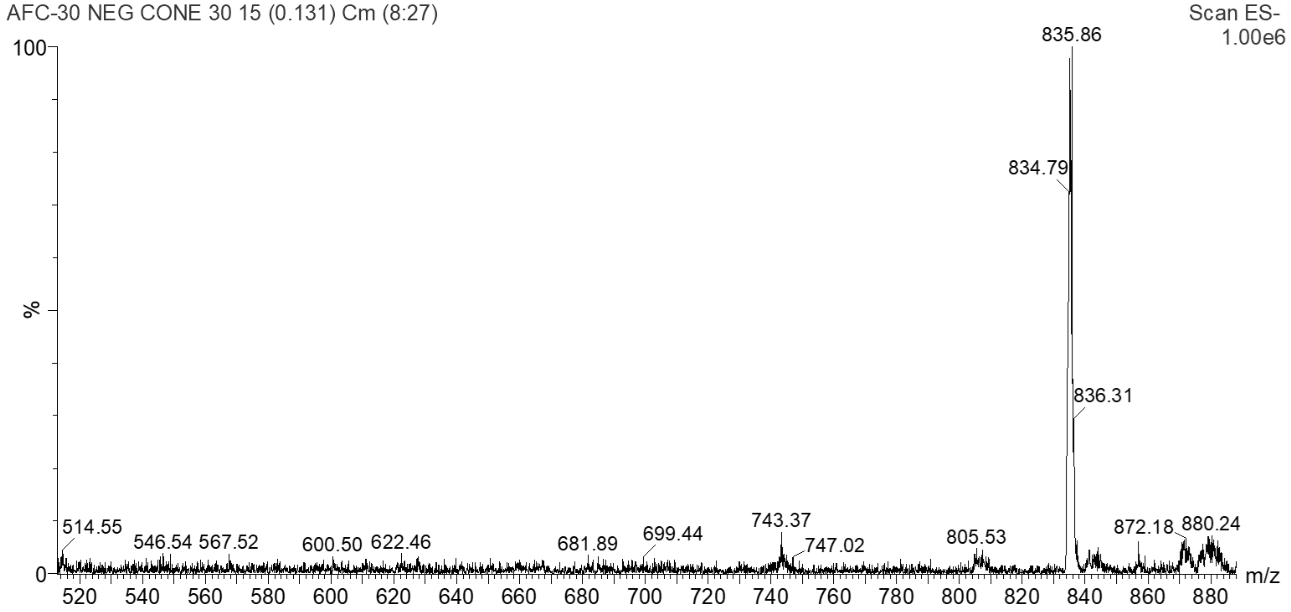

Figure S11. UPLC-ESI (-) mass spectrum of L_5 (CH_3OH); *m/z* $[L_5]^-$= 835.86.

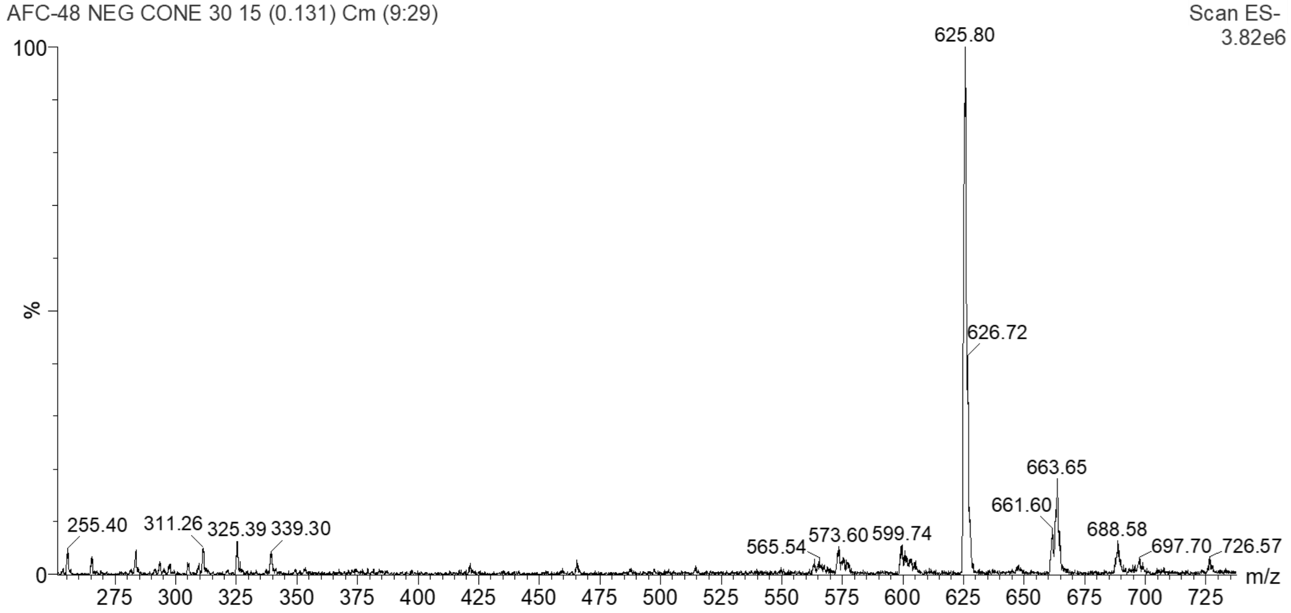

Figure S12. UPLC-ESI (-) mass spectrum of L_6 (CH_3OH); *m/z* $[L_6]^-$= 625.80.

Uv-Visible measurements

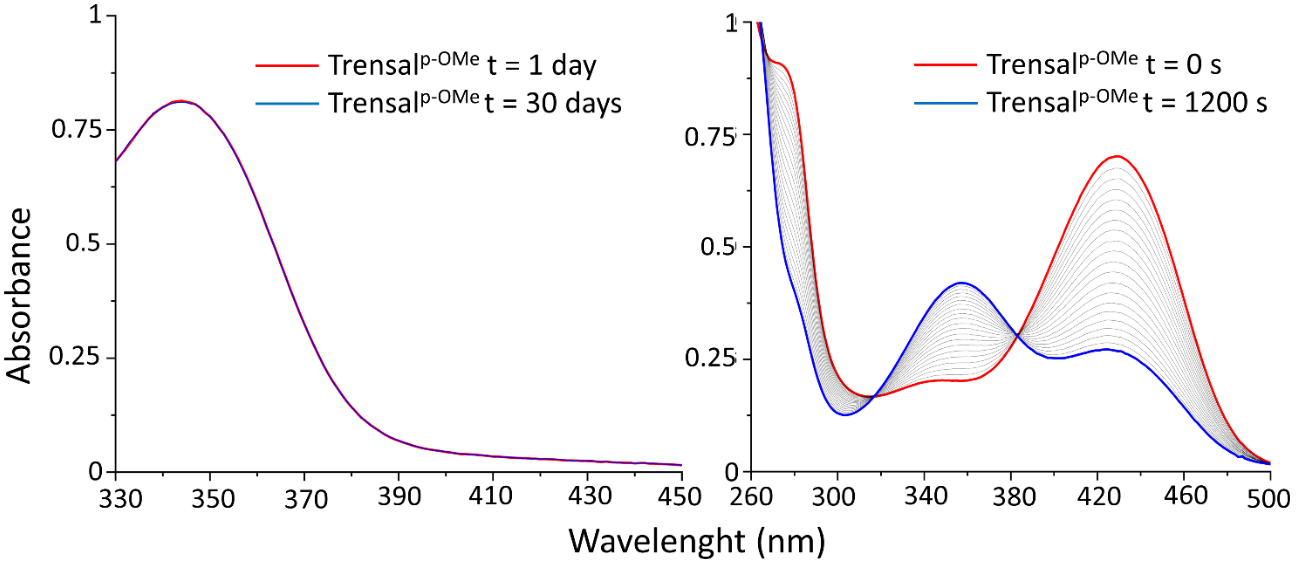

Figure S13. Study of the Trensal$^{p\text{-}OMe}$ stability over time. Uv-Vis spectra were collected under stirring at 25 °C in acetone (left) and in 50 mM aqueous HEPES buffer at pH 7 (right). UV-Visible spectra were collected every 60 s for 1200 s. The tris-imino functional groups of the Trensal-type ligands are the source of chemical instability in aqueous media. Indeed, the ligands are not stable in water and, as expected, they undergo hydrolysis in a very short time (less than 3 min.) at pH 7.

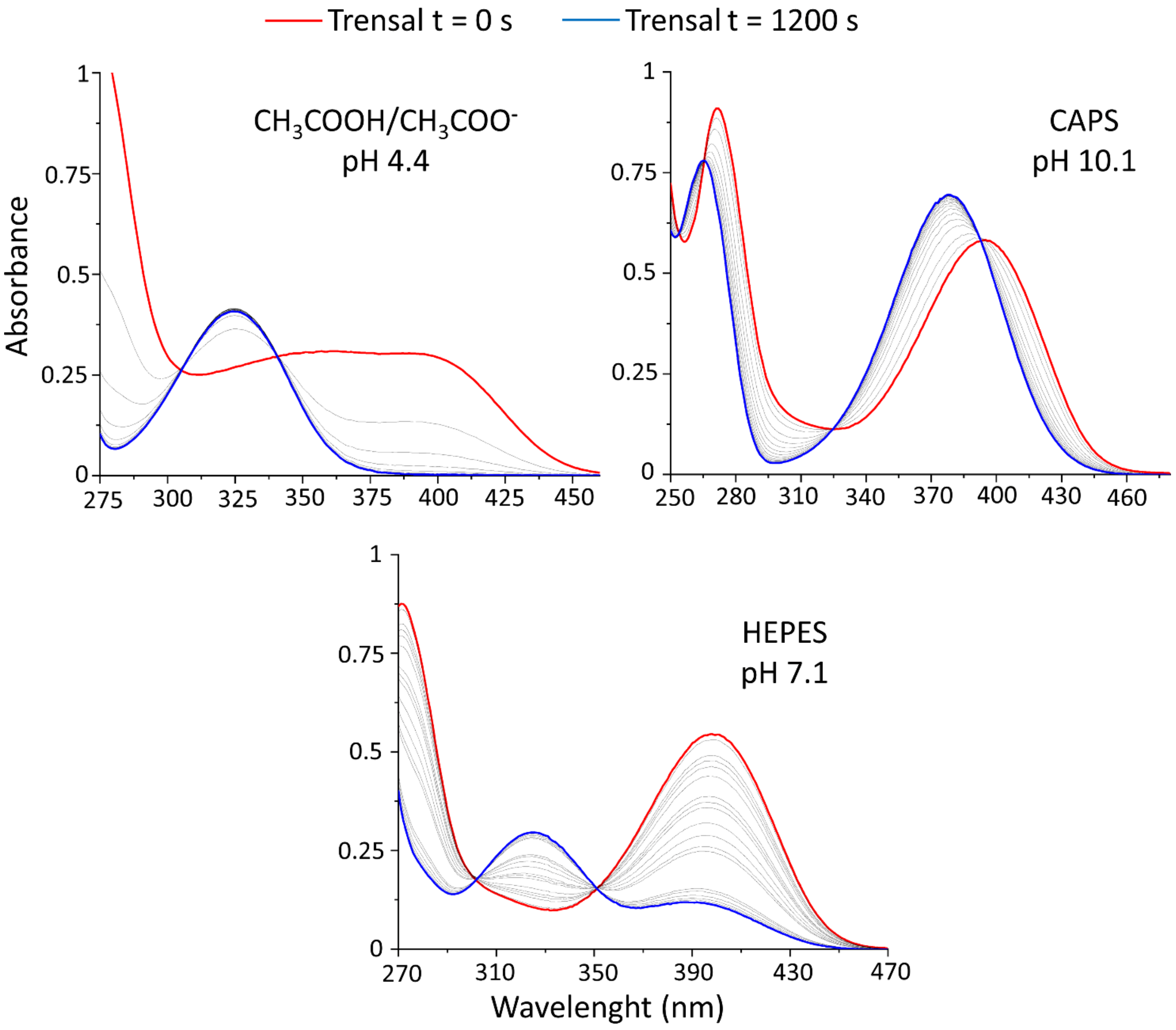

Figure S14. Study of the Trensal$^{p\text{-}OMe}$ stability over time. Uv-Vis spectra were collected under stirring at 25 °C in 50 mM aqueous acetate buffer at pH 4.4 (left), in 50 mM aqueous CAPS buffer at pH 10.1 (right) and in 50 mM aqueous HEPES buffer at pH 7.1 (central). UV-Visible spectra were collected every 60 s for 1200 s. The tris-imino functional groups of the Trensal-type ligands are the source of chemical instability in aqueous media. Indeed, the ligands are not stable in water and, as expected, they undergo hydrolysis in a very short time (less than 3 min.) at different pH values.

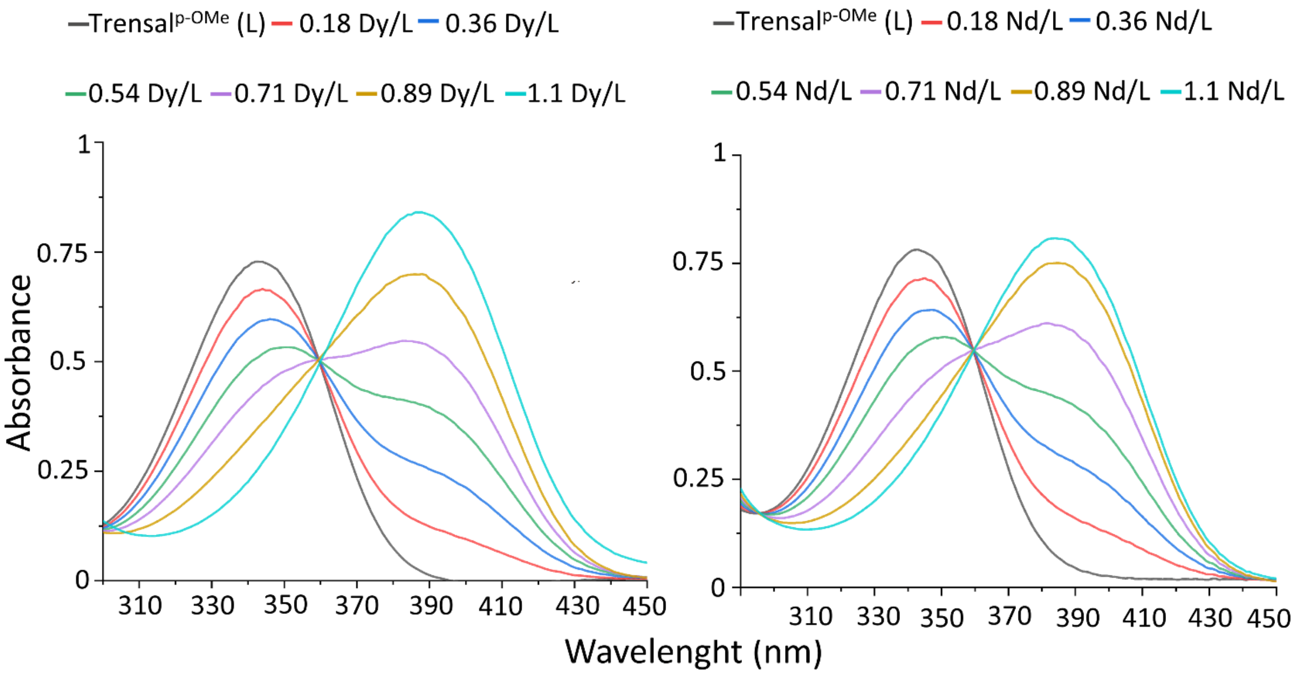

Figure S15. Trensal$^{p\text{-}OMe}$ titrations with $Dy(NO_3)_3{\cdot}6H_2O$ (left) and $Nd(NO_3)_3{\cdot}6H_2O$ (right). Spectra were collected under stirring at 25°C in acetonitrile. The Trensal$^{p\text{-}OMe}$ solution ($6.46{\cdot}10^{-5}$ M) was titrated by a proper aliquot of $Dy(NO_3)_3{\cdot}6H_2O$ ($2{\cdot}10^{-3}$ M) and $Nd(NO_3)_3{\cdot}6H_2O$ ($2{\cdot}10^{-3}$ M) stock solutions.

a)

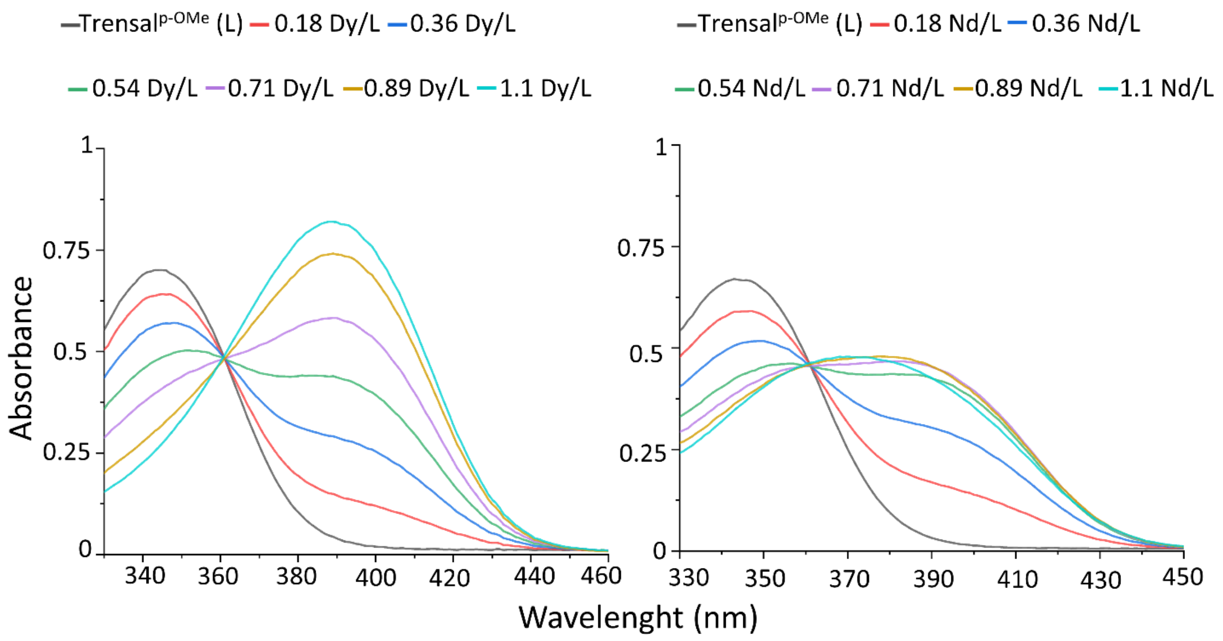

b)

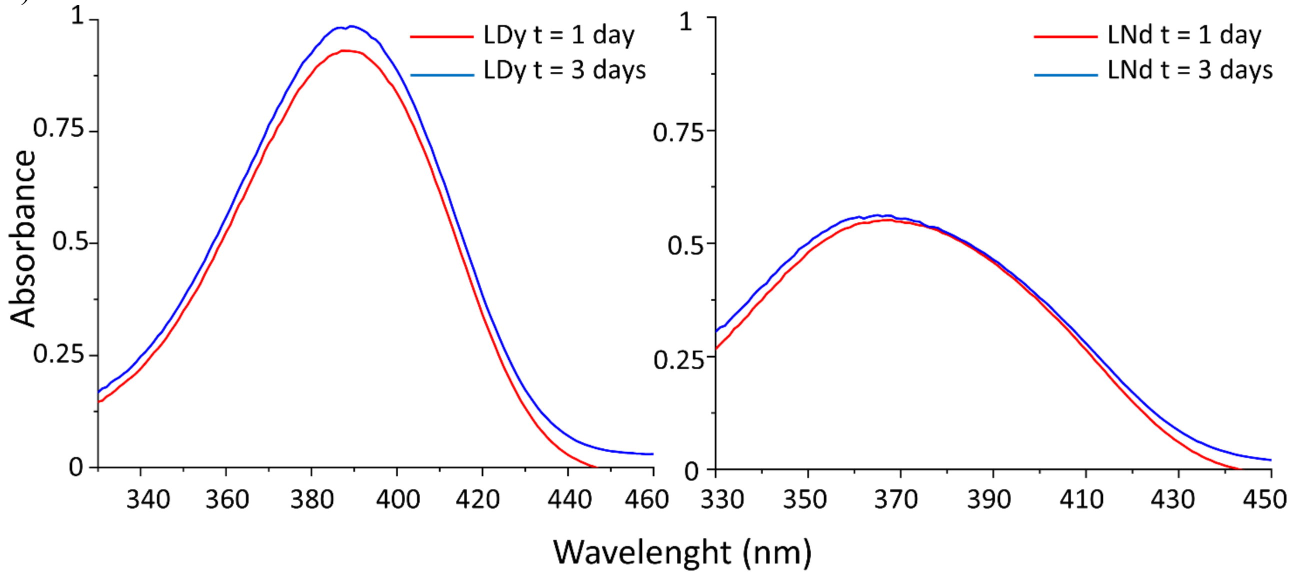

Figure S16. a) Trensal$^{p\text{-}OMe}$ titrations with $Dy(NO_3)_3{\cdot}6H_2O$ (left) and $Nd(NO_3)_3{\cdot}6H_2O$ (right). Spectra were collected under stirring at 25 °C in acetone. The Trensal$^{p\text{-}OMe}$ solution ($6.28{\cdot}10^{-5}$ M) was titrated by a proper aliquot of $Dy(NO_3)_3{\cdot}6H_2O$ ($2{\cdot}10^{-3}$ M) and $Nd(NO_3)_3{\cdot}6H_2O$ ($2{\cdot}10^{-3}$ M) stock solutions. b) Investigation of the of [Nd(Trensal$^{p\text{-}OMe}$)(H_2O)] and [Dy(Trensal$^{p\text{-}OMe}$)] stability in acetone over time. The UV-vis spectra were collected at 25 °C.

Nd/Dy Trensal^{R} complexes

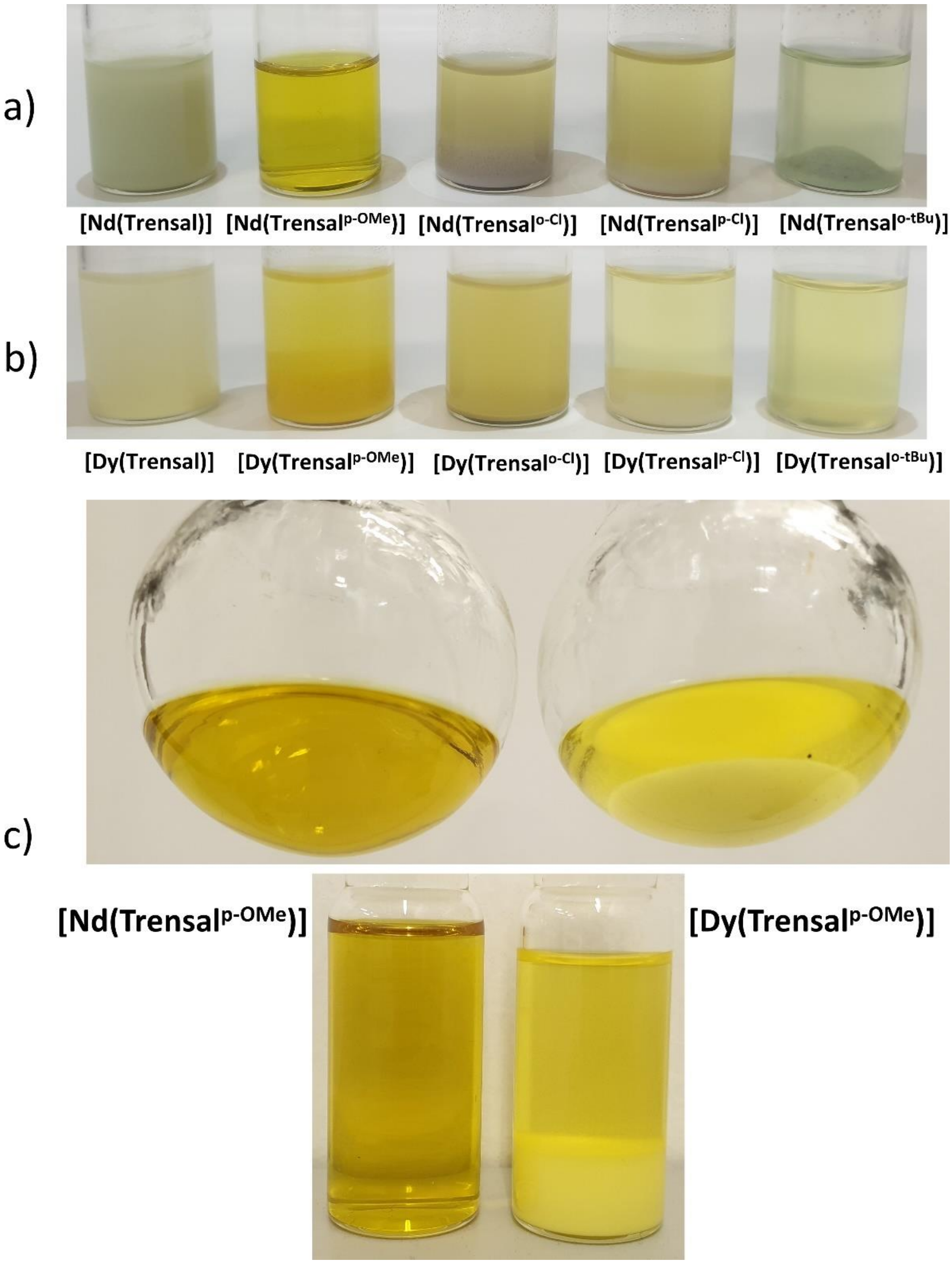

Figure S17. Images of Trensal^{R} complexes of Nd (a) and Dy (b) as prepared by mixing equimolar amount of ligand and metal; images of [Nd(Trensal$^{p\text{-}OMe}$)(H_2O)] (**13**) (c, left) and [Dy(Trensal$^{p\text{-}OMe}$)] (**5**) (c, right) synthesized as follows: $Ln(NO_3)_3{\cdot}6H_2O$ (1 eq) (Ln= Nd, Dy), Trensal$^{p\text{-}OMe}$ (1 eq), triethylamine (3 eq) in acetone (15 mL).

EDX analysis

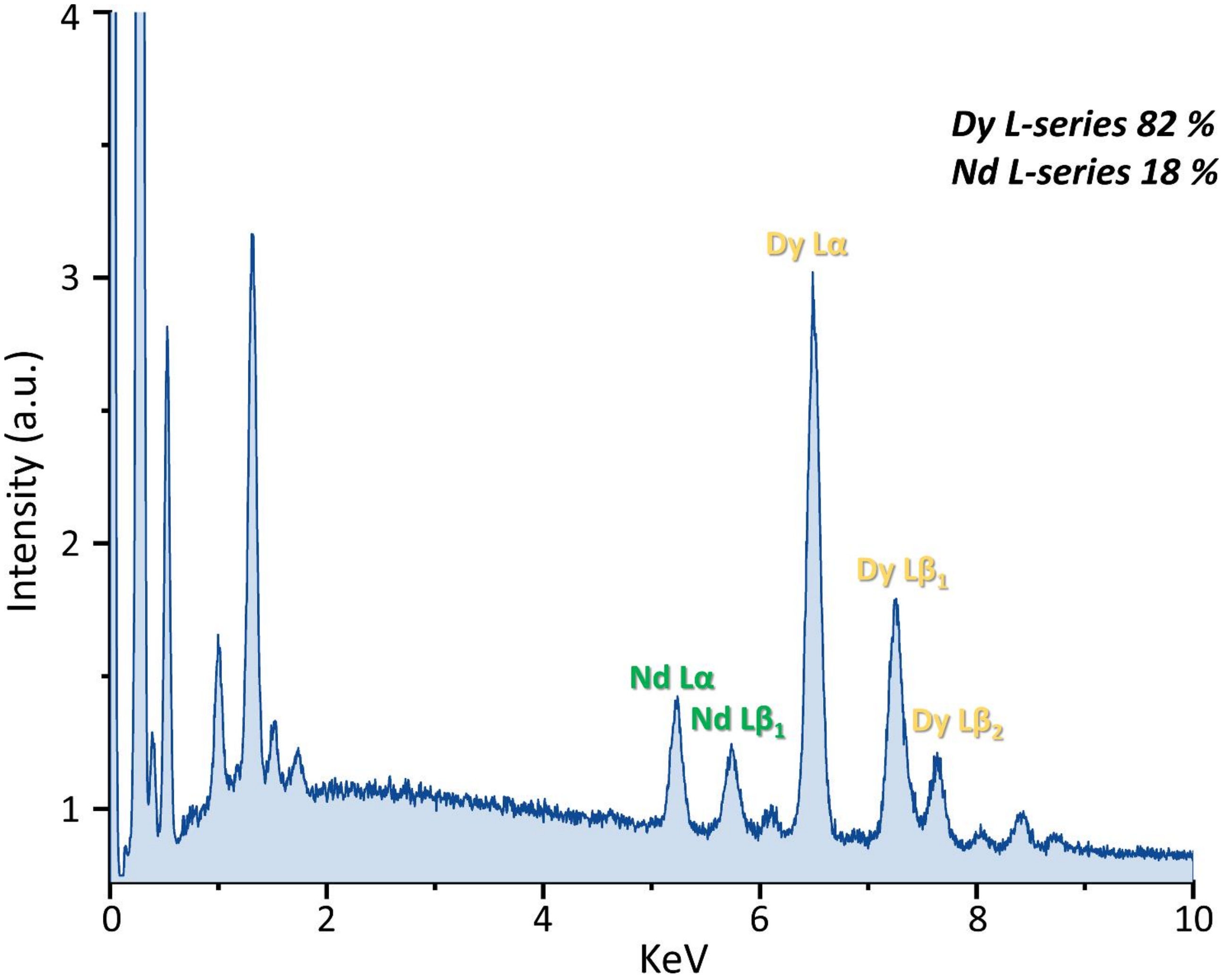

Figure S18. EDX and semi-quantitative analysis performed on the solid obtained by mixing Nd:Dy: Trensal$^{p\text{-}OMe}$ in the 1:1:3 ratio in acetone.

Theoretical partition coefficients of ligands

Table S1. The theoretical partition coefficients of the ligands are showed in increasing order of lipophilicity.

Ligand	logP
Trensal (L_1)	4.54
Trensal$^{p\text{-}OMe}$ (L_2)	4.64
Trensal$^{o\text{-}Cl}$ (L_3)	5.81
Trensal$^{p\text{-}Cl}$ (L_4)	6.51
Trensal$^{p\text{-}I}$ (L_5)	7.72
Trensal$^{o\text{-}tBu}$ (L_6)	8.64

Single Crystal X-ray Structures

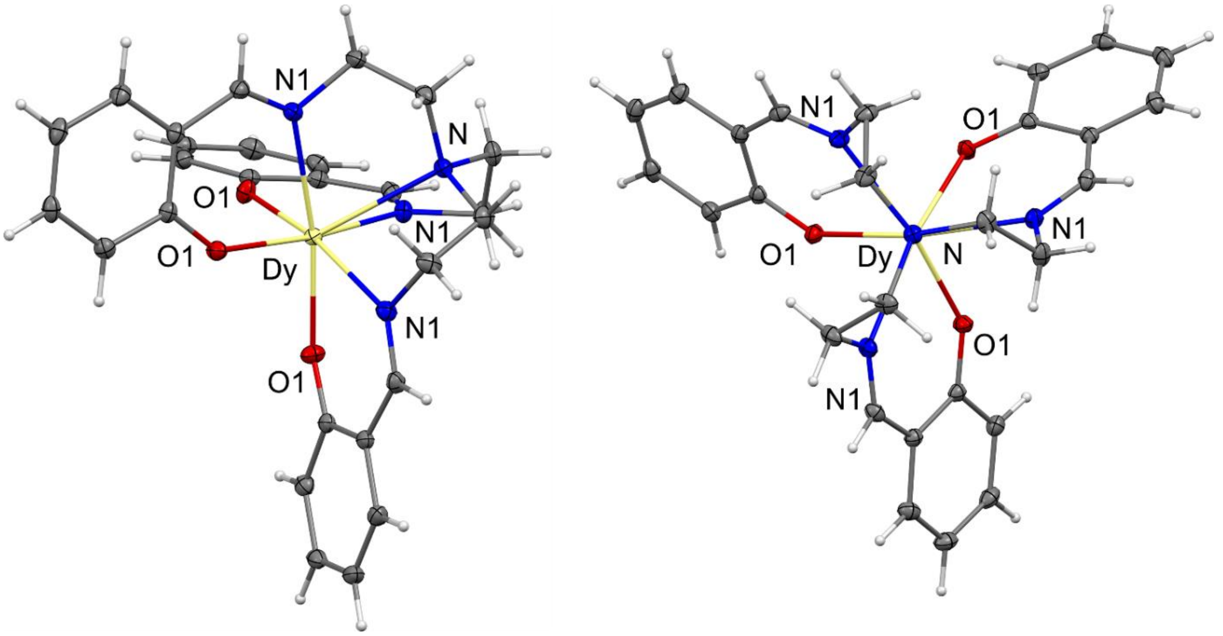

Figure S19. Representation of the structure of **1**. Thermal ellipsoids were depicted at the 30% probability level. Capped octahedron molecular geometry (left). View along the central axis N-Dy (right). Dy (pale-yellow), N (blue), O (red), C (gray) and H (light gray).

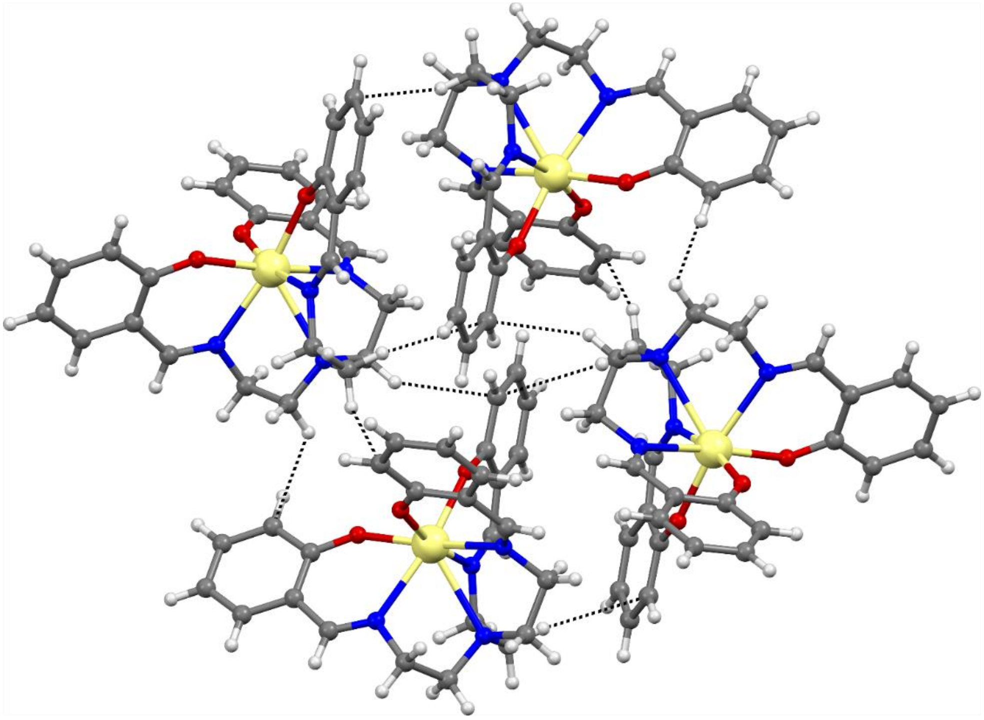

Figure S20. Portion of the crystal packing of **1**. C-H···π represent the main interaction (dashed bonds). Dy (pale-yellow), N (blue), O (red), C (gray) and H (light gray).

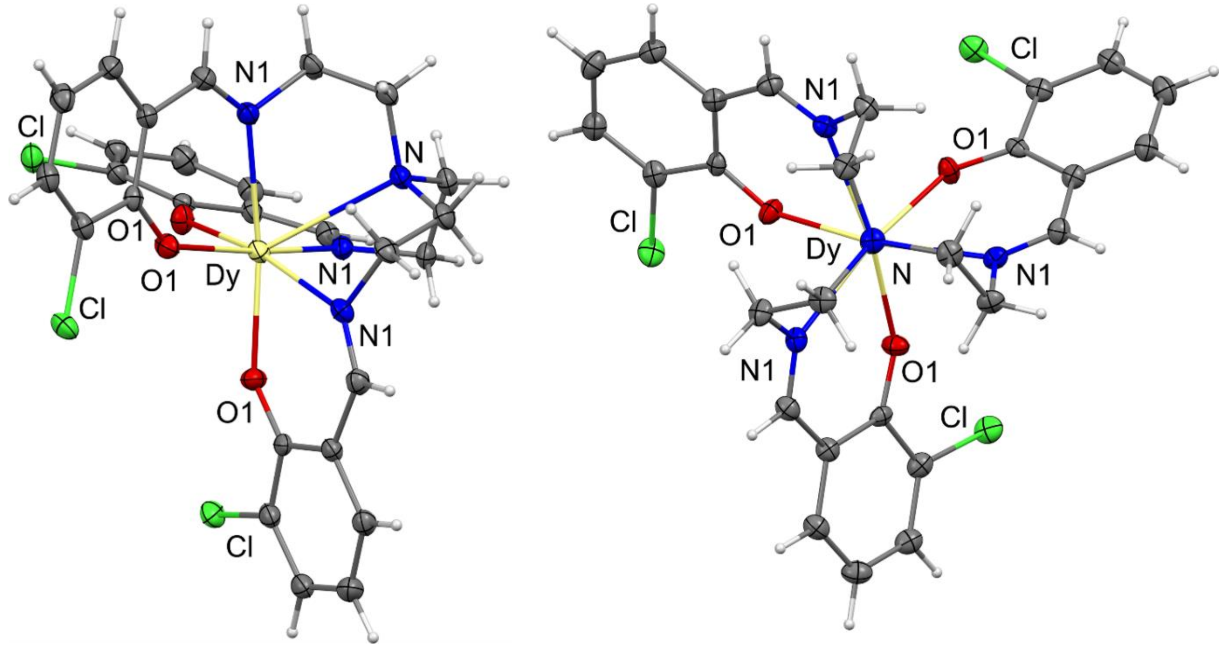

Figure S21. Representation of the structure of **2**. Thermal ellipsoids were depicted at the 30% probability level. Capped octahedron molecular geometry (left). View along the central axis N-Dy (right). Dy (pale-yellow), N (blue) O (red), Cl (green), C (gray) and H (light gray).

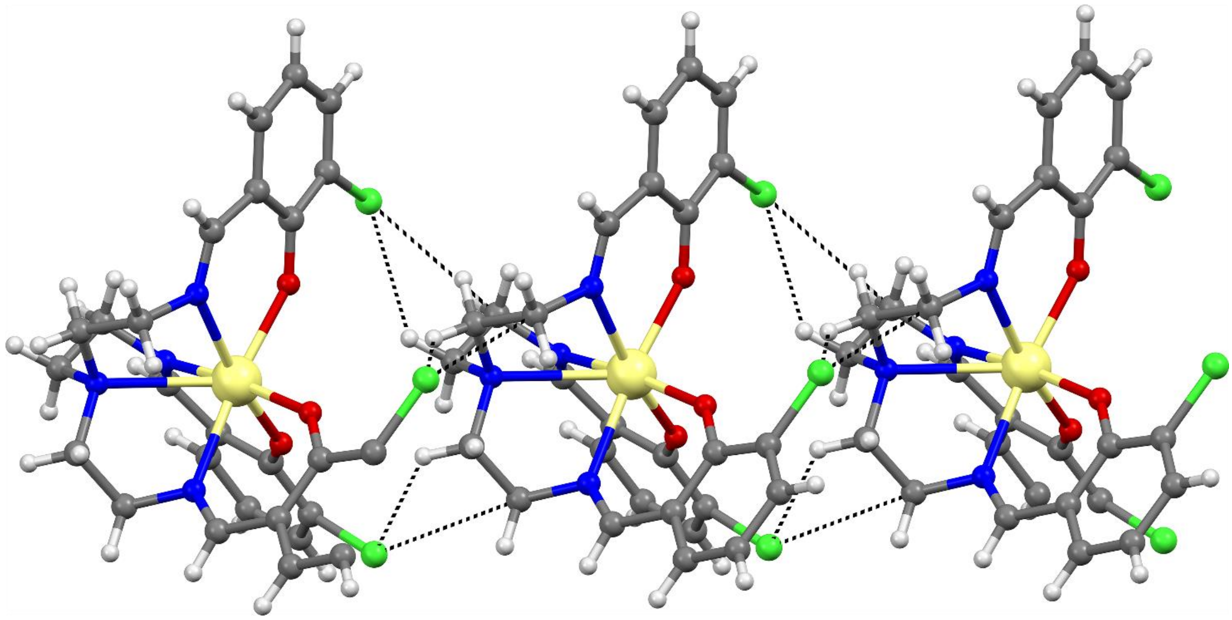

Figure S22. Portion of the crystal packing of **2**. C-H···Cl represent the main interaction (Dashed bonds). Dy (pale-yellow), N (blue), O (red), Cl (green), C (gray) and H (light gray).

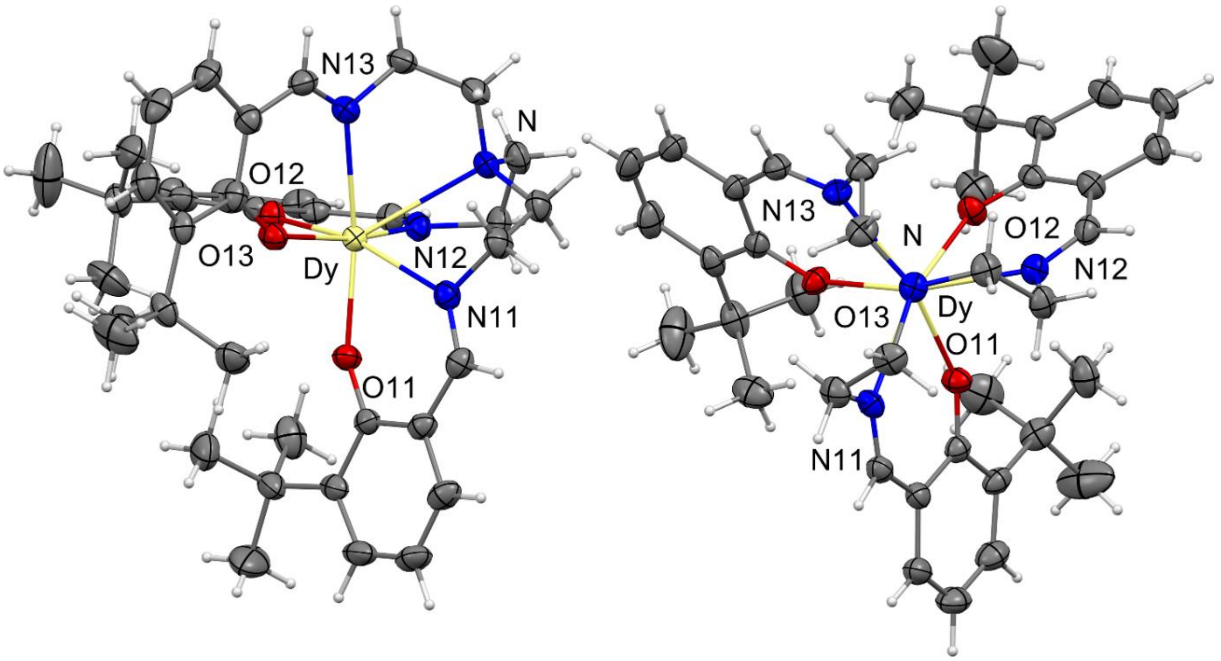

Figure S23. Representation of the structure of **3**. Thermal ellipsoids were depicted at the 30% probability level. Capped octahedron molecular geometry (left). View along the central axis N-Dy (right). Dy (yellow), N (blue) and O (red), C (gray) and H (light gray). Crystallization solvent acetone was omitted for clarity.

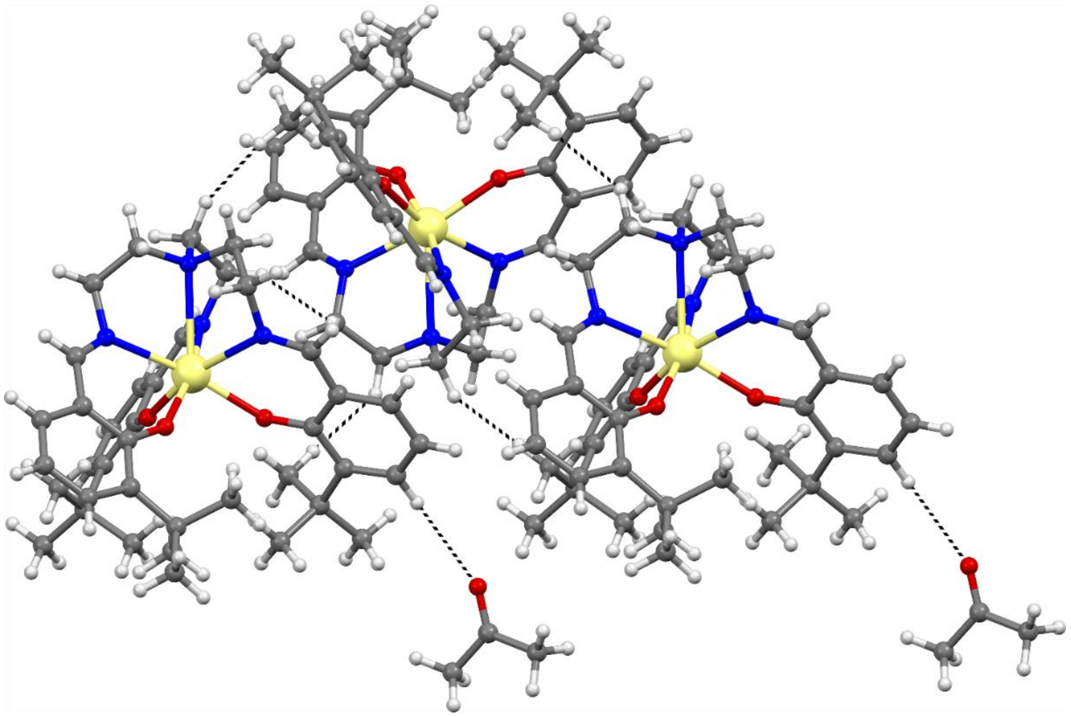

Figure S24. Portion of the crystal packing of **3**·Acetone. Interactions between the complex and acetone are highlighted (dashed bonds). Dy (pale-yellow), N (blue), O (red), C (gray) and H (light gray).

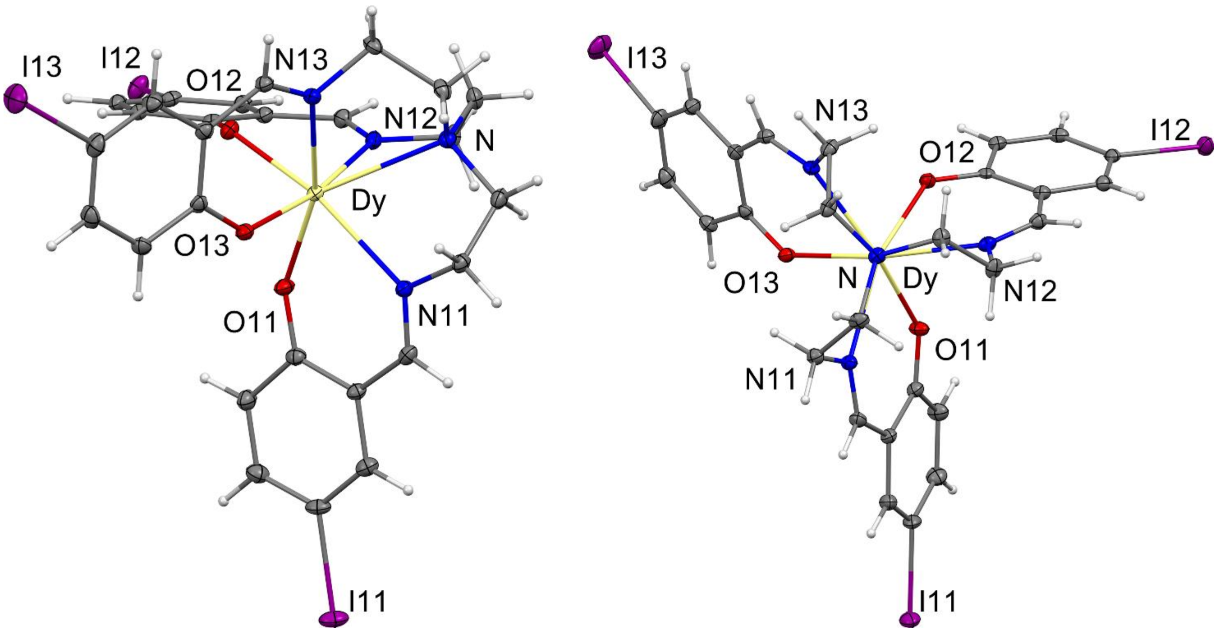

Figure S25. Representation of the structure of **4**. Thermal ellipsoids were depicted at the 30% probability level. Capped octahedron molecular geometry (left). View along the central axis N-Dy (right). Dy (yellow), N (blue) and O (red), I (purple), C (gray) and H (light gray). Crystallization solvent DMF was omitted for clarity.

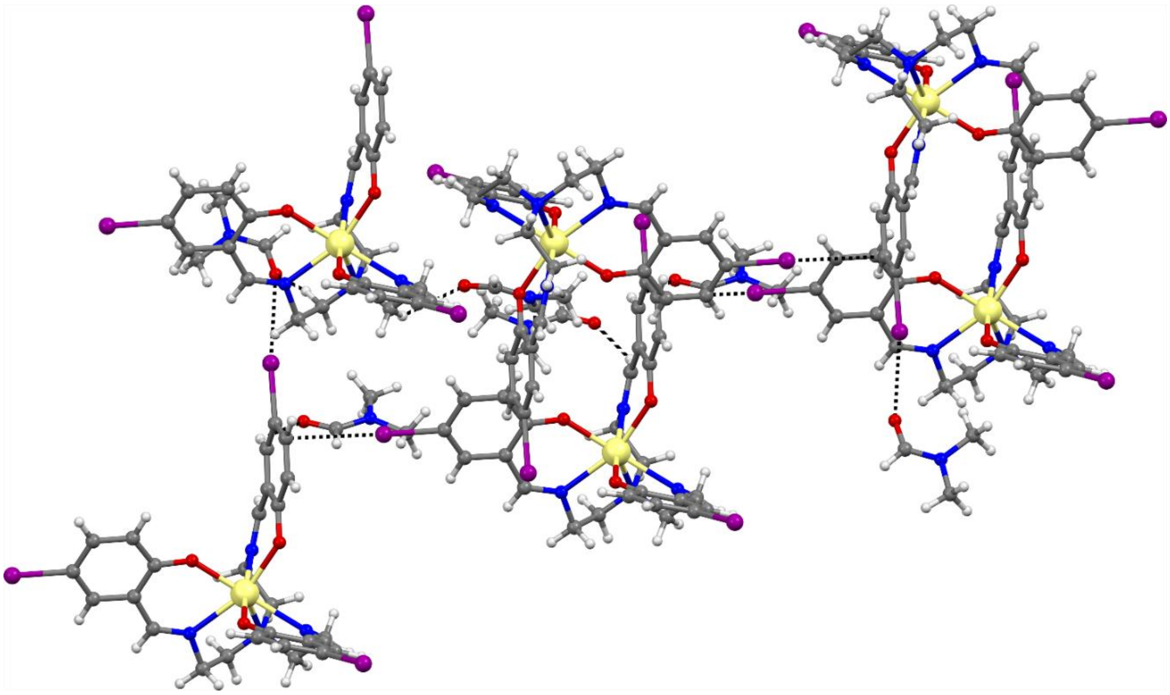

Figure S26. Portion of the crystal packing of **4**·3DMF. C-H···O and halogen bonds with the DMF molecule of crystallization and with symmetry related molecules represent the main interactions (weak interactions as dashed bonds). Dy (pale-yellow), N (blue), O (red), I (purple), C (gray) and H (light gray).

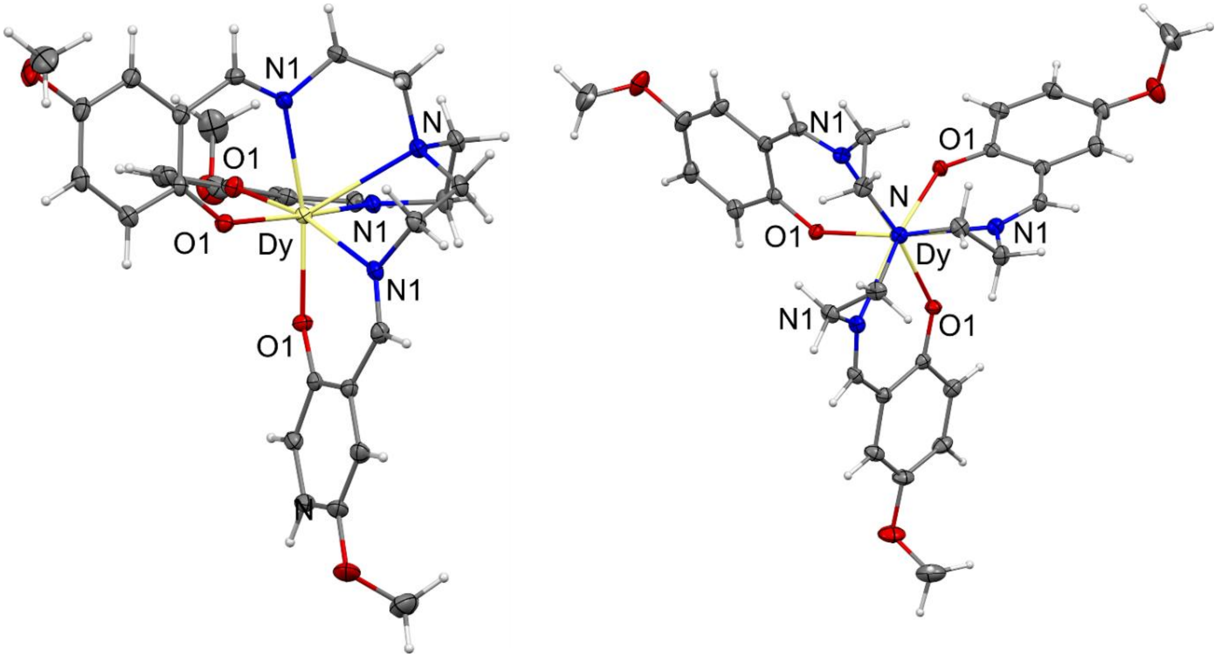

Figure S27. Representation of the structure of **5**. Thermal ellipsoids were depicted at the 30% probability level. Capped octahedron molecular geometry (left). View along the central axis N-Dy (right). Dy (yellow), N (blue) and O (red), C (gray) and H (light gray).

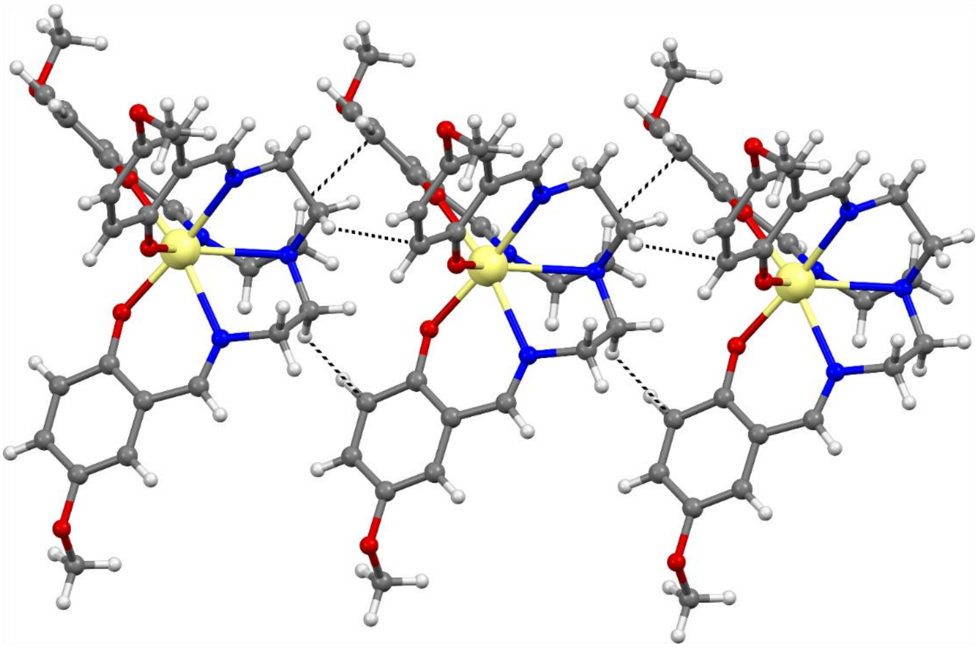

Figure S28. Portion of the crystal packing of **5**. Dashed bonds highlight the C-H···π interactions. Dy (pale-yellow), N (blue), O (red), C (gray) and H (light gray).

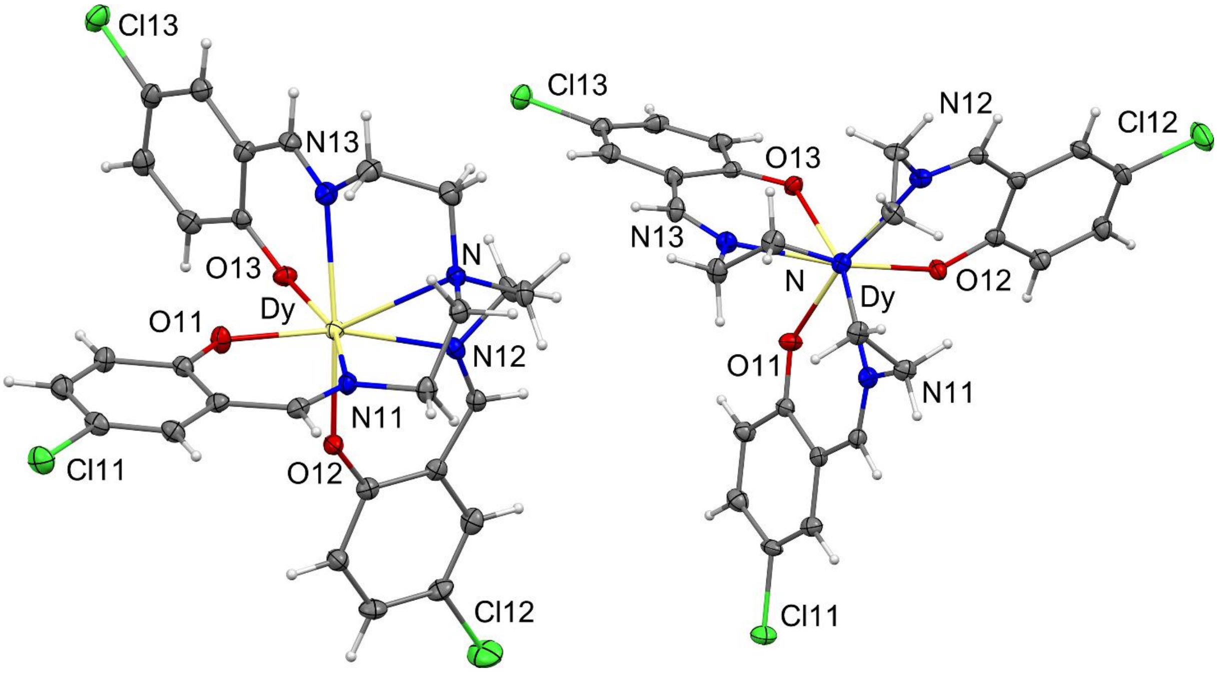

Figure S29. Representation of the structure of **6**. Thermal ellipsoids were depicted at the 30% probability level. Capped octahedron molecular geometry (left). View along the central axis N-Dy (right). Dy (yellow), N (blue) and O (red), Cl (green), C (gray) and H (light gray).

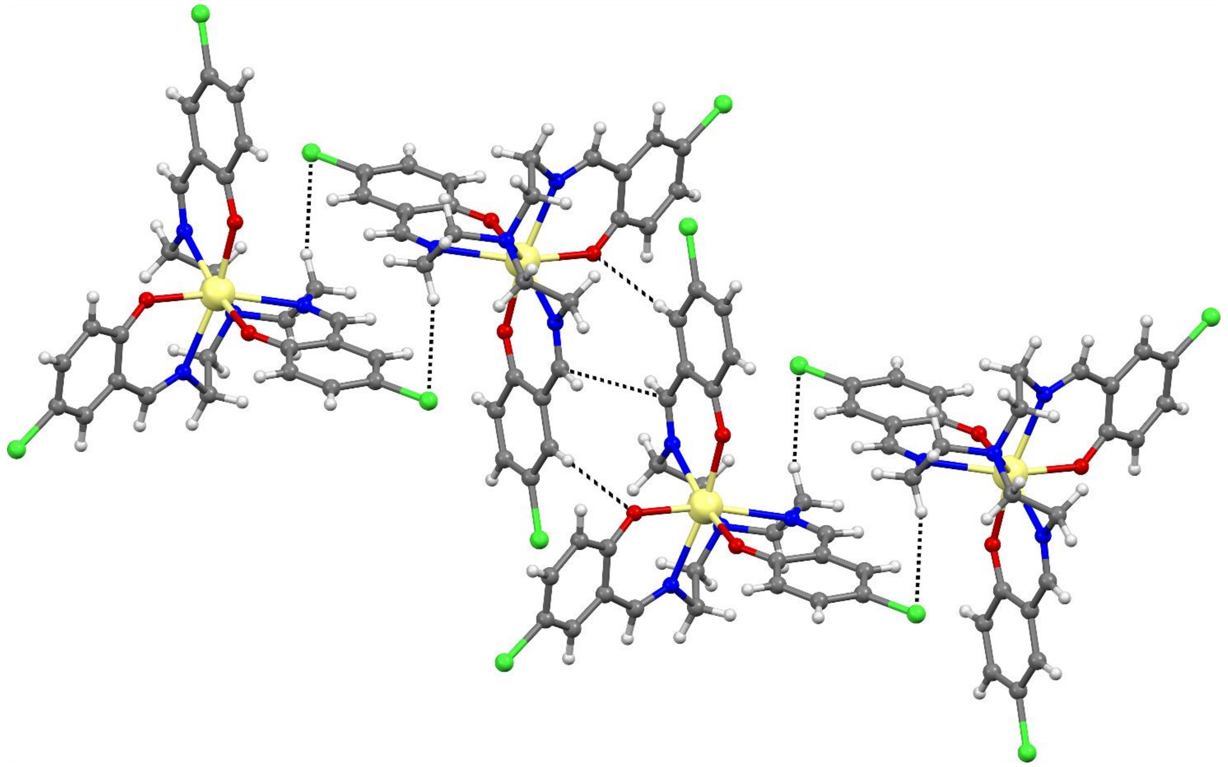

Figure S30. Portion of the crystal packing of **6**. Dashed bonds highlight C-H···O and C-H···Cl interactions. Dy (pale-yellow), N (blue), O (red), Cl (green), C (gray) and H (light gray).

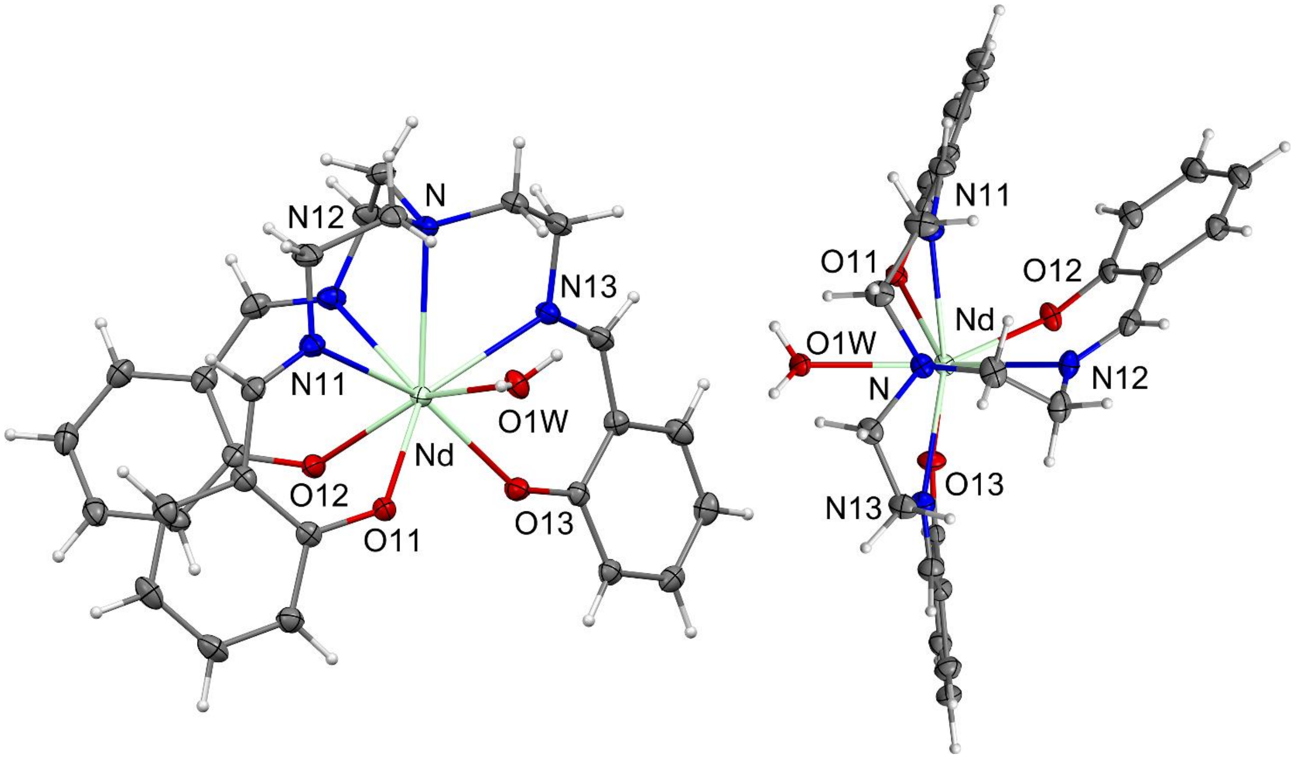

Figure S31. Representation of the structure of **7**. Thermal ellipsoids were depicted at the 30% probability level. Antiprismatic geometry (left). View along the central axis N-Nd (right). Nd (light green), N (blue) and O (red), C (gray) and H (light gray).

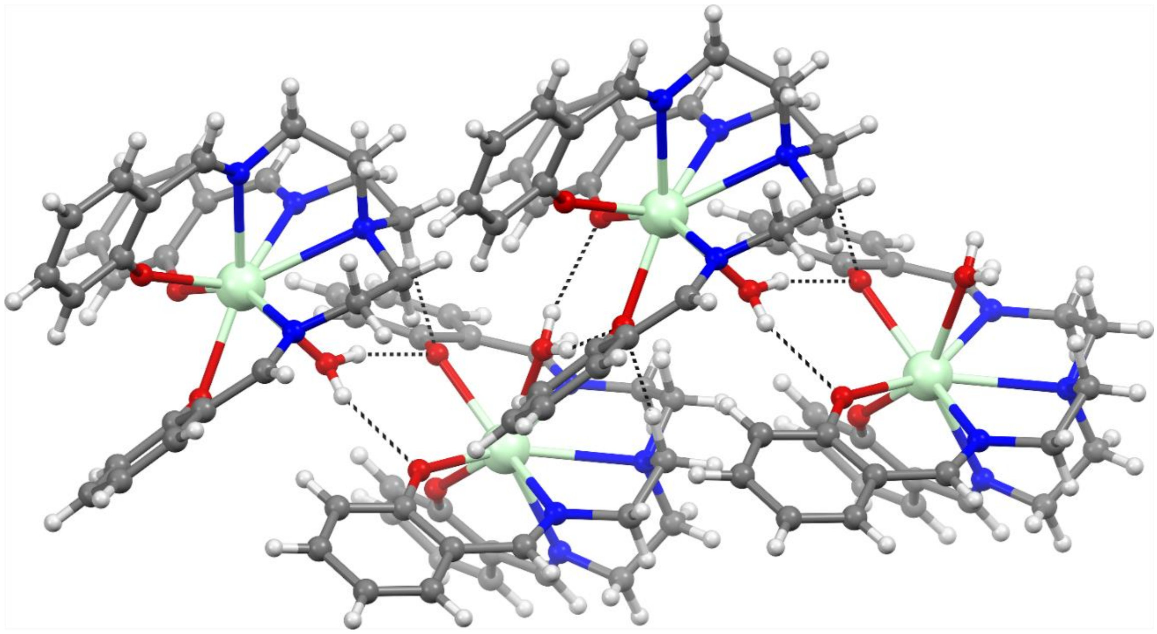

Figure S32. Portion of the crystal packing of **7**. The main interactions are represented by hydrogen bonds between coordinated water and the oxygen atoms of symmetry related molecules. Nd (pale-green), N (blue), O (red), C (gray) and H (light gray).

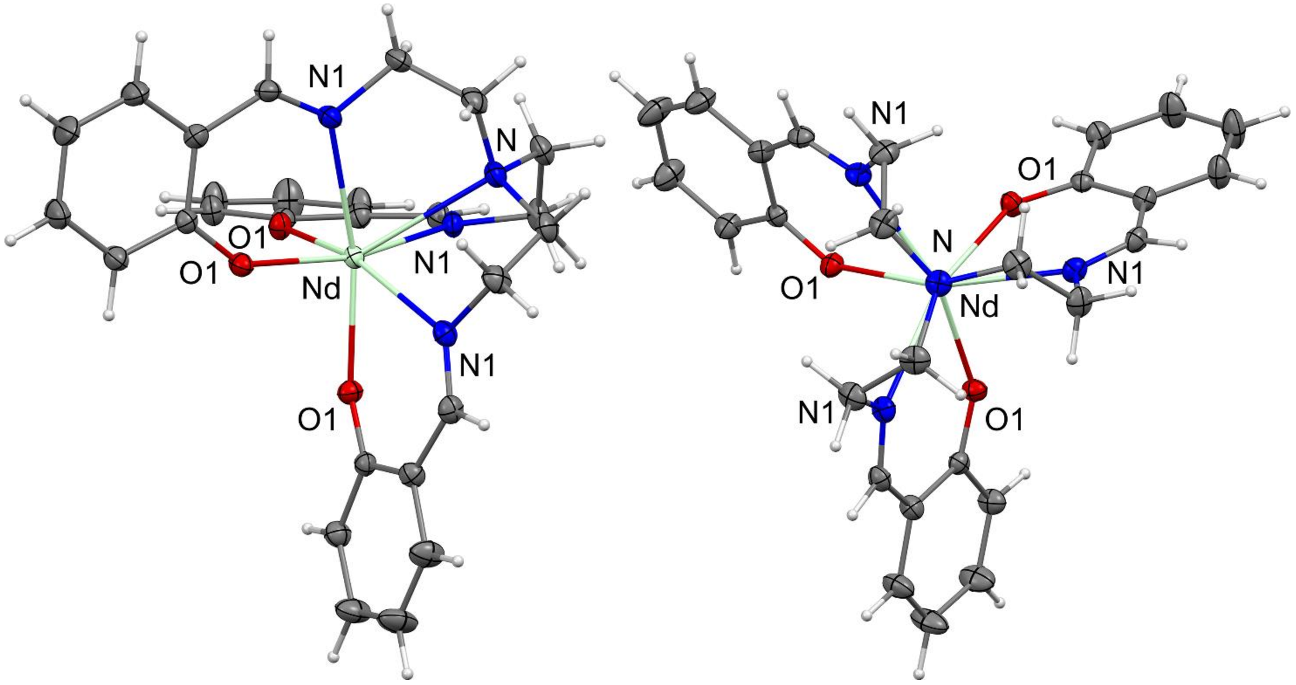

Figure S33. Representation of the structure of **8**. Thermal ellipsoids were depicted at the 30% probability level. Capped octahedron molecular geometry (left). View along the central axis N-Nd (right). Nd (light green), N (blue) and O (red), C (gray) and H (light gray). Crystallization solvent ACN was omitted for clarity.

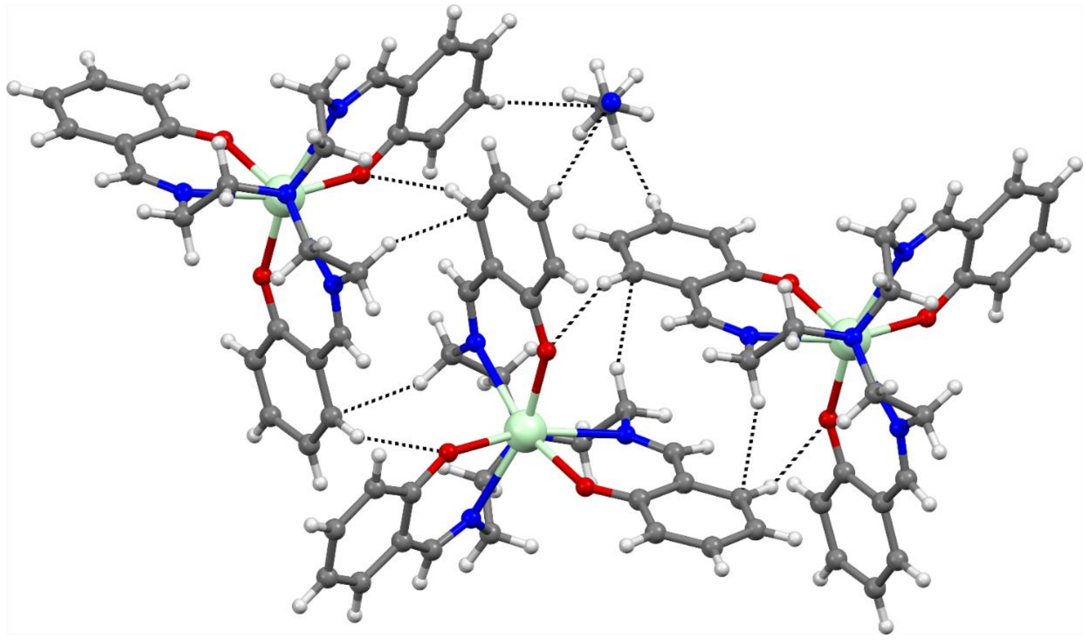

Figure S34. Portion of the crystal packing of **8**·ACN. C-H···O and C-H···π interactions are reported as dashed bonds. Nd (pale-green), N (blue), O (red), C (gray) and H (light gray).

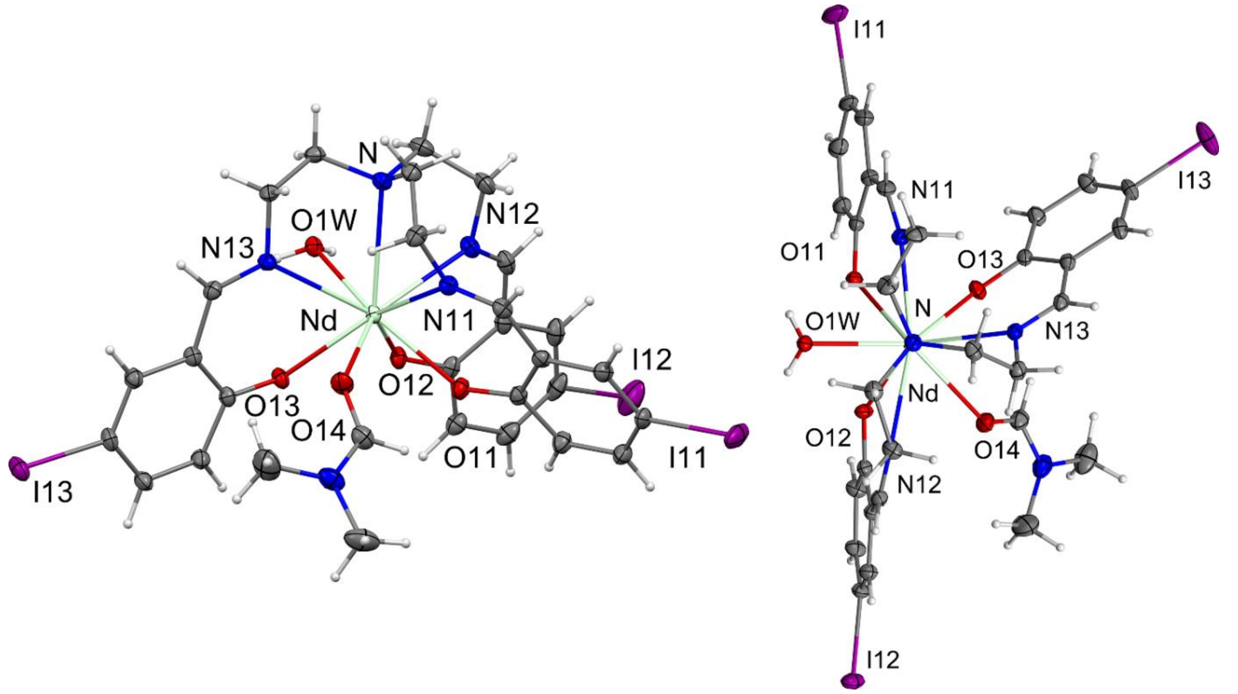

Figure S35. Representation of the structure of **9**·DMF. Thermal ellipsoids were depicted at the 30% probability level. Capped octahedron molecular geometry (left). View along the central axis N-Nd (right). Nd (light green), N (blue) and O (red), I (purple), C (gray) and H (light gray).

Crystallization solvent DMF was omitted for clarity.

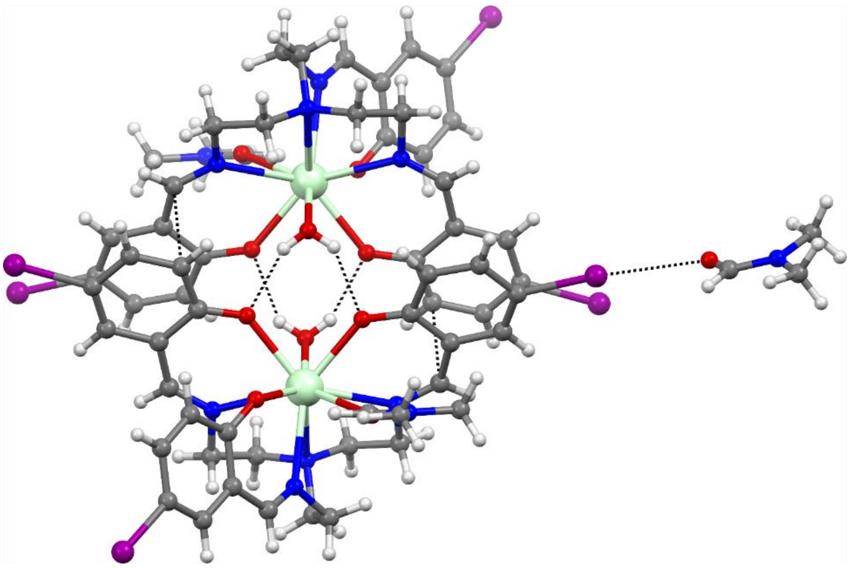

Figure S36. Portion of the crystal packing of **9**·DMF. Hydrogen bonds exchanged by coordinated water, and halogen bonds with DMF molecules of crystallization are reported as dashed bonds. Nd (pale-green), N (blue), O (red), I (purple), C (gray) and H (light gray).

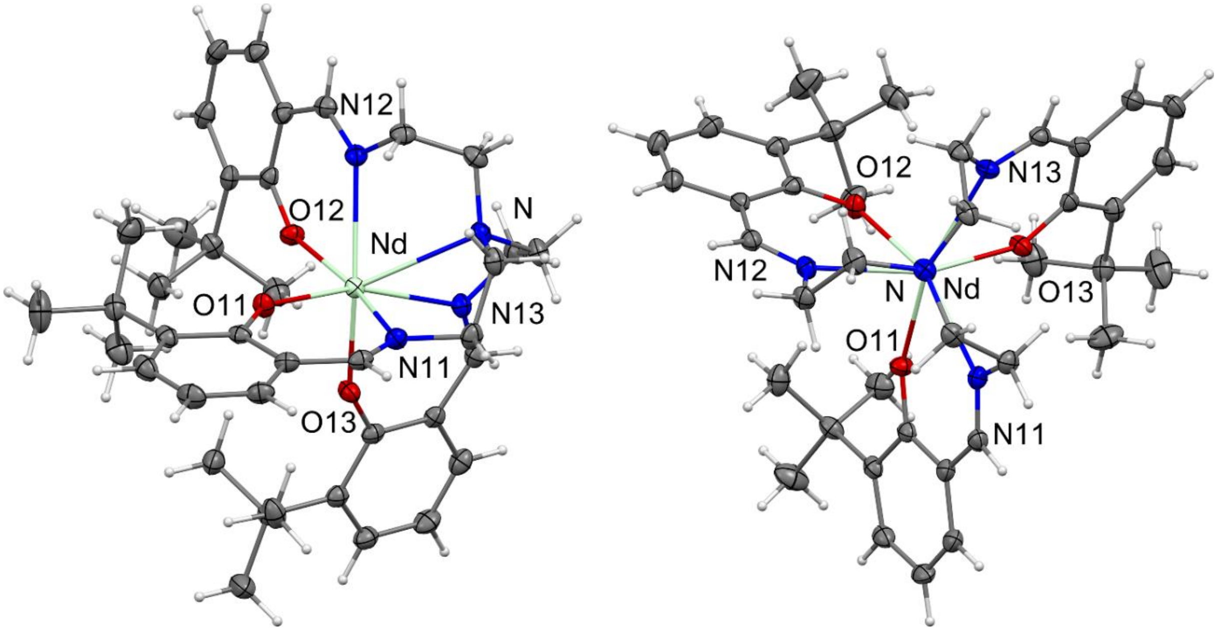

Figure S37. Representation of the structure of **10**. Thermal ellipsoids were depicted at the 30% probability level. Capped octahedron molecular geometry (left). View along the central axis N-Nd (right). Nd (light green), N (blue) and O (red), C (gray) and H (light gray). Crystallization solvent DMF was omitted for clarity.

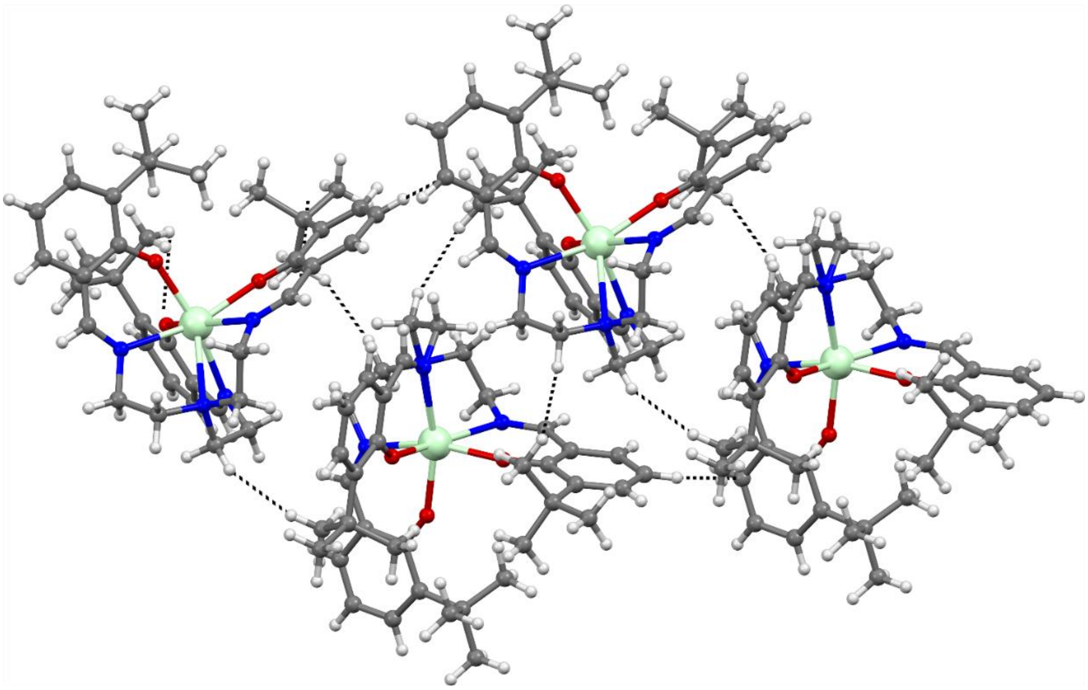

Figure S38. Portion of the crystal packing of **10**. Weak interactions mainly involve C-H groups (dashed bonds). Nd (pale-green), N (blue), O (red), C (gray) and H (light gray).

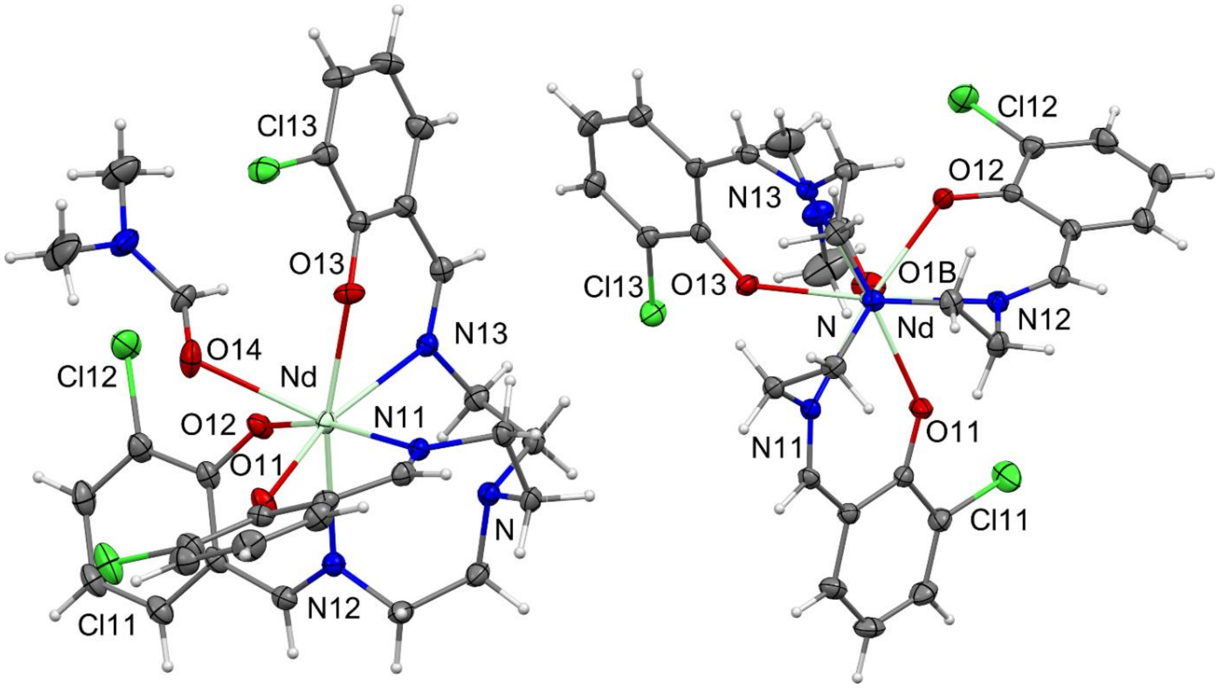

Figure S39. Representation of the structure of **11**. Thermal ellipsoids were depicted at the 30% probability level. Capped octahedron molecular geometry (left). View along the central axis N-Nd (right). Nd (light green), N (blue) and O (red), Cl (green), C (gray) and H (light gray). Crystallization solvent DMF was omitted for clarity.

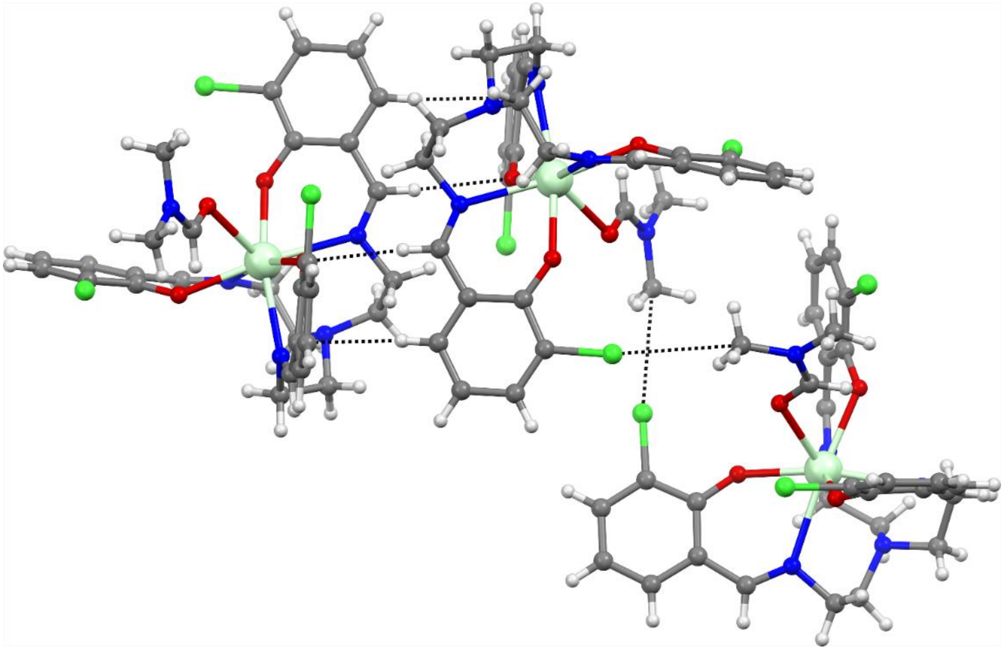

Figure S40. Portion of the crystal packing of **11**. Dashed bonds highlight C-H···O interactions tetrel Cl···CH_3 bonds. Nd (pale-green), N (blue), O (red), Cl (green), C (gray) and H (light gray).

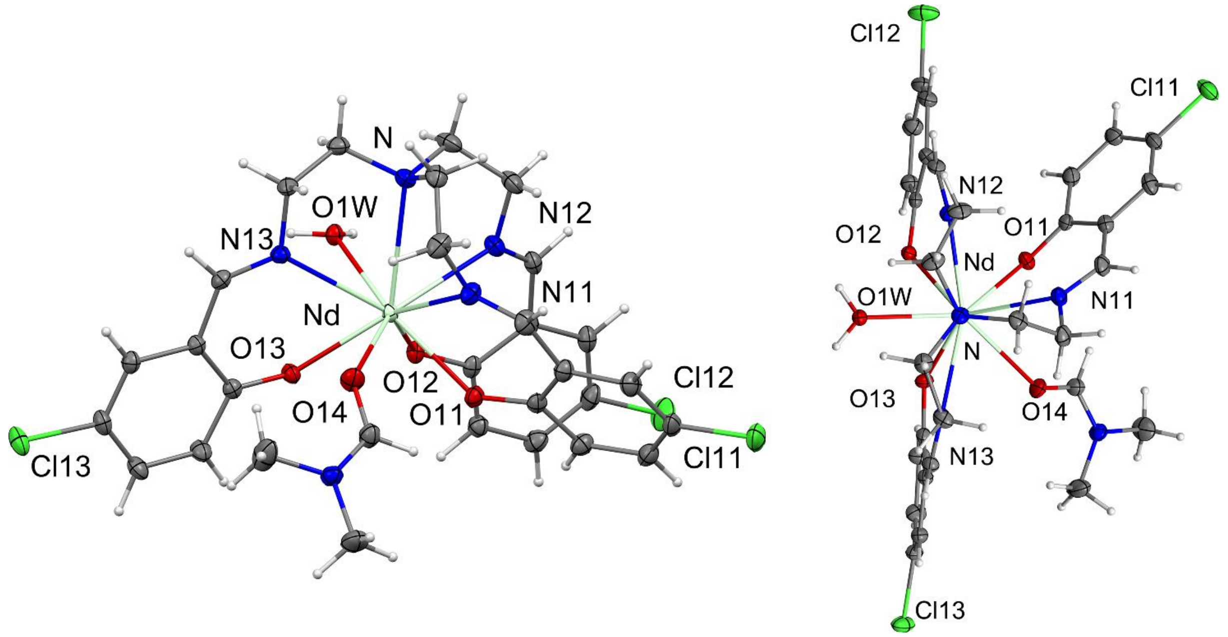

Figure S41. Representation of the structure of **12**. Thermal ellipsoids were depicted at the 30% probability level. Capped octahedron molecular geometry (left). View along the central axis N-Nd (right). Nd (light green), N (blue) and O (red), Cl (green), C (gray) and H (light gray).

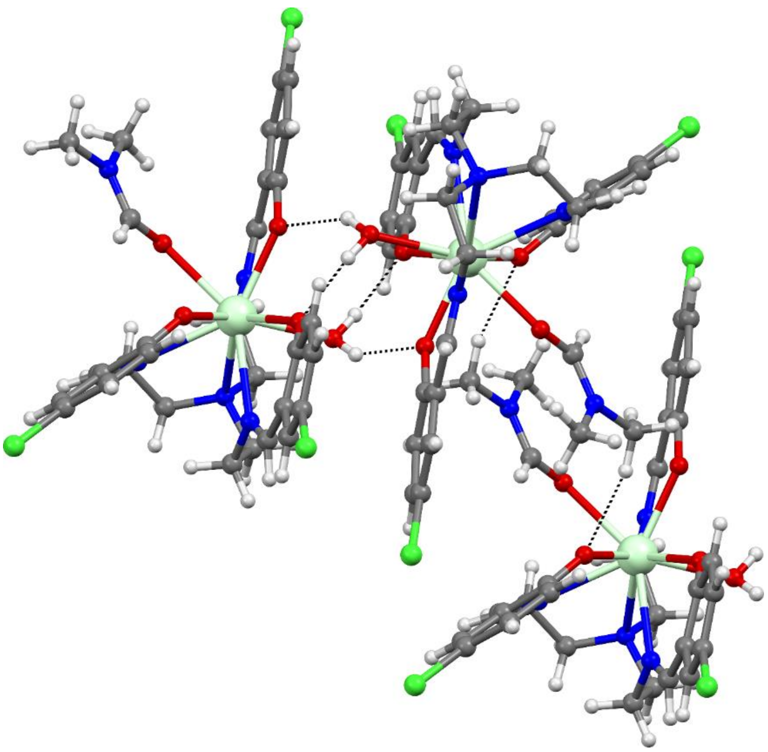

Figure S42. Portion of the crystal packing of **12**. Hydrogen bonds exchanged by coordinated water, and CH···O interactions are reported as dashed bonds. Nd (pale-green), N (blue), O (red), Cl (green), C (gray) and H (light gray).

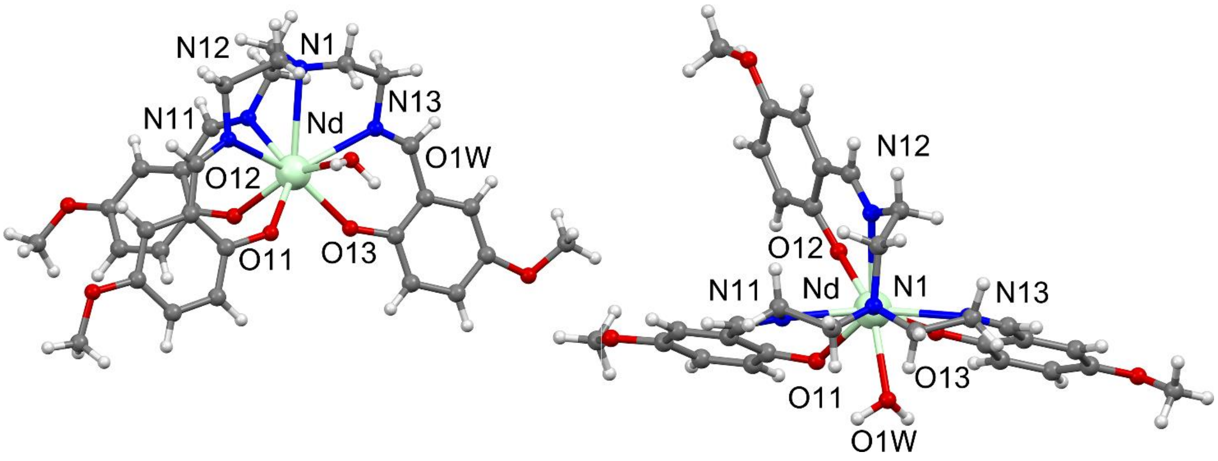

Figure S43. Ball and stick representation of the structure of **13**. Antiprismatic geometry (left). View along the central axis N-Nd (right). Nd (light green), N (blue) and O (red), C (gray) and H (light gray).

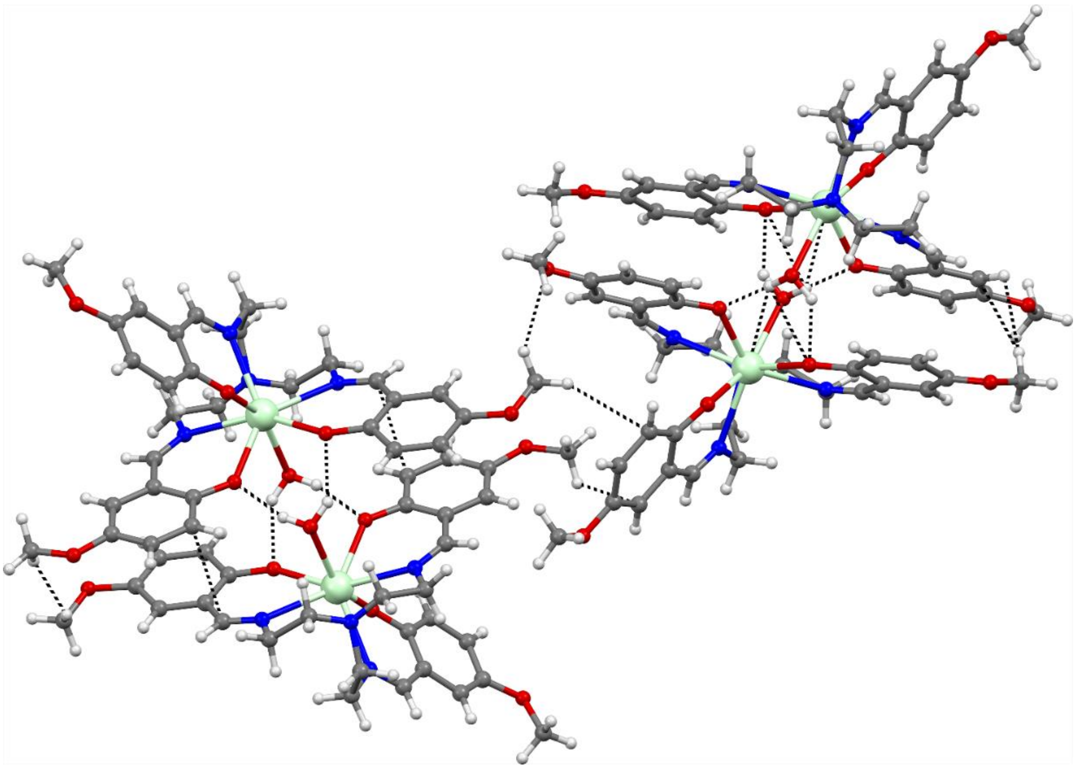

Figure S44. Portion of the crystal packing of **13**. Hydrogen bonds exchanged by coordinated water, and CH···O interactions are reported as dashed bonds. Nd (pale-green), N (blue), O (red), C (gray) and H (light gray).

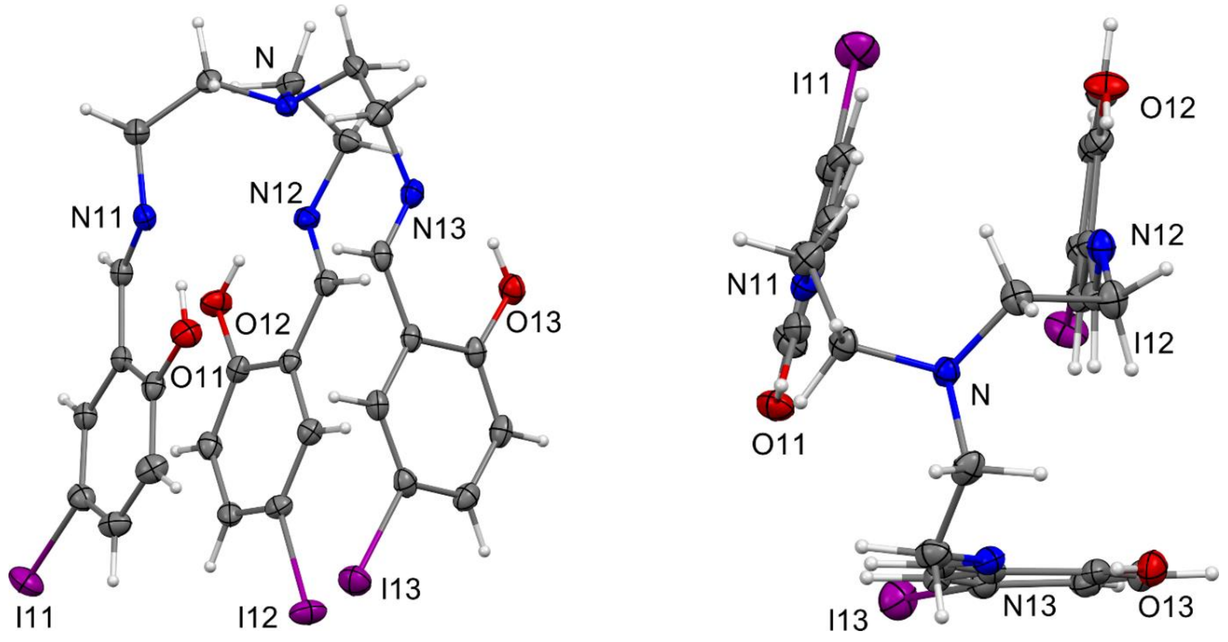

Figure S45. Representation of the structure of **L**$_5$. Thermal ellipsoids were depicted at the 30% probability level. N (blue) and O (red), I (purple), C (gray) and H (light gray).

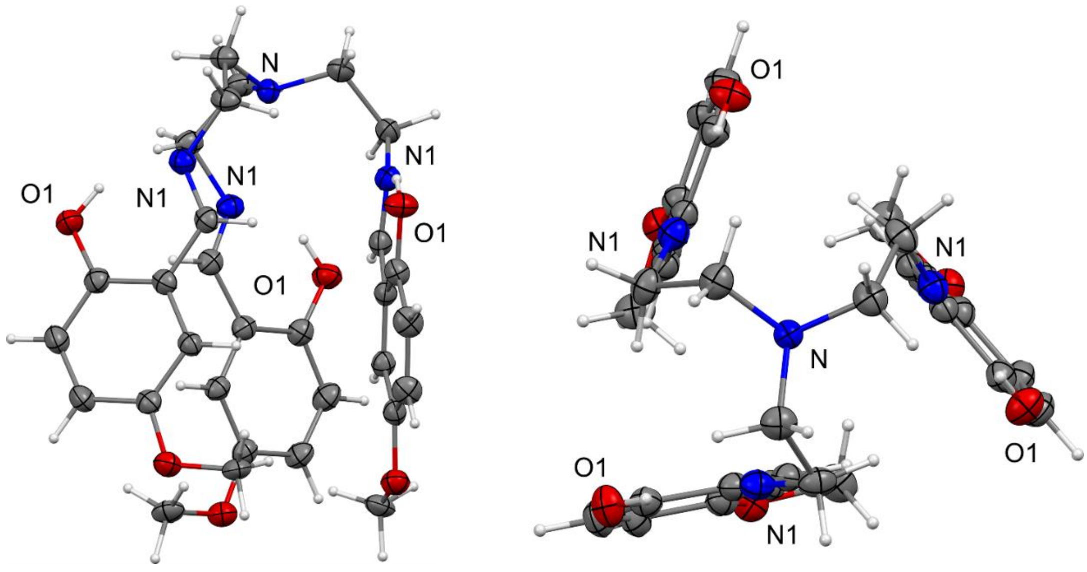

Figure S46. Representation of the structure of **L**$_2$. Thermal ellipsoids were depicted at the 30% probability level. N (blue) and O (red), C (gray) and H (light gray).

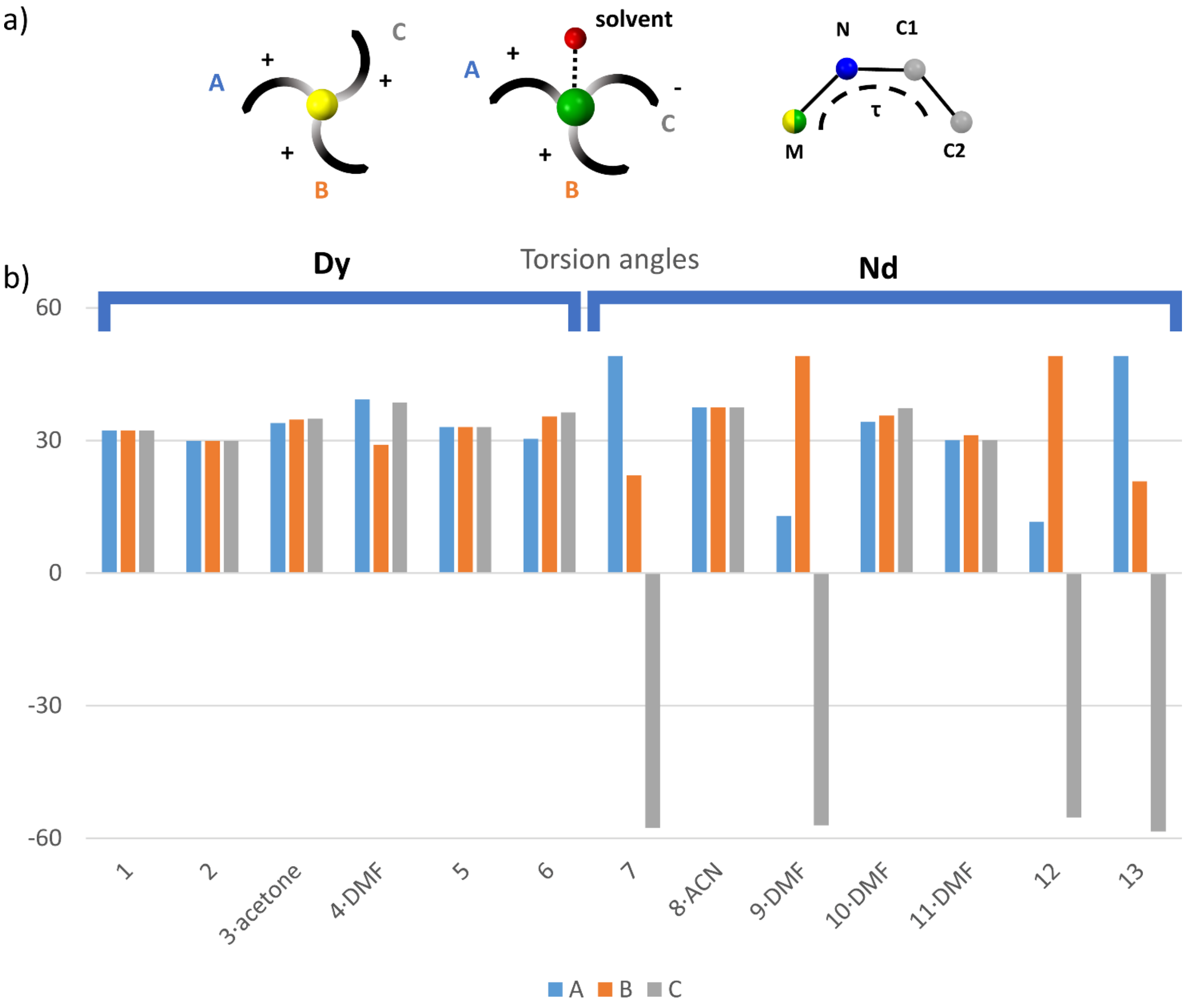

Figure S47. a) Different orientations of the three arms of the ligands (labeled A, B and C) as a function of the sign of the τ angle (M-$N_{central}$-C1-C2). b) Torsion angles (τ: M-$N_{central}$-C1-C2, reported in degrees) for the ethylene conformation of the three arms of the ligands. **7**, **9**·DMF, **12** and **13** have a water molecule coordinated to the metal, and they present a negative value for τ for one of the three residues.

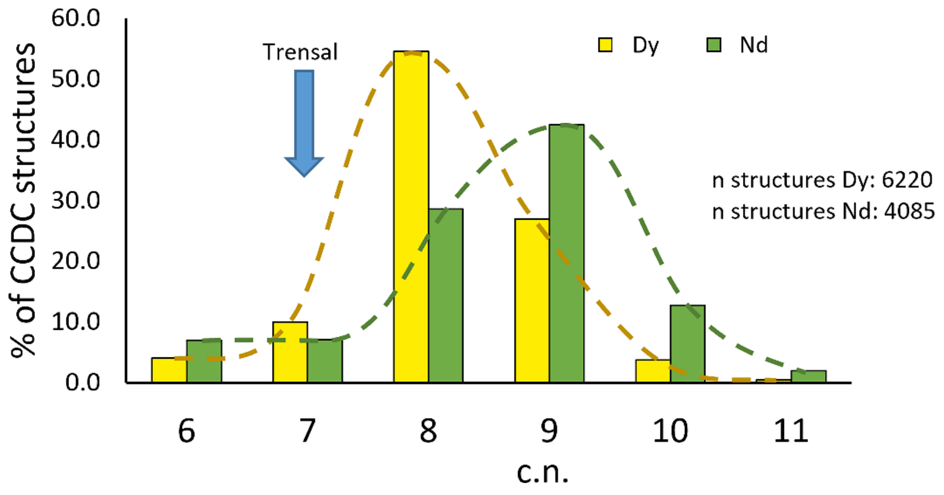

Figure S48. Percentage of Nd and Dy-containing structures deposited in the CCDC *vs* coordination number of the metal (c.n.). A clear trend can be observed where the preferred coordination number for Dy is 8 whereas it is 9 for Nd. Trensal is a heptadentate ligand with a N_4O_3 donor set.

ICP-AES analysis

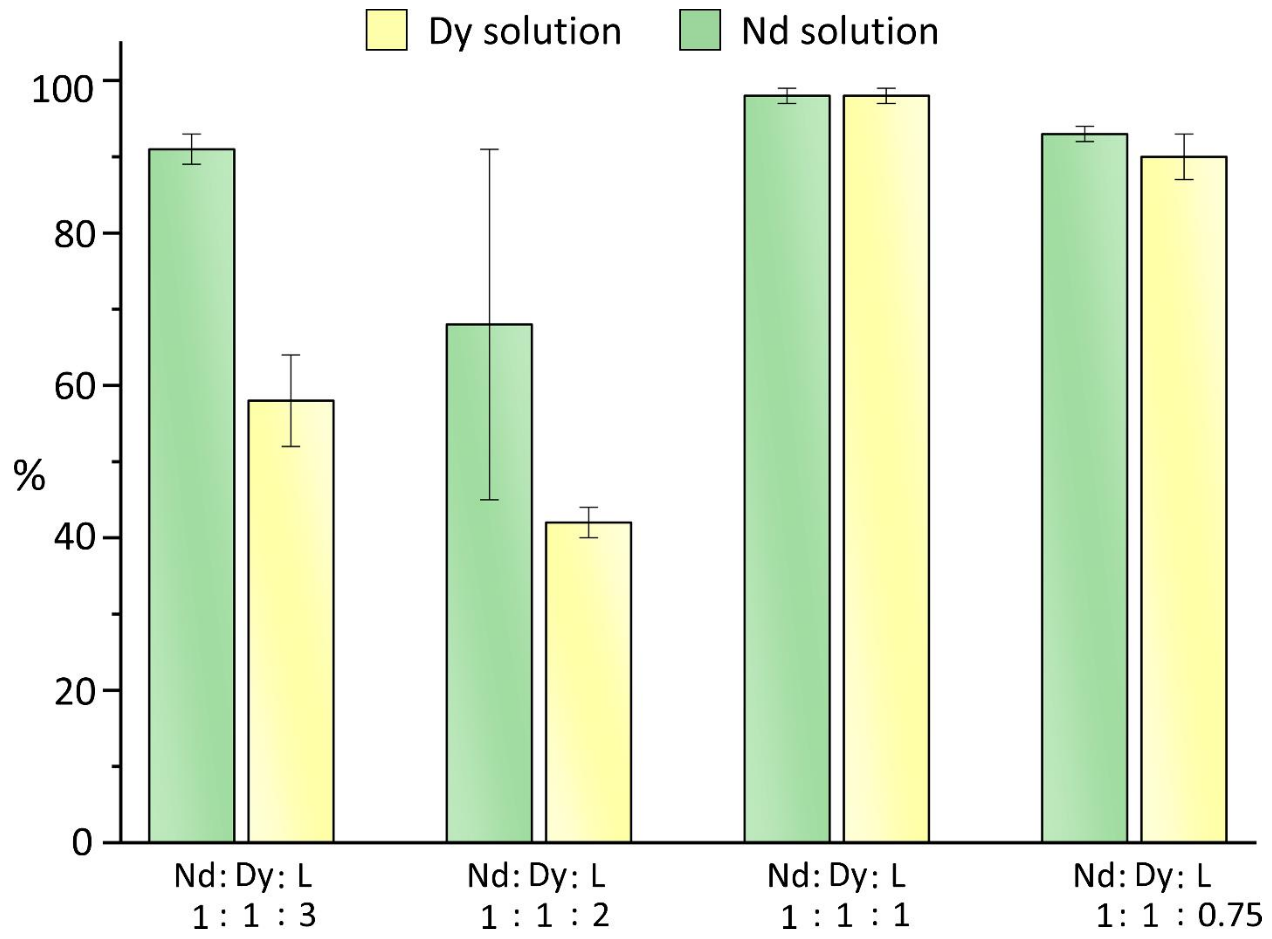

Figure S49. Amount of Nd and Dy as derived by the ICP-AES analysis, recovered in the solution phase on varying the Trensal$^{p\text{-}OMe}$:Ln stochiometric ratios. 3 equivalents of triethylamine with respect to Trensal$^{p\text{-}OMe}$ were used for every conditions. The indicated values are referenced with respect to the initial quantities of metals.

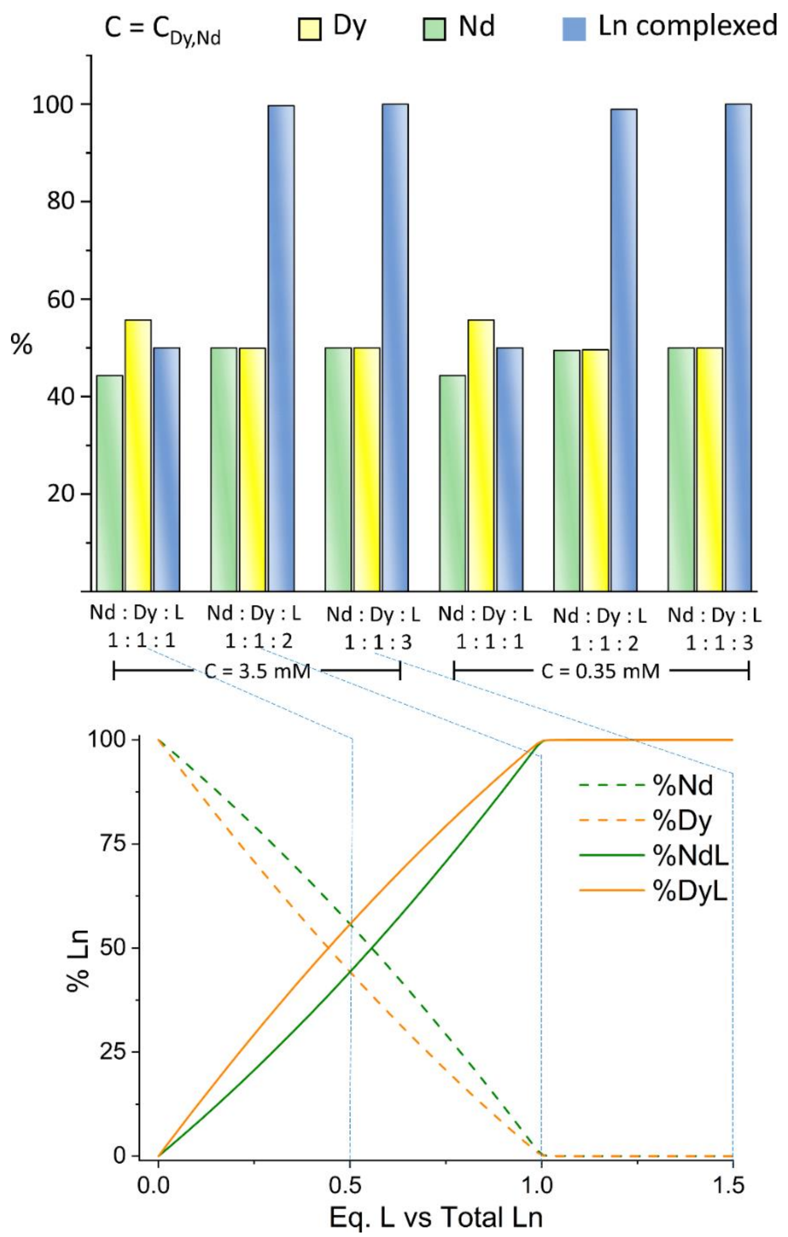

Figure S50. Above, Speciation of the [Nd(Trensal$^{p\text{-}OMe}$)] and [Dy(Trensal$^{p\text{-}OMe}$)] complexes (as % Ln) as a function of the different stochiometric ratio Ln:Trensal$^{p\text{-}OMe}$. The green and yellow bars represent the % of the [Nd(Trensal$^{p\text{-}OMe}$)] and [Dy(Trensal$^{p\text{-}OMe}$)] complexes, respectively, for the specified Nd:Dy:L stoichiometry; The blue bar represents the % of total [Ln(Trensal$^{p\text{-}OMe}$)] complexes for the specified Nd:Dy:L stoichiometry. Only when the ligand is in defect with respect to the metals (1:1:1 stoichiometry), the concentration of [Dy(Trensal$^{p\text{-}OMe}$)] is greater than that of [Nd(Trensal$^{p\text{-}OMe}$)]. Below, representative distribution diagram of a system Nd:Dy:L = 1:1:3 (Total Ln:L = 1:0-1.5; CL = 3.5 10^{-3} M). The reported values are based on the formation constant of the Ln:L 1:1 species determined in acetone.

Crystal data and structural geometric parameters

Table S2. Summary of X-ray crystallographic data for **1**, **2** and **3**·Acetone.

**Identification code**	[Dy(Trensal)][a]	[Dy(Trensal$^{o\text{-}Cl}$)]	[Dy(Trensal$^{o\text{-}tBu}$)]·Acetone
Empirical formula	$C_{27}H_{27}DyN_4O_3$	$C_{27}H_{24}Cl_3DyN_4O_3$	$C_{42}H_{57}DyN_4O_4$
Formula weight	618.02	721.36	844.41
Temperature/K	200.0	200.0	200
Crystal system	trigonal	trigonal	monoclinic
Space group	P-3c1	P-3	C2/c
a/Å	13.0116(4)	13.9712(6)	21.5004(8)
b/Å	13.0116(4)	13.9712(6)	12.1501(4)
c/Å	16.3938(5)	7.8155(5)	31.5994(10)
α/°	90	90	90
β/°	90	90	95.7190(10)
γ/°	120	120	90
Volume/Å^3	2403.65(16)	1321.16(14)	8213.7(5)
Z	4	2	8
ρ_{calc}g/cm^3	1.708	1.813	1.366
μ/mm^{-1}	3.146	18.237	1.863
F(000)	1228.0	710.0	3480.0
Crystal size/mm^3	0.18 × 0.11 × 0.08	0.19 × 0.04 × 0.04	0.21 × 0.15 × 0.13
Radiation	MoKα (λ = 0.71073)	CuKα (λ = 1.54178)	MoKα (λ = 0.71073)
2Θ range for data collection/°	6.146 to 51.388	7.306 to 139.936	3.856 to 52.104
Index ranges	$-15 \le h \le 15$, $-15 \le k \le 15$, $-19 \le l \le 19$	$-16 \le h \le 16$, $-17 \le k \le 15$, $-9 \le l \le 9$	$-26 \le h \le 26$, $-14 \le k \le 14$, $-39 \le l \le 39$
Reflections collected	52201	10103	40218
Independent reflections	1523 [R_{int} = 0.0430, R_{sigma} = 0.0117]	1666 [R_{int} = 0.1465, R_{sigma} = 0.0873]	8089 [R_{int} = 0.0351, R_{sigma} = 0.0285]
Data/restraints/parameters	1523/0/106	1666/0/115	8089/0/471
Goodness-of-fit on F^2	1.049	1.003	1.003
Final R indexes [I>=2σ (I)]	R_1 = 0.0177, wR_2 = 0.0498	R_1 = 0.0423, wR_2 = 0.0901	R_1 = 0.0369, wR_2 = 0.1244
Final R indexes [all data]	R_1 = 0.0197, wR_2 = 0.0527	R_1 = 0.0666, wR_2 = 0.1004	R_1 = 0.0429, wR_2 = 0.1301
Largest diff. peak/hole / e Å^{-3}	0.33/-1.38	1.55/-0.88	1.30/-2.37

[a] Structure already present in the database as CCDC 618519.

Table S3. Summary of X-ray crystallographic data for **4**·3DMF, **5** and **6**.

**Identification code**	[Dy(Trensal^{p-I})]·3DMF	[Dy(Trensal^{p-OMe})]	[Dy(Trensal^{p-Cl})]
Empirical formula	$C_{36}H_{45}DyI_3N_7O_6$	$C_{30}H_{33}DyN_4O_6$	$C_{27}H_{24}Cl_3DyN_4O_3$
Formula weight	1214.99	708.11	721.35
Temperature/K	150.0	200.0	200.0
Crystal system	triclinic	trigonal	monoclinic
Space group	P-1	P-3	$P2_1/n$
a/Å	13.0475(5)	14.7962(8)	12.6245(7)
b/Å	13.4115(5)	14.7962(8)	15.2077(7)
c/Å	15.1120(6)	7.6514(8)	15.0627(9)
α/°	73.4640(10)	90	90
β/°	65.4600(10)	90	110.359(2)
γ/°	62.1540(10)	120	90
Volume/Å^3	2113.45(14)	1450.7(2)	2711.2(3)
Z	2	2	4
ρ_{calc}g/cm^3	1.909	1.621	1.767
μ/mm^{-1}	4.009	2.625	3.089
F(000)	1166.0	710.0	1420.0
Crystal size/mm^3	0.4 × 0.05 × 0.04	0.22 × 0.19 × 0.15	0.21 × 0.2 × 0.15
Radiation	MoKα (λ = 0.71073)	MoKα (λ = 0.71073)	MoKα (λ = 0.71073)
2Θ range for data collection/°	3.934 to 52.84	5.324 to 51.502	3.936 to 51.5
Index ranges	$-16 \le h \le 16, -16 \le k \le 16, -18 \le l \le 18$	$-18 \le h \le 18, -18 \le k \le 16, -9 \le l \le 9$	$-15 \le h \le 15, -18 \le k \le 17, -18 \le l \le 18$
Reflections collected	34347	11634	26851
Independent reflections	8659 [R_{int}= 0.0415, R_{sigma}= 0.0345]	1854 [R_{int} = 0.0501, R_{sigma} = 0.0284]	5166 [R_{int}= 0.0635, R_{sigma}= 0.0502]
Data/restraints/parameters	8659/56/532	1854/0/125	5166/0/343
Goodness-of-fit on F^2	1.017	1.073	1.056
Final R indexes [I>=2σ (I)]	R_1= 0.0245, wR_2= 0.0471	R_1 = 0.0195, wR_2 = 0.0421	R_1= 0.0350, wR_2= 0.0740
Final R indexes [all data]	R_1= 0.0310, wR_2= 0.0493	R_1 = 0.0226, wR_2 = 0.0434	R_1= 0.0492, wR_2= 0.0810
Largest diff. peak/hole / e Å^{-3}	1.33/-1.05	0.51/-0.57	1.66/-0.86

Table S4. Summary of X-ray crystallographic data for **7**, **8**·ACN and **9**·DMF.

**Identification code**	[Nd(Trensal)(H_2O)]	[Nd(Trensal)]·ACN[a]	[Nd(Trensal$^{p\text{-}I}$)DMF(H_2O)]·DMF
Empirical formula	$C_{27}H_{29}N_4NdO_4$	$C_{28}H_{28}N_{4.5}NdO_3$	$C_{33}H_{40}I_3N_6NdO_6$
Formula weight	617.78	619.79	1141.65
Temperature/K	220	160.0	200.0
Crystal system	monoclinic	trigonal	triclinic
Space group	$P2_1/n$	P-3	P-1
a/Å	11.3824(2)	13.5381(7)	12.7307(19)
b/Å	8.55910(10)	13.5381(7)	13.1608(12)
c/Å	24.9240(4)	8.1270(4)	13.8742(15)
α/°	90	90	98.604(4)
β/°	93.551(2)	90	106.887(4)
γ/°	90	120	112.474(4)
Volume/Å^3	2423.51(6)	1289.96(15)	1964.1(4)
Z	4	2	2
ρ_{calc}g/cm^3	1.693	1.597	1.930
μ/mm^{-1}	2.185	2.051	3.728
F(000)	1244.0	624.0	1094.0
Crystal size/mm^3	0.3 × 0.03 × 0.03	0.21 × 0.19 × 0.12	0.12 × 0.03 × 0.018
Radiation	MoKα (λ = 0.71073)	MoKα (λ = 0.71073)	MoKα (λ = 0.71073)
2Θ range for data collection/°	3.848 to 51.362	6.1 to 51.48	3.806 to 51.356
Index ranges	$-13 \leq h \leq 13$, $-10 \leq k \leq 9$, $-30 \leq l \leq 30$	$-16 \leq h \leq 16$, $-14 \leq k \leq 16$, $-9 \leq l \leq 9$	$-15 \leq h \leq 15$, $-15 \leq k \leq 16$, $-16 \leq l \leq 16$
Reflections collected	28747	10121	30719
Independent reflections	4598 [R_{int}= 0.0338, R_{sigma}= 0.0226]	1641 [R_{int}= 0.0247, R_{sigma}= 0.0159]	7422 [R_{int}= 0.0282, R_{sigma}= 0.0236]
Data/restraints/parameters	4598/2/331	1641/0/115	7422/2/454
Goodness-of-fit on F^2	1.038	1.023	1.016
Final R indexes [I>=2σ (I)]	R_1= 0.0217, wR_2 = 0.0560	R_1= 0.0136, wR_2 = 0.0368	R_1= 0.0276, wR_2 = 0.0613
Final R indexes [all data]	R_1= 0.0258, wR_2 = 0.0581	R_1= 0.0136, wR_2 = 0.0368	R_1= 0.0305, wR_2 = 0.0629
Largest diff. peak/hole / e Å^{-3}	0.37/-0.49	0.26/-0.60	3.17/-2.50

[a] Structure already present in the database as CCDC 1272122.

Table S5. Summary of X-ray crystallographic data for **10**·DMF, **11**·DMF, **12** and **13**.

**Identification code**	[Nd(Trensal$^{o\text{-}tBu}$)]DMF	[Nd(Trensal$^{o\text{-}Cl}$)(DMF)]·DMF	[Nd(Trensal$^{p\text{-}Cl}$)(DMF)(H_2O)]	[Nd(Trensal$^{p\text{-}OMe}$)(H_2O)]
Empirical formula	$C_{42}H_{58}N_5NdO_4$	$C_{33}H_{38}Cl_3N_6NdO_5$	$C_{30}H_{33}Cl_3N_5NdO_5$	$C_{30}H_{35}N_4NdO_7$
Formula weight	841.17	849.28	794.20	707.86
Temperature/K	200.0	200.0	200.0	200.00(10)
Crystal system	monoclinic	monoclinic	triclinic	monoclinic
Space group	C2/c	C2/c	P-1	$P2_1/c$
a/Å	21.5929(14)	26.3461(7)	11.5773(5)	31.292(5)
b/Å	11.9510(9)	13.1816(3)	11.9645(5)	8.7088(19)
c/Å	31.788(2)	22.3923(6)	13.0394(6)	22.605(4)
α/°	90	90	77.635(2)	90
β/°	93.616(2)	111.1890(10)	81.139(2)	110.27(2)
γ/°	90	90	67.3280(10)	90
Volume/Å^3	8186.9(10)	7250.7(3)	1622.81(12)	5779(2)
Z	8	8	2	8
ρ_{calc}g/cm^3	1.365	1.556	1.625	1.627
μ/mm^{-1}	1.314	1.701	1.893	14.168
F(000)	3496.0	3432.0	798.0	2872.0
Crystal size/mm^3	0.17 × 0.13 × 0.12	0.25 × 0.19 × 0.11	0.19 × 0.16 × 0.16	0.1 × 0.1 × 0.02
Radiation	MoKα (λ = 0.71073)	MoKα (λ = 0.71073)	MoKα (λ = 0.71073)	CuKα (λ = 1.54184)
2Θ range for data collection/°	3.898 to 51.462	3.71 to 51.426	3.824 to 51.576	6.022 to 108.474
Index ranges	-26 ≤ h ≤ 26, -14 ≤ k ≤ 14, -31 ≤ l ≤ 38	-32 ≤ h ≤ 32, -16 ≤ k ≤ 16, -27 ≤ l ≤ 27	-14 ≤ h ≤ 14, -14 ≤ k ≤ 14, -15 ≤ l ≤ 15	-32 ≤ h ≤ 32, -9 ≤ k ≤ 9, -23 ≤ l ≤ 23
Reflections collected	29087	56760	47397	35749
Independent reflections	7635 [R_{int} = 0.0497, R_{sigma} = 0.0475]	6889 [R_{int} = 0.0339, R_{sigma} = 0.0184]	6203 [R_{int} = 0.0629, R_{sigma} = 0.0323]	6870 [R_{int} = 0.4169, R_{sigma} = 0.2214]
Data/restraints/parameters	7635/0/480	6889/18/476	6203/0/400	3852/570/648
Goodness-of-fit on F^2	1.060	1.043	1.052	1.069
Final R indexes [I>=2σ (I)]	R_1 = 0.0430, wR_2 = 0.1097	R_1 = 0.0199, wR_2 = 0.0452	R_1 = 0.0189, wR_2 = 0.0455	R_1 = 0.1598, wR_2 = 0.3796
Final R indexes [all data]	R_1 = 0.0524, wR_2 = 0.1143	R_1 = 0.0255, wR_2 = 0.0485	R_1 = 0.0213, wR_2 = 0.0464	R_1 = 0.2301, wR_2 = 0.4331
Largest diff. peak/hole / e Å^{-3}	1.42/-1.48	0.48/-0.49	0.56/-0.54	1.79/-1.12

Table S6. Selected geometric parameters (Å) for **1**, **2**, **3**·Acetone, **4**·3DMF, **5**, **6**.

**1**		**2**	
Dy-N	2.728(3)	Dy-N	2.766(7)
Dy-N(1)	2.486(2)	Dy-N(1)	2.462(5)
Dy-O(1)	2.206(1)	Dy-O(1)	2.213(4)
**3**·Acetone		**4**·3DMF	
Dy-N	2.764(4)	Dy-N	2.674(3)
Dy-N(11)	2.495(4)	Dy-N(11)	2.460(2)
Dy-N(12)	2.499(4)	Dy-N(12)	2.469(2)
Dy-N(13)	2.503(4)	Dy-N(13)	2.490(3)
Dy-O(11)	2.189(3)	Dy-O(11)	2.199(2)
Dy-O(12)	2.188(3)	Dy-O(12)	2.222(2)
Dy-O(13)	2.187(3)	Dy-O(13)	2.223(2)
**5**		**6**	
Dy-N	2.691(3)	Dy-N	2.724(4)
Dy-N(1)	2.470(2)	Dy-N(11)	2.474(4)
Dy-O(1)	2.205(2)	Dy-N(12)	2.490(4)
		Dy-N(13)	2.496(4)
		Dy-O(11)	2.203(3)
		Dy-O(12)	2.212(3)
		Dy-O(13)	2.199(3)

Table S7. Selected geometric parameters (Å) for **7**, **8**·ACN, **9**·DMF, **10**·DMF, **11**·DMF, **12**.

**7**		**8**·ACN	
Nd-N	2.776(2)	Nd-N	2.798(2)
Nd-N(11)	2.583(2)	Nd-N(1)	2.596(1)
Nd-N(12)	2.652(2)	Nd-O(1)	2.273(1)
Nd-N(13)	2.677(2)		
Nd-O(11)	2.410(2)		
Nd-O(12)	2.299(2)		
Nd-O(13)	2.360(2)		
Nd-O(1W)	2.523(2)		
**9**·DMF		**10**·DMF	
Nd-N	2.841(3)	Nd-N	2.821(4)
Nd-N(11)	2.647(3)	Nd-N(11)	2.573(5)
Nd-N(12)	2.664(3)	Nd-N(12)	2.590(4)
Nd-N(13)	2.708(3)	Nd-N(13)	2.601(4)
Nd-O(11)	2.352(3)	Nd-O(11)	2.261(3)
Nd-O(12)	2.397(2)	Nd-O(12)	2.248(4)
Nd-O(13)	2.361(2)	Nd-O(13)	2.259(4)
Nd-O(1W)	2.531(3)		
Nd-O(14)	2.588(3)		
**11**·DMF		**12**	
Nd-N	3.012(2)	Nd-N	2.846(2)
Nd-N(11)	2.595(2)	Nd-N(11)	2.626(2)
Nd-N(12)	2.603(2)	Nd-N(12)	2.649(2)
Nd-N(13)	2.622(2)	Nd-N(13)	2.733(2)
Nd-O(11)	2.303(1)	Nd-O(11)	2.349(1)
Nd-O(12)	2.337(1)	Nd-O(12)	2.410(1)
Nd-O(13)	2.320(1)	Nd-O(13)	2.365(1)
Nd-O(14)	2.522(4)	Nd-O(1W)	2.525(1)
Nd-O(14A)	2.460(6)	Nd-O(14)	2.535(1)

Table S8. Selected geometric parameters (Å) for **13**.

**13**			
Nd1-N(1)	2.82(3)	Nd2-N(2)	2.82(2)
Nd1-N(11)	2.58(3)	Nd2-N(14)	2.64(2)
Nd1-N(12)	2.59(3)	Nd2-N(15)	2.61(2)
Nd1-N(13)	2.69(2)	Nd2-N(16)	2.72(2)
Nd1-O(11)	2.38(2)	Nd2-O(14)	2.34(2)
Nd1-O(12)	2.33(3)	Nd2-O(15)	2.24(2)
Nd1-O(13)	2.34(3)	Nd2-O(16)	2.31(2)
Nd1-O(1W)	2.49(2)	Nd2-O(2W)	2.53(2)
